# U5 snRNA Interactions With Exons Ensure Splicing Precision

**DOI:** 10.3389/fgene.2021.676971

**Published:** 2021-07-02

**Authors:** Olga V. Artemyeva-Isman, Andrew C. G. Porter

**Affiliations:** Gene Targeting Group, Centre for Haematology, Department of Immunology and Inflammation, Faculty of Medicine, Imperial College London, London, United Kingdom

**Keywords:** splice sites, splicing mutations, U5 snRNA, U6 snRNA, U2 snRNA, U1 snRNA, group II intron retrotransposition, RNA base pair geometry

## Abstract

Imperfect conservation of human pre-mRNA splice sites is necessary to produce alternative isoforms. This flexibility is combined with the precision of the message reading frame. Apart from intron-termini GU_AG and the branchpoint A, the most conserved are the exon-end guanine and +5G of the intron start. Association between these guanines cannot be explained solely by base-pairing with U1 snRNA in the early spliceosome complex. U6 succeeds U1 and pairs +5G in the pre-catalytic spliceosome, while U5 binds the exon end. Current U5 snRNA reconstructions by CryoEM cannot explain the conservation of the exon-end G. Conversely, human mutation analyses show that guanines of both exon termini can suppress splicing mutations. Our U5 hypothesis explains the mechanism of splicing precision and the role of these conserved guanines in the pre-catalytic spliceosome. We propose: (1) optimal binding register for human exons and U5—the exon junction positioned at U5Loop1 C_39_|C_38_; (2) common mechanism for base-pairing of human U5 snRNA with diverse exons and bacterial *Ll.*LtrB intron with new loci in retrotransposition—guided by base pair geometry; and (3) U5 plays a significant role in specific exon recognition in the pre-catalytic spliceosome. Statistical analyses showed increased U5 Watson–Crick pairs with the 5′exon in the absence of +5G at the intron start. In 5′exon positions −3 and −5, this effect is specific to U5 snRNA rather than U1 snRNA of the early spliceosome. Increased U5 Watson–Crick pairs with 3′exon position +1 coincide with substitutions of the conserved −3C at the intron 3′end. Based on mutation and X-ray evidence, we propose that −3C pairs with U2 G_31_ juxtaposing the branchpoint and the 3′intron end. The intron-termini pair, formed in the pre-catalytic spliceosome to be ready for transition after branching, and the early involvement of the 3′intron end ensure that the 3′exon contacts U5 in the pre-catalytic complex. We suggest that splicing precision is safeguarded cooperatively by U5, U6, and U2 snRNAs that stabilize the pre-catalytic complex by Watson–Crick base pairing. In addition, our new U5 model explains the splicing effect of exon-start +1G mutations: U5 Watson–Crick pairs with exon +2C/+3G strongly promote exon inclusion. We discuss potential applications for snRNA therapeutics and gene repair by reverse splicing.

## Introduction

Human genes can generate multiple protein isoforms by alternative splicing (AS) of different sets of pre-mRNA exons, which enables another layer of regulatory control over gene function in development and adaptive processes. AS is involved in the regulation of cell fate from the earliest switch of pluripotent embryonic stem cells to specific lineages (Gabut et al., 2011; Fiszbein and Kornblihtt, 2017; Su et al., 2018) until terminal differentiation of somatic stem cells in adults (Nakka et al., 2018). AS controls the proliferation and apoptosis of specialized cells such as T cells (Corrionero et al., 2011) and response to genotoxic stress (Shkreta et al., 2011; Shkreta and Chabot, 2015; Muñoz et al., 2017).

Pre-mRNA splicing is catalyzed by the spliceosome, a multi-molecular dynamic complex, which shares a remarkably conserved ribozyme core with ancient mobile Group II introns, found in bacteria, archaea, and eukaryotic organelles. In effect, the mechanism of splicing, a 2-metal-ion-ribozyme catalysis (Steitz and Steitz, 1993; Fica et al., 2013), much predates the origin of eukaryotes and is thought to have been driving molecular evolution in the primordial RNA world (Gilbert, 1986; Koonin, 2006; Cech, 2012; Irimia and Roy, 2014).

The modern spliceosome combines the flexibility essential for the aforementioned complex gene regulation in metazoans with the routine precision of the RNA message to preserve the reading frame for the effective protein translation. The RNA components of the spliceosome, small nuclear U-RNAs (U1, U2, U6, and U5), pair short sequences in the pre-mRNA, which are imperfectly conserved to allow for alternative sites to be used. This choice of splice sites is often regulated by RNA binding proteins, RBP, and can be overruled by mutations that increase splice site complementarity to snRNAs (Hamid and Makeyev, 2017). While weak splice site conservation is clearly required to produce alternative isoforms, the exact mechanism that guarantees splicing precision in spite of these sequence variations is still unknown and is the focus of this study.

**FIGURE 1:**
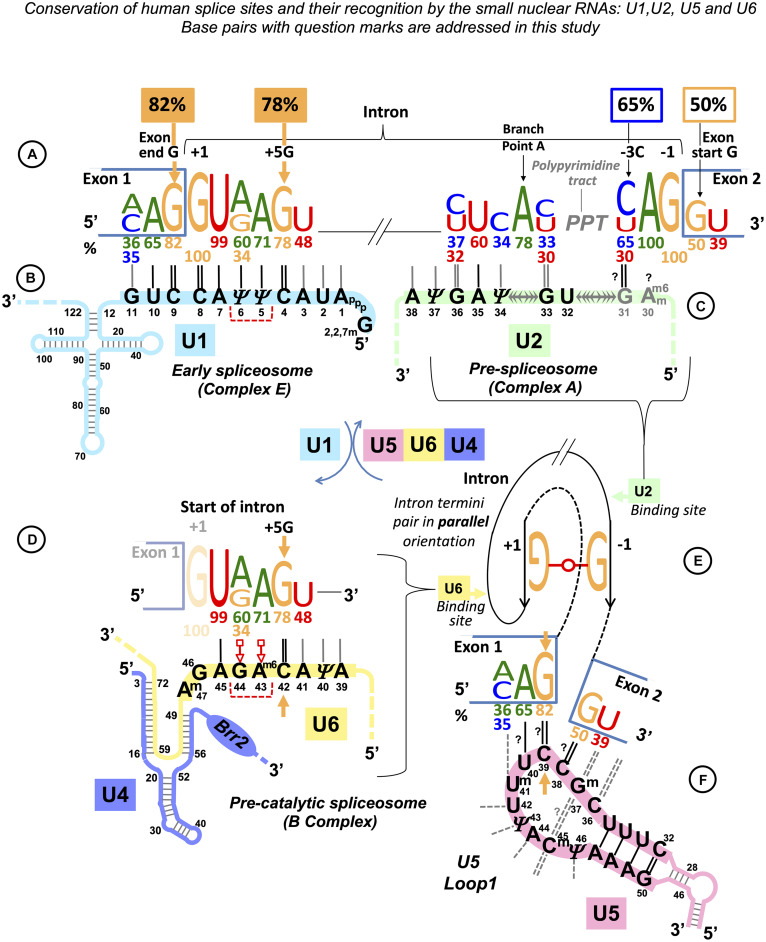
Multistage recognition of variable human splice sites by U snRNAs. **(A)** Only seven nucleotides in human introns are conserved above 75% (Sheth et al., 2006; Mercer et al., 2015). Apart from the terminal di-nucleotides and the branchpoint A, the most important are the two guanines of the exon end and at position +5 at the intron start (orange labels and arrows). **(B)** In the early spliceosome, U1 snRNA forms on average seven Watson–Crick pairs with human exon/intron boundaries (Carmel et al., 2004). **(C)** In the pre-spliceosome, U2 snRNA forms the BP helix with an adenosine bulge. (**?**) Proposed U2 G_31_ = C_–3_ pair, see DISCUSSION. **(D)** In the pre-catalytic spliceosome, U1 quits the complex and the start of the intron is passed on to U6 snRNA. +5G pairs with U6 42G (orange arrow). The conserved adenines +3, +4 form non-Watson–Crick pairs with U6 44G and 43A^m6^ (Konarska et al., 2006; Galej et al., 2016; shown here in red according to Westhof geometric classification: 10th family, Leontis et al., 2002; role explained in Figure 14 caption). The stable U6/start of the intron helix is a checkpoint for the later spliceosome activation by *Brr2* helicase (binding site on U4: blue oval). *Brr2* unwinds U6/U4 duplexes and frees U6 to configure the catalytic site of the spliceosome (Nielsen and Staley, 2012). **(E)** The strictly conserved non-canonical G⋅⋅G (2nd Westhof geometric family, see DISCUSSION, Scadden and Smith, 1995; Costa et al., 2016). **(F)** At the pre-catalytic stage, U5 snRNA comes into the complex together with U6 as part of U5⋅U4/U6 tri-snRNP (Wahl et al., 2009; Wahl and Lührmann, 2015; Scheres and Nagai, 2017; Supplementary Table S1). As U1 quits the complex, the 5′exon is passed on to U5 snRNA Loop1. For the 3′exon, see DISCUSSION. Aligned together, the exons form the splice junction consensus AG|G (proto-splice site, Sverdlov et al., 2004) pictured here paired with complementary C_38_C_39_U_40_ of the U5 Loop1. In this way, the most conserved exon-end G pairs with U5 39C (orange arrow). If so, in the pre-catalytic spliceosome, the intron-termini pair and U6 non-Watson–Crick pairs are stabilized by flanking U5/5′exon and U6/intron-start helices, each secured by one of the two important guanines of the human splice signals (orange arrows in **D,F**). Post-transcriptional base modifications of snRNAs: Ψ, pseudouridine; Superscript m, 2’O-methyl; A^m6^, N6-methyladenosine; A^m6^_*m*_, 2’O-methyl,N6-methyladenosine (modification positions as in Anokhina et al., 2013).

In human pre-mRNA introns, apart from the AG_GU di-nucleotides of the intron termini, the most conserved bases are the branchpoint adenine, the exon-end guanine (−1G), and the +5G near the start of the intron (Figure 1A; Sheth et al., 2006; Mercer et al., 2015). The relationship between these conserved guanines has been scrutinized for over 20 years (Burge and Karlin, 1997; Carmel et al., 2004; Wong et al., 2018) and linked to the initial recognition of the exon/intron boundary by U1 snRNA (Figure 1B). During the development of the GENESCAN algorithm for exon/intron gene structure prediction, Burge and Karlin (1997) statistically examined the dependencies between the nucleotides at the exon/intron boundary. The authors reported a “compensation effect”: that in the absence of the intronic +5G, the exon-end G (−1G) is almost invariant. Comparative analysis of substitutions in human and mouse orthologous 5′ splice sites also showed the same dependency between the exon-end guanine and +5G at the start of the intron (Carmel et al., 2004). A recent study (Wong et al., 2018) employed a focused massively parallel splicing assay (MPSA) to empirically examine the effects of all possible variants of the 9nt sequence NNN/GYNNNN of the exon/intron boundary on exon inclusion (percent spliced-in, PSI). This approach allowed to quantify the relationship of these conserved guanines by measuring PSI, and the authors conclude that the previously observed “seesaw linkage” pattern, whereby exon-end G (−1G) permits any nucleotide at intron position +5 and vice versa +5G allows any nucleotide at the end of the exon, is in fact “a strong positive interaction between −1G and +5G,” such that a substitution at either of these conserved positions results in over 20% reduction of PSI.

Experimentally changing a suboptimal exon-end nucleotide to G can completely suppress the effect of various splicing mutations associated with genetic disease. *IKBKAP* IVS20 (+6T → C) mutation that causes a skip of exon 20 in 99.5% of patients with familial dysautonomia (a recessive congenital neuropathy) is completely neutralized by the exon-end A → G change leading to almost 100% exon 20 inclusion (Carmel et al., 2004). *ATR* c.2101A → G mutation within exon 9 is synonymous; however, it appears to strengthen the exonic splicing silencer (ESS) and results in only a trace of the correct transcript and a very severe, but not lethal, phenotype (Seckel syndrome associated with dwarfism and microcephaly). The effect of this mutant ESS can be overruled by the change of exon-end T → G, which produced an almost exclusively normally spliced product (Scalet et al., 2017). Another example, coagulation factor 5, has an alternative intron within a 2.7-kb exon 13 that is spliced out in a small fraction of transcripts, leading to ∼1% of the Factor5-short protein isoform in plasma normally. This alternative intron is preceded by an adenine: an A → G change in this case enhances exon-end definition and leads to the predominant exclusion of this alternative intron causing a rare bleeding disorder (F5-Texas phenotype, Vincent et al., 2013).

Currently, the only mechanistic explanation of the strong dependency of exon-end G and intron +5G, as well as the ability of exon-end G to suppress splicing mutations or hyperactivate splicing, is centered on the 5′ splice site selection by base-pairing with U1 snRNA. Indeed, U1 specifically engineered to increase complementarity to 5′ss can also partially restore exon inclusion (as in Carmel et al., 2004), a discovery of Zhuang and Weiner (1986), which led to the development of snRNA therapeutics (see DISCUSSION). However, the functional 5′ splice site is not defined only by complementarity to U1 snRNA, although shifts and bulges in the U1 binding register at divergent exon–intron boundaries have been proposed to fix the problem of poor conservation (Roca et al., 2012, 2013; Tan et al., 2016). In the early spliceosomal complex (complex E), U1 snRNA binds multiple alternative or cryptic sites, and the commitment to splicing depends on both the affinity to the target and relative positions of the U1 and U2 binding sites (Eperon et al., 1993, 2000). Multiple U1 snRNAs can bind initially, and the surplus of U1 is removed after U2 snRNP interacts with U1 snRNP during the transition to complex A (Hodson et al., 2012; see Supplementary Table S1 for successive spliceosomal complexes). It is also long known that the 5′ splice site is not defined relative to the base-pairing with U1 snRNA. Indeed, U1 snRNAs engineered to base-pair in the vicinity rather than exactly at the exon/intron boundary can rescue the inclusion of exons with splicing mutations in cell culture and in mouse models (Fernandez Alanis et al., 2012; Rogalska et al., 2016; reviewed in Singh and Singh, 2019). This variability of U1 binding cannot support the precise definition of the 5′ splice site, which means that the binding register of U6 snRNA is the final determinant of the intron 5′ boundary—an explanation put forward by Hwang and Cohen (1996). In addition, U1-independent splicing was also discovered in HeLa nuclear extracts: Crispino and Sharp (1995) show that complementarity to U6 snRNA enhances splicing if U1 is depleted. The authors confirm experimentally that U6 can form Watson–Crick pairs with the intron until position +9. More recent studies (reviewed in Fukumura and Inoue, 2009) show that at least a fraction of human introns normally rely on U1-independent splicing. Moreover, engineering increased complementarity to U1 at the exon/intron boundary disrupts the normal splicing pattern, overruling exon exclusion prompted by Fox-1 RBP (Fukumura et al., 2009).

A recent evolutionary insight provided by a monocellular red alga *Cyanidioschyzon merolae* that lacks U1 snRNA and all its protein co-factors (U1 snRNP) shows that U1 is altogether dispensable for pre-mRNA splicing (Matsuzaki et al., 2004; Stark et al., 2015). Single intron genes of this exceptional eukaryote do not require alternative processing. This indicates that U1 is needed to facilitate flexible splice site choices, rather than splicing precision, and confirms that U6 controls the 5′ intron boundary definition. Moreover, the formation of the U6 helix with the start of the intron (the so-called U6 ACAGAGA interaction) is considered to trigger subsequent activation of the human pre-catalytic spliceosome (complex B, Charenton et al., 2019). The difficulty is that one in five human introns lacks the essential +5G that pairs with U6 42C to secure this interaction (Figure 1D). In *Saccharomyces cerevisiae*, the first model organism of the spliceosome studies, U_+__4_G_+__5_U_+__6_ are absolutely conserved and all form Watson–Crick pairs with U6 snRNA. Upstream position +3 forms a non-Watson–Crick pair essential for the correct repositioning of the lariat intermediate after branching (Konarska et al., 2006). In humans, the conservation of +3A is less reliable, so adenine is repeated in position +4, which ensures the presence of at least one of these key purine pairs. This, however, takes out a Watson–Crick pair, and given that the conservation of +6U is below 50%, preservation of this checkpoint U6 helix is altogether elusive, suggesting the need for other specific interactions in the pre-catalytic spliceosome.

The 3′ intron-end motifs(Figure 1A, right) are initially recognized by proteins: SF1 binds the branchpoint (BP), and the large subunit of the U2 snRNP auxiliary factor protein, U2AF^65^, tethers the polypyrimidine tract (PPT), while the small subunit U2AF^35^ binds the intron-end AG. Only then U2 snRNA can pair with its target sequence around the BP (Figure 1C, Complex A). This is quite unlike the usual way when RNA guides a protein enzyme to the RNA or DNA target. Mutations at the 3′ intron end can also be suppressed by increasing complementarity of U2 snRNA to the sequence around the branchpoint (Zhuang and Weiner, 1989). Polypyrimidine tract between the branchpoint and the 3′ intron end is highly variable in length and nucleotide composition. Crystal structures of U2AF^65^ bound to poly-U indicate a sharp kink of the RNA strand (Sickmier et al., 2006) and, moreover, conversion of uridines to pseudouridines, which confers rigidity to the RNA backbone, blocks U2AF^65^ binding (Chen et al., 2010). Oddly, no RNA partner has been identified so far for the conserved position −3 at the end of the intron, although mutations in this position impair or block splicing completely. The effect of −3C → G change was explored in *Fas/CD95* intron 5: while U2AF^65^ binding was not affected, this mutation blocked U2 snRNA binding (Corrionero et al., 2011; see DISCUSSION).

Next, adenine at intron position −2 is absolutely conserved (Figure 1A). According to the Cryo-EM studies, −2A interacts with the BP A (two H-bonds between Hoogsteen edges of adenines, glycosidic bonds in *trans* orientation), which helps to position the 3′ exon for the ligation (reviewed in Wilkinson et al., 2020). Intron termini guanines form a pair that conserves local parallel strand orientation as in Group II introns and in eukaryotes can be substituted exclusively by A_C intron ends (see DISCUSSION for the exact pair configuration). As only seven nucleotides of the human splice sites are conserved above 75%, the fact that half of all human exons start with a guanine is significant. Fu et al. (2011) reported that G → T mutations at the start of *GH1* exon 3, *FECH* exon 9, and *EYA1* exon 10 cause exon skipping, while G → T change at the start of *LPL* exon 5 and G → A change at the start of *HEXA* exon 13 do not affect exon inclusion. The authors explain it by the shorter PPT stretch that precedes mutations affecting splicing. However, partial exon inclusion for the neutral substitutions of the exon-start G persists even if the PPT stretch is reduced to 2–5 nucleotides. Remarkably, both these neutral changes are followed by cytosines in exon position +2. Fu et al. (2011) continued to quantify variable splicing effect of further nine exon +1G mutations in different human genes. Here, we re-examine their data and link the exon positions +2 and +3 with the inclusion efficiency.

As in the case of the exon-end guanine, experimentally changing a suboptimal exon-start nucleotide to guanine can suppress *ATR* c.2101A → G mutation of the exon 9 ESS and restores exon inclusion to the wt level (Scalet et al., 2017). Unlike for the exon-end guanine, the mechanistic basis for the splicing re-activation by the exon-start G (+1G) or the variable effect of +1G mutations on splicing cannot be explained by the initial U1 selection, which points at a later stage interactions of exon sequences at splice junctions with U5 snRNA Loop1. Base-pairing of exons with U5 proved to be the most challenging of all pre-mRNA interactions with snRNAs, possibly due to the fact that as opposed to the U6 binding site at the start of the intron and the sequence around the BP, the exon sequences at splice junctions are less conserved in *S. cerevisiae* than they are in humans. So, even the binding register of the exons with U5 Loop1 presented a problem. While for the intron interactions with U6 and U2, easy alignment facilitated compensatory double mutation analyses (Zhuang and Weiner, 1989; Crispino and Sharp, 1995; Hwang and Cohen, 1996), mutation analysis of U5 Loop1 was jumbled up by the absence of the interaction model, although mutant U5 variants promoted the activation of new splice sites (Cortes et al., 1993). Crosslinking experiments of the 1990s involved 4-thiouridine (4sU) substitutions of the conserved guanines of the exon termini and could not show the wild-type base pairing with U5 Loop1 (Sontheimer and Steitz, 1993; Newman et al., 1995; schematics in Supplementary Table S2). Both 5′ exon and 3′ exon 4sUs crosslinked to two positions of the loop: 5′ to U_40_ and U_41_ and 3′ to C_39_ and U_40_. Since the start of CryoEM structural studies of the *S.c.* and human spliceosomes, the pains to place the exons relative to U5 Loop1 produced no less than five different binding registers (Galej et al., 2016; Rauhut et al., 2016; Wan et al., 2016; Yan et al., 2016; Bertram et al., 2017a, b; Zhang et al., 2017; schematics in Supplementary Table S2). Initially, Rauhut et al. (2016) modeled 11 nt U5 Loop1 with the exon-end G unpaired and the U_+__2_ interactions as in the crosslinking experiments. Galej et al. (2016) presented 7 nt U5 Loop1 with the exon-end G also unpaired and A_+__2_A_+__3_A_+__4_ forming Watson–Crick pairs with U5 U_97_U_98_U_99_ (human U^m^_41_U_42_Ψ_43_). Wan et al. (2016) were first to present exon-end G paired to U5 C_95_ (human C_39_) and A_+__2_A_+__3_ paired with U_97_U_98_ (human U^m^_41_U_42_) of the 7 nt U5 Loop1. Yan et al. (2016) then placed G_+__1_A_+__2_A_+__3_ paired with U_97_U_98_U_99_ (human U^m^_41_U_42_Ψ_43_). All Cryo-EM reconstructions for the human spliceosome use the MINX pre-mRNA substrate, which contains a small composite adenovirus intron (Supplementary Tables S2, S3), yet Bertram et al. (2017a) place the 5′ exon end G_+__1_C_+__2_A_+__3_ paired with U^m^_41_U_42_Ψ_43_ and Bertram et al. (2017b) place it with U_40_U^m^_41_U_42_, with U5 Loop1 open to 11 nt. The other structures by Zhang et al. (2017) and Zhan et al. (2018a, b) also place this 5′ exon end G_+__1_C_+__2_A_+__3_A_+__4_ paired with U5 U_40_U^m^_41_U_42_Ψ_43_ but with the small 7 nt version of U5 Loop1. The binding register for the 5′ exon with the exon-end G paired to U5 U_40_ of the 7nt U5 Loop1 currently prevails (see DISCUSSION), as it is featured in the most recent structures with the best resolution (Zhang et al., 2019). On the contrary, base-pairing for the 3′ exon is still unresolved. The root of the problem is the timing of this interaction and the mechanistic challenges of bringing the 3′ exon into the catalytic core with the variable PPT stretch between the branchpoint and the 3′ss. The timing is not a problem for the 5′exon, as when U1 quits the complex at the pre-catalytic stage (complex B), the start of the intron is passed on to U6 snRNA and the end of the 5′ exon binds U5 snRNA Loop1. We consider the key role of the intron termini pair in the mechanism of splicing catalysis to adjust the timing for the 3′ exon interaction with U5 (see DISCUSSION).

These varying Cryo-EM reconstructions of U5 base pairs with the 5′ exon inclusive of the latest version and the lack of clear base-pairing for the 3′ exon with the remaining part of the loop do not seem to connect to genetic studies reviewed above. The latest binding register for the 5′ exon does not include a G = C pair for the 82% conserved exon-end guanine, and the 7 nt loop is so small that it does not allow much base-pairing for two exons (see DISCUSSION). The structures suggest that U5 Loop1 plays little role in the recognition of the exon sequences at splice junctions and thus cannot contribute to splicing fidelity. However, poor conservation of every nucleotide in human splice site sequences must be accounted for with a specific interaction, which all combined have to ensure splicing precision. We ask a question if this can be managed by U1, U2, and U6 without a substantial contribution from U5.

We start with a different approach to U5 modeling and first compare splicing with the retrotransposition of a mobile bacterial Group IIA intron. Small nuclear RNAs U2, U6, and U5, which assemble on the pre-mRNA in the spliceosome core, are homologous to Group II RNA domains (Zimmerly and Semper, 2015; Galej et al., 2018; detailed in DISCUSSION). In particular, the U5 Loop1 homolog, Id3 Loop of Domain I, controls the specificity of the Group IIA intron splicing by Watson–Crick base-pairing with the exons (Lambowitz and Belfort, 2015; Dong et al., 2020). In retrotransposition, mobile Group IIA introns invade new loci by splicing in reverse into genomic targets “similar” to their exons (Ichiyanagi et al., 2002; Coros et al., 2005; Novikova et al., 2014; Lambowitz and Belfort, 2015). The “similarity” of retrotransposition sites is so far not clearly defined, but in effect, the unique DId3 Loop pairs variable target sites just like the universal U5 snRNA Loop1 fits all the diverse exon junctions in the human genome.

Here, we compare the alignments of human splice junctions with U5 Loop1 to the alignments of bacterial retrotransposition sites with the Group IIA DId3 loop. We propose a common mechanism of base-pairing for human U5 snRNA with diverse exons and the bacterial *Ll.*LtrB intron with new loci in retrotransposition: recognition guided by base pair geometry. Statistical analyses of U5 interactions with human exons lend support to our alignment model with the optimal binding register for the splice junction of exons positioned at U5 Loop1 C_39_|C_38_. We find that U5 Watson–Crick pairs with the exons show a clear pattern of compensation for substitutions of the conserved nucleotides in human introns, indicating a collective mechanism whereby U5, U6, and U2 recognize their variable binding sites. We suggest that snRNAs in the pre-catalytic spliceosome together ensure fidelity before the committed ribozyme core is configured. In addition, we clearly explain the effect of human mutations on splicing (Fu et al., 2011) by base-pairing of the 3′ exon with U5 Loop1.

Our findings result in a new model for U5 snRNA interactions with the exons that is central in the precision mechanism of pre-mRNA splicing. We propose verification experiments and future therapeutic applications.

## Results

### Modeling U5 Loop1 Base Pairing With Human Exons on Group IIA Intron Interactions With Retrotransposition Sites

We considered that the types of pairs acceptable in the interactions of Group IIA introns with variable target sites might provide a clue to the way human exons pair with U5 snRNA. A pilot investigation of a small number of published sequences of *Ll.*LtrB retrotransposition sites and splice junctions of just one human gene, albeit a giant *dystrophin*, was performed. Detailed examination and sequence alignments of these small datasets provided a pilot hypothesis and guided the design of a series of statistical tests on a large number of human splice junctions and intron sequences.

#### Base Pair Types in the Interactions of *Ll.*LtrB With Retrotransposition Sites

**FIGURE 2:**
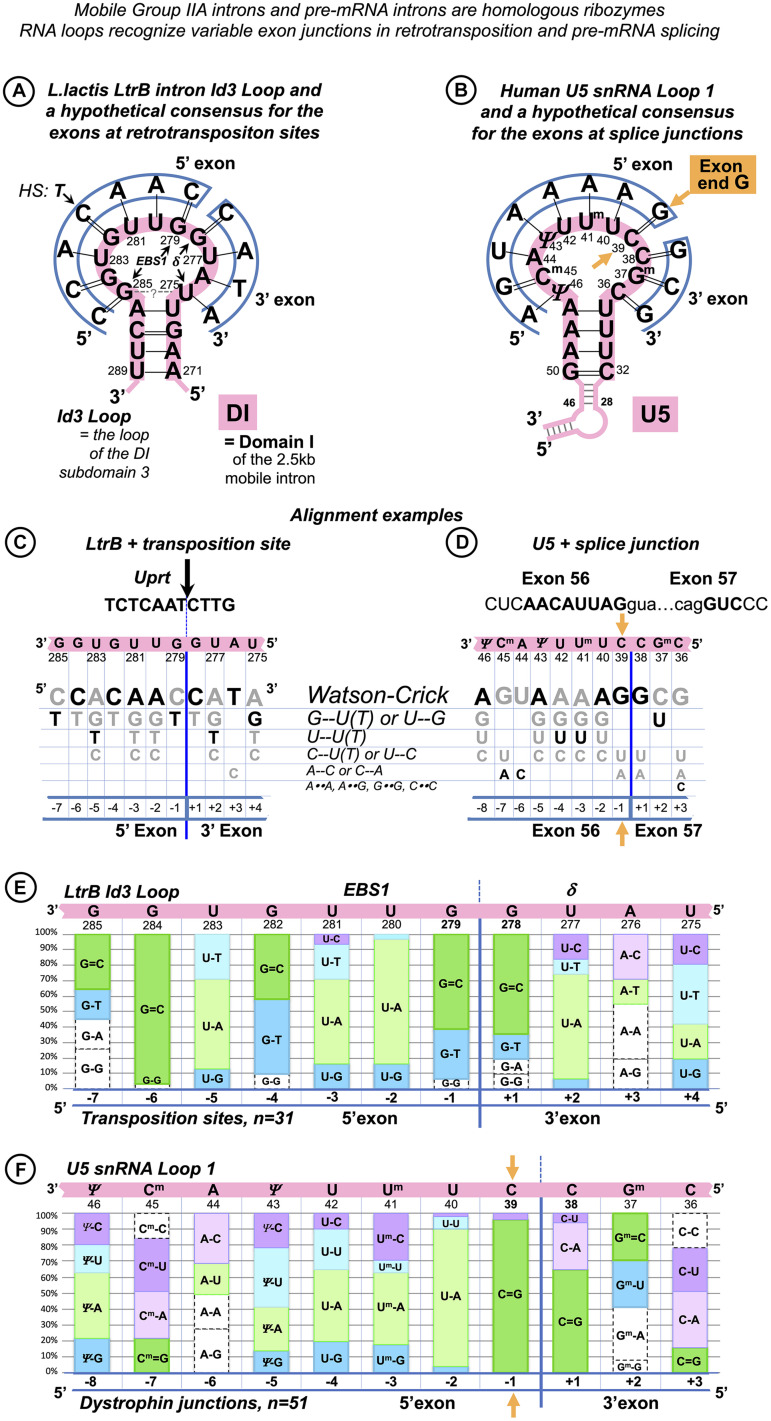
Base pair composition for the target recognition by mobile *Ll.*LtrB intron helps to identify the binding register for the human exons and U5 snRNA. **(A)** The 11nt Id3 Loop in Domain I (DI) is the element of the *Ll.*LtrB intron responsible for the specific recognition of the exons. C = G pairs with guanines in Id3 positions 278 and 279 coordinate the splice junction. The Id3 loop of the excised intron can pair with genomic targets similar to the homing site and guide retrotransposition. We derived a hypothetical consensus for retrotransposition sites based on the complementarity to the Id3 Loop. The homing site (*HS*) in the *ltrB* gene differs from this consensus in the 5′ exon position −4 (thymidine). ***EBS1***: Seven nucleotides of the Id3 loop (positions 279–285) pair with the end of the 5′ exon. *δ:* The remaining four nucleotides (positions 275–278) form a helix with the start of the 3′ exon. **(B)** Assuming that, like in Group IIA introns, Watson–Crick pairs are preferred, we derived a hypothetical consensus complementary to U5 Loop1. The actual human splice junction consensus AG|G (Figure 1F) appears incorporated into this hypothetical sequence and G = C pairs with cytosines in U5 positions 38 and 39 coordinate the splice junction (orange arrow: U5 39C pairs the conserved exon-end G). **(C)** We derived a grid for manual alignment of the retrotransposition sites with the Id3 loop that listed Watson–Crick and frequent mismatched pairs. In this way, we recorded base pairs involved in the recognition by the LtrB intron of 31 targets in the *L. lactis* genome (Ichiyanagi et al., 2002; one example is shown here). **(D)** Assuming that U5 snRNA Loop1 has the same base pair preferences as the *Ll.*LtrB Id3 Loop and that the 5′ exon helix is longer than the 3′ exon helix, we superimposed each of the 78 dystrophin gene splice junctions on the grid in the five possible binding registers (as in the example here). Alignments with most Watson–Crick pairs were chosen as most likely with 65% of dystrophin exon junctions unambiguously aligned to U5 C_38_| C_39_ (as in **B,D**) and further 30% also fit this and one or two alternative binding registers. **(E)** Summary for the *Ll.*LtrB Id3 Loop of the total *n* = 341 bp with 31 retrotransposition sites (Ichiyanagi et al., 2002). **(F)** Summary for the human U5 snRNA Loop 1 of the total *n* = 561 bp with only 51 dystrophin splice junctions that unambiguously aligned with U5 C_38_|C_39_. Base modifications as in Figure 1 caption.

We chose *Ll.*LtrB, a well-studied mobile Group IIA intron from *Lactococcus lactis* (Ichiyanagi et al., 2002; Coros et al., 2005; Novikova et al., 2014; Dong et al., 2020; Ll.I1 in the Zimmerly Lab Group II intron database^1^, Candales et al., 2012). This 2.5-kb intron of the *ltrB* gene (encoding a relaxase found in the conjugative elements) folds into a typical structure of the Group IIA ribozyme: RNA domains DI to DVI (Dong et al., 2020). The intron catalyzes its own splicing, and the excised intron lariat can undergo specific reverse splicing to insert into the “homing” site of the intron-less allele (retrohoming) or invade a new genomic locus choosing a “similar” target sequence (retrotransposition). *Ll*.LtrB Id3 Loop is uracil-rich like U5 snRNA Loop1 (5 out of 11 nucleotides are uracils; Figures 2A,B). Seven nucleotides of the loop bind the end of the 5′exon, and four nucleotides bind the start of the 3′ exon (Figure 2A). This is a typical pattern of exon binding by the intron ribozyme of the subclass IIA (Supplementary Figure S1).

We examined the published sequences (Ichiyanagi et al., 2002) of retrotransposition sites (*n* = 31) for the base pair content of their interactions with *Ll*.LtrB Id3 Loop (Figure 2C). Apart from canonical Watson–Crick pairs (55%, 186 of 341), G-T/U-G (17%) and U-T pairs (9%) appeared to be the most frequent in these interactions (Figure 2E).

#### Binding Register and Base Pair Types in the Interactions of U5 snRNA With the Dystrophin Exons

We examined possible U5 binding registers individually for 78 splice junctions of the human dystrophin full-length skeletal muscle mRNA. We assumed that (1) the end of the 5′ exon forms a longer helix with the recognition loop than the start of the 3′exon as in Group II introns; (2) the preferred pairs are Watson–Crick; (3) the types of frequent mismatched pairs are common for these RNA loops: we used the same grid that lists all possible base pair types in order of frequency observed in *Ll*.LtrB retrotransposition [first Watson–Crick followed by mismatches G-U(T)/U-G, U-U(T), C-U/U-C] for the human U5 snRNA Loop1 (Figures 2C,D) to align manually 10 nt at the end of each exon joined to 5 nt of the start of the next exon. The sequence was superimposed on the grid for all five binding registers that allow for a longer 5′exon helix, and alignments with the most Watson–Crick pairs were chosen as most likely. 65% of dystrophin exon junctions unambiguously aligned to U5 positions C_38_|C_39_ (Figure 2B); a further 30% also fit this and equally one or two alternative binding registers. Therefore, a total of 95% of dystrophin mRNA splice junctions match the same U5 position, indicating that U5 C_38_|C_39_ is the optimal fixed binding position for the exon junction. This position is subsequently referred to as “the proposed binding register” and used for the statistical analysis below.

As 5% of dystrophin exon junctions appear to match alternative positions better than U5 C_38_|C_39_, a possibility of an occasional shift of the U5 binding register cannot be outruled. A single relevant piece of evidence concerns the reverse splicing of a Group II intron into a mutant homing site (HS, exon junction in the intronless allele): Su et al. (2001) reported a shift in the binding register by one nucleotide that secured a G = C pair.

While we assume that possible shifts in the U5 binding register are rare, the incorporation of non-canonical mismatched pairs alongside canonical Watson–Crick is inevitable in exon recognition helices. Accordingly, the base pair composition for dystrophin junctions that aligned unambiguously to U5 C_38_| C_39_ (*n* = 51) was as follows: 45% Watson–Crick (252 of 561), 14% C-U/U-C, 11% A-C/C-A, 10% G-U/U-G, and 9% U-U (Figure 2F).

#### Common Mismatched Pairs Are Interchangeable for Watson–Crick Pairs

What makes these mismatched pairs acceptable in the interactions of *Ll*.LtrB with diverse genomic targets, and the proposed interactions of U5 with the multitude of exon sequences? It appears that G-U, A-C, C-U, and U-U pairs have an important quality in common: they can assume Watson–Crick-like geometry in different cellular molecular systems (Bebenek et al., 2011; Wang et al., 2011; Rozov et al., 2015; Rypniewski et al., 2016). In effect, a single repositioning of a proton (prototropic tautomerization) or the addition of a proton (protonation) for one of the bases in these pairs can produce configurations resembling the shape of the canonical pairs (see DISCUSSION). Further, in this paper, these pairs are termed “isosteric” as opposed to A-G, G-G, A-A, and C-C pairs that are always distinct from Watson–Crick geometry and thus disrupt the architecture of the recognition helices (The theoretically possible Watson–Crick-like C–C configuration requires both imino tautomerization and protonation—a pair not featured in any structures to date). For convenience, isosteric pairs are subsequently represented by a double dash “G–U,” non-isosteric with a double dot “G⋅⋅A,” canonical Watson–Crick with a single dash for A-U, an equal sign for the triple-H-bonded G = C, and non-isosteric “wobble” pairs with a single dot “G⋅U.”

Figure 2E is the essential evidence of the co-variation of Watson–Crick and isosteric mismatched pairs. During self-splicing or retro-homing (reverse self-splicing), the *Ll.*LtrB Id3 Loop forms Watson–Crick pairs at every position of the splice junction, except position −4 of the 5′ exon. Assuming that in retrotransposition the shape of pairs is the key to target recognition, G--U, A--C, C--U, and U--U are acceptable only in their isosteric configuration. Remarkably, position −4 demonstrates a reciprocal example: during self-splicing or retro-homing, the 5′ exon of the “home” gene forms a U_–4_--G_282_ pair with the Id3 loop of the LtrB intron. In retrotransposition, whereas 48% of integration sites conserve U_–4_--G_282_, 42% change to canonical Watson–Crick C_–4_ = G_282_. Isosteric U--G with either base in enol configuration is a high-frequency pair (previous NMR data—Kimsey et al., 2015—discussed below) and as opposed to differently shaped wobble U⋅G explains the occurrences of U--G/G--U pairs in various positions in the interactions of many other Group II introns with the exons of their home genes (Supplementary Figure S1).

Figure 2F presents a homologous co-variation of Watson–Crick and isosteric pairs for the U5 Loop1 with human dystrophin gene exons. In particular, at position −3 of the 5′exon, the proposed binding register shows co-variation of C--U and A-U pairs. In the early spliceosomal complex that precedes U5 binding, exon positions −1 to −3 interact with U1 snRNA C_9_U_10_G_11_ and select for exon-end C_–3_A_–2_G_–1_ (Figure 1B). In fact, in the dystrophin gene, the ratio of C/A in position −3 is ¾ (in the whole human genome, it is near 1:1, Figure 1A). Although Cryo-EM studies pictured 5′exon paired with U5 in different registers, position −3 was always aligned with one of the uracils (Supplementary Table S2), effectively admitting the A-U/C--U co-variation. In our model, exon position −3 pairs with U5 41-2’O-methyl-uracyl with co-variation of the A_–3_-U^m^_41_ and C_–3_--U^m^_41_ (Supplementary Figure S3 and Figure 2F).

In summary, we suggest that base pair geometry is the key to the recognition of exon junctions by the spliceosome and retrotransposition sites by the LtrB Group IIA intron. Watson–Crick pairs are selected for, isosteric pairs (G--U, A--C, U--U, and U--C) are accepted, while pairs that perturb the helix architecture are kept out of these interactions. An important quality of uracil is that it can form isosteric pairs with any other base (see DISCUSSION), so uracil-rich RNA loops like spliceosomal U5 Loop1 and *Ll.*LtrB Id3 Loop are useful for semi-specific sequence recognition, relying on isosteric mismatched pairs supported by Watson–Crick pairs to preserve the shape of the RNA helix. This mechanism explains how the universal U5 Loop1 can bind the multitude of diverse human exon junctions and equally explains *Ll.*LtrB intron mobility by reverse splicing into new genomic targets, with sequence “similarity” defined by acceptable base pair geometry.

#### U5 Watson–Crick Pairs With the Exons in the Proposed Binding Register Compensate for Substitutions of the Conserved +5G in the Dystrophin Introns

**FIGURE 3:**
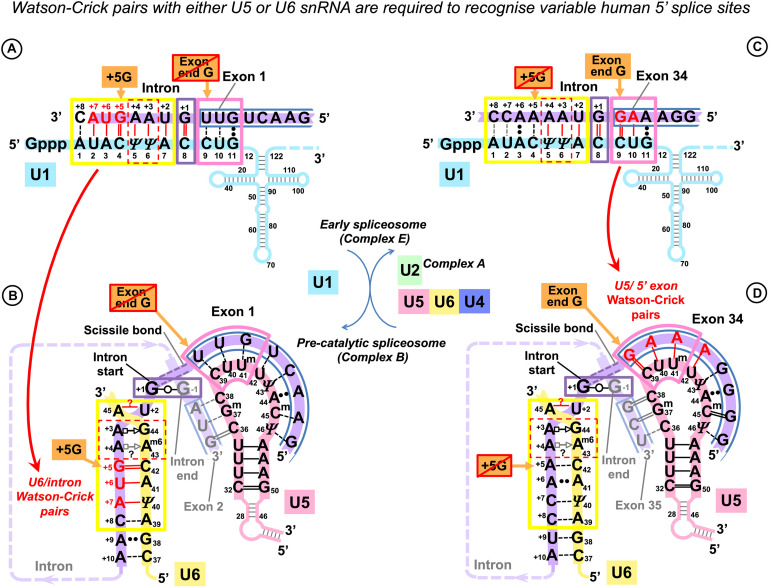
U5 and U6 recognize variable human exon/intron boundaries by Watson–Crick base pairing at the pre-catalytic stage and cooperatively ensure splicing fidelity. **(A,C)** In the early spliceosome (complex E), U1 snRNA forms, on average, seven (minimum five) Watson–Crick pairs with the exon/intron boundary (Ketterling et al., 1999; Carmel et al., 2004). U1 can bind multiple alternative and cryptic targets (Eperon et al., 1993, 2000) and is known to initiate correct splicing when bound in the vicinity rather than at the actual exon/intron boundary (Fernandez Alanis et al., 2012; Singh and Singh, 2019), presumably leaving the fidelity check for the next stage. **(B,D)** In the pre-catalytic spliceosome (complex B), U1 is replaced by U5 snRNA at the exon end (pink boxes). U6 snRNA replaces U1 at the intron start (yellow boxes). The well-conserved adenines at intron positions +3 and +4 are enclosed in a red dashed box. Initially, these adenines pair with U1 pseudouridines 5 and 6, and then in the pre-catalytic complex, they form non-canonical pairs with U6 A^m6^_43_G_44_ (Figure 1D). Intron termini pair (purple box): see Figure 1E and DISCUSSION. **(C)** Lack of exon complementarity to U5 is compensated by a strong intron interaction with U6 (as an example: human dystrophin intron 1). **(D)** *Vice versa*, lack of intron complementarity to U6 is compensated by U5 Watson–Crick pairs with the 5′exon: (as an example: dystrophin intron 34). Base modifications as in Figure 1.

Some dystrophin gene exons cannot form any Watson–Crick pairs with U5 snRNA in the proposed binding register. We noticed that, in such cases, there is always a perfectly conserved U6 binding site G_+__5_U_+__6_A_+__7_(U_+__8_) at the start of the intron that forms Watson–Crick pairs with U6 positions (39)40 to 42 (Figures 3A,B). Conversely, among the 78 dystrophin gene introns, 18 (23%) lack the conserved +5G, and all of these introns are preceded by exons that form multiple Watson–Crick pairs with U5 snRNA in the proposed binding register (Figures 3C,D).

Effectively, in the human dystrophin gene, U5 and U6 snRNAs mutually compensate for the loss of complementarity at their binding sites, stabilizing the pre-catalytic complex with Watson–Crick base pairing. These observations in this small dataset hinted that it is the collective effect of U5 and U6 that ensures splicing precision in the context of variable splice signals of human genes.

### Statistical Testing of the New Model of the Interactions of U5 snRNA With Human Splice Junctions

The pilot hypothesis indicates a distinctive binding register for the exons and U5 snRNA and places the splice junction so that the end of the 5′ exon is paired with U5 39C and the start of the 3′ exon binds U5 38C. This binding register appears to be linked to the mechanism of coordinated and mutually supportive splice signal recognition by U5 and U6 snRNAs.

In order to investigate if what is true for the dystrophin pre-mRNA is a general rule, we planned statistical tests that compare base pair distributions in the interactions of U5 and U6 snRNAs, placing U5 interactions according to the new model.

We also paid special attention in distinguishing the roles of U5 and U1 snRNA, which binds the last three positions of the exons during the initial selection of exon/intron boundaries. The focus of the series of statistical tests described below is to validate the functional importance of the new model of the U5 interactions with the exons.

#### Dataset of Base Pairs in the Interactions Between Human Pre-mRNAs and snRNAs

Rather than scoring nucleotide distributions in exons and introns, we generated datasets of base pairs of their interactions with snRNAs. We opted to select transcripts of well-studied human genes, rather than a massive approach, for the purpose of excluding inferred splicing events. These selected genes are responsible for a wide range of functions (Supplementary Figure S12), and their exon/intron structure is representative of human protein-coding genes (Supplementary Figure S13). In order to enable the analysis of the role of variation at specific positions in the splice junction, it is necessary to have a sufficiently large dataset of base pairs for U5 and U6 snRNAs at their pre-mRNA binding sites. Aiming to create a dataset of approximately 2,000 introns, we paired *in silico* the splice sites (ss) from 132 human genes (Supplementary List S1) with the snRNAs and computed for each ss position the frequency of base pairs grouped into three categories depending on their geometric properties. These are Watson–Crick pairs (G = C/C = G and A-U/U-A), isosteric pairs as defined above (G--U/U--G, U--U, C--U/U--C, and A--C/C--A), and non-isosteric pairs (A⋅⋅G/G⋅⋅A, A⋅⋅A, G⋅⋅G, and C⋅⋅C).

The 132 selected genes contain 2,007 introns and their respective exon junctions (Supplementary List S1). Four minor introns with U6atac binding site motif (processed by the alternative spliceosome; see DISCUSSION) were excluded from subsequent analysis (Supplementary List S2). Thirteen atypical major introns with substitutions of the usual +2U (+2C in 12 introns and +2A in 1 intron, Supplementary List S3) were also excluded from the analysis of the 5′ splice site interactions with U5 and U6, as the observed multiple Watson–Crick pairs on both sides of the exon/intron boundary are likely to stabilize the unusual U6 A_45_--C_+__2_(A_+__2_) pair, rather than indicate any correspondence between the end of exon and start of intron positions +5 to +8. The final dataset consisted of 1,990 major spliceosome GU_AG introns and their respective exon junctions.

#### The Effects of Intron +5G and Exon −1G Substitutions at the 5′ Splice Site

The analyses below show that U5 snRNA contributes to the precise definition of the 5′ splice site in the pre-catalytic complex forming more Watson–Crick pairs with the 5′ exon positions −5, −3, −2, and −1 to compensate for the loss of +5G, the most conserved residue of the U6 binding site at the start of the intron. Reciprocally, U6 snRNA forms more Watson–Crick pairs with the intron positions +5 to +8 to compensate for substitutions of the most conserved exon-end guanine (−1G) of the U5/splice junction interaction.

##### U5 Watson–Crick Pairs With the 5′ Exon Compensate for Substitutions of the Conserved +5G in the Following Intron

For the first experiment, we sorted exon junctions into two groups; the first one contained introns that conserved +5G and the second: introns with substitutions of +5G (+5Gsub).

**FIGURE 4:**
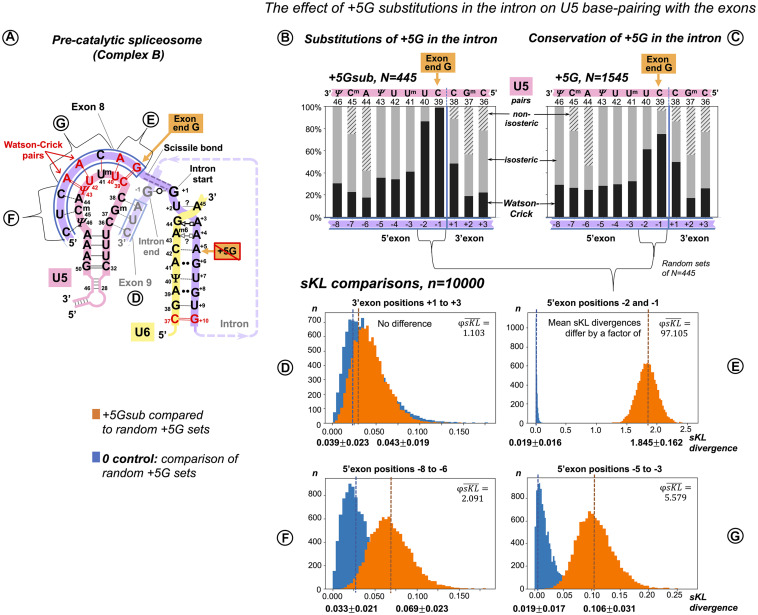
Additional U5 Watson–Crick pairs with the 5′exon compensate for substitutions of the conserved +5G at the start of the intron. **(A)** Schematic of the interactions with U5 and U6 snRNAs (using human dystrophin splice junction of exons 8 and 9 and the start of intron 8 as an example). The 11 base pairs of the U5 interaction with the exons are here subdivided into four groups that correspond to sKL distributions **(D–G). (B)** Base pair frequencies for 445 human splice junctions related to introns lacking +5G (+5Gsub). **(C)** Base pair composition for 1,545 splice junctions of introns with conserved +5G. **(D–G)** Distributions of symmetrized Kullback–Leibler divergences (sKL) for each positional group between the +5Gsub and 10,000 random non-redundant +5G sets of *n* = 445 (orange histograms) and control distributions of random non-overlapping and non-redundant sets of the same size from within the +5G dataset (blue histograms).

Plotting the proportion of Watson–Crick, isosteric and non-isosteric pairs as a function of position, we observed an increase in Watson–Crick pairs between the 5′ exon and U5 snRNA in the absence of the conserved U6 C_41_ = G_+__5_ pair at the start of the intron (Figures 4A–C). However, there is no change in the base pair composition of the interaction of the 3′ exon with U5 between +5G and +5Gsub groups.

We compared the distribution of U5 base pair types by computing Kullback–Leibler (KL) divergence, a statistic used previously to compare the distributions of nucleotides at splice sites (Sheth et al., 2006). Originating in information theory (Kullback and Leibler, 1951), KL divergence is a measure of relative Shannon entropy (variation) between two probability distributions, a cumulative statistic that sums up *all* the changes in the two distributions as logs of relative probabilities. The original KL divergence is not symmetric: KL(P,Q) ≠ KL(Q,P). The symmetrized KL (sKL) divergence is a sum of KL divergences of distribution P from Q and Q from P (“there and back again”). To assess the effect of the base pair position, we divided the splice junction into subsites (Figure 4A) and evaluated the extent of changes by sKL at each of these subsites (Figures 4D–G).

Our two datasets are naturally of unequal size: introns with conserved +5G, *N* = 1,545 and introns with substitutions +5Gsub, *N* = 445. sKL divergence is a relative measure, so it is useful to have a control with “no difference.” Here, as control, we used pairs of random non-overlapping and non-redundant sets of *N* = 445 drawn from the +5G dataset. One +5G set of each such pair was also compared to the +5Gsub dataset. Ten thousand iterations of these procedures returned distributions of sKL divergences within +5G sets (control) and between the +5Gsub dataset and +5G sets. If these distributions superimpose, there is essentially no difference between the two cases as exemplified by Figure 4D – the 3′exon. The sKL distributions are well separated in Figure 4E (mean sKL divergences differ by a factor of $\varphi \bar{sKL}=97.105$), which shows that the base pair composition is very different at exon positions −2 and −1. This is easily seen in the increased proportion of Watson–Crick pairs with U5 snRNA in these positions in the +5Gsub dataset (Figure 4B vs. 4C). An ∼17-fold smaller effect of the +5G substitutions is evident at 5′ exon positions −5 to −3 (Figure 4G, $\varphi \bar{sKL}=5.579$). On the other hand, in positions −8 to −6, sKL distributions almost superimpose (Figure 4F, $\varphi \bar{sKL}=2.091$), indicating no or little effect of the conserved intron position +5.

However, our comparisons of sKL divergence distributions show the cumulative change of all the three base pair types at two or three positions of the splice junction at a time. The smaller change at 5′exon positions −5 to −3 is not convincing to distinguish between the U5 interaction in the pre-catalytic spliceosome and the U1 interaction in the early spliceosome. To overcome these limitations, we measured the variation of distinct U5 base pairs at individual positions in the splice junction correlated with substitutions of the conserved guanine at the intron position +5.

##### Functional Importance of Distinct U5 Base Pairs at Specific Positions

**FIGURE 5:**
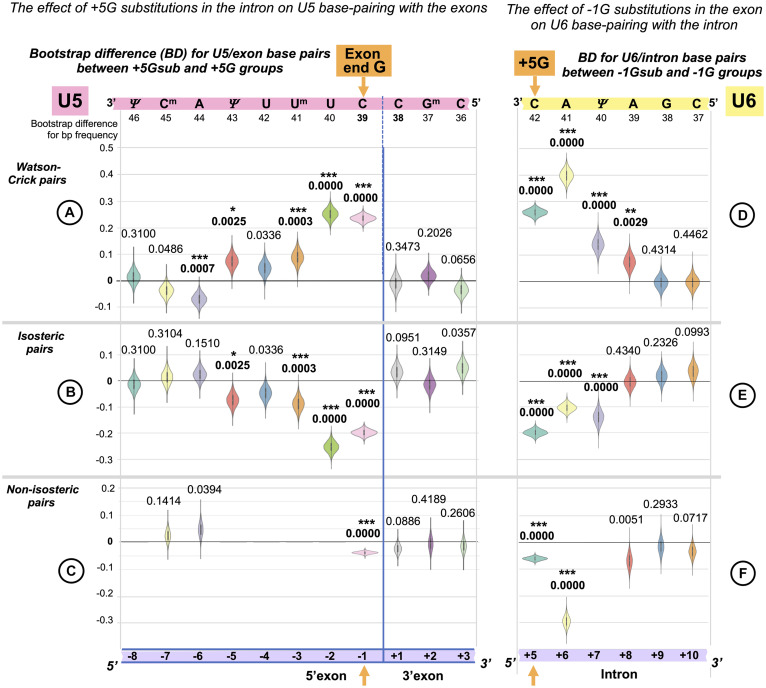
Reciprocal effects of +5G intron substitutions on the 5′ exon base pairs with U5 and the exon-end G substitutions on the intron base pairs with U6. Distributions of 10,000 bootstrap differences for the frequency of Watson–Crick, isosteric, and non-isosteric base pairs at each position of the exon junction (U5 biding site) between junctions flanking introns that conserve +5G and those where this base is substituted **(A–C)**. Differences for the bp frequencies of the U6 binding site at the start of the intron positions +5 to +10 between introns preceded by exons that conserve exon-end G (−1G) and those that do not **(D–F)**. The null hypothesis probability, *P*(*H*_0_), of no difference is indicated above each violin, and asterisks mark significant changes after the correction for multiple testing (see Materials and Methods for details).

We can make a more detailed comparison and test for differences in the frequency for each type of base pair at each of the 11 splice junction positions between our two datasets: +5Gsub and +5G, using a bootstrap procedure. Distributions of the bootstrap differences (BDs) are summarized in three sets of violin plots, one set for each base pair geometry: Watson–Crick, isosteric, and non-isosteric (Figures 5A–C). The grid line 0 represents the null hypothesis of no difference between the two datasets. The violin plots represent frequency estimates: the variations in the difference that could arise from the variation in the sampling of the transcriptome (i.e., the uncertainty associated with the observed differences for our dataset). The null hypothesis *p*-value is indicated (statistically significant values are marked with asterisks—see section “Materials and Methods” regarding multi-comparison corrections). The BDs above 0 indicate an increase inthe base pair frequency in the +5Gsub dataset compared to the +5G dataset.

In accordance with sKL divergence evaluation, there are no substantial (or significant) changes in the base pair composition for the 3′ exon with U5 C_38_G^m^_37_G_36_: all the violin plots to the right of the blue line representing the splice junction in Figures 5A–C adhere to the 0-difference lines. The picture is different for the 5′ exon: the violin plots to the left of the blue line show that the changes for the frequency of Watson–Crick pairs are largely mirrored by those of isosteric pairs (Figures 5A,B): these pairs replace each other in the interactions of U5 snRNA with the 5′exon. Non-isosteric pairs (Figure 5C), which are the minority (less than 15% of all pairs), play little role in these exchanges (see below). Significant increases in U5 Watson–Crick pairs with 5′ exon positions −1, −2, −3, and −5 are observed, which apparently compensate for the absence of the key U6 C_42_ = G_+__5_ pair. The decrease in Watson–Crick pairs at position −6 shows that a 5-bp-long U5 interaction is sufficient, if it is a perfect helix rich in Watson–Crick pairs. Conversely, introns with the conserved +5G are preceded with exons that form fewer proximal Watson–Crick pairs with U5 snRNA, and their helices have significantly more Watson–Crick pairs in position -6.

There are five violins missing in Figure 5C, as non-isosteric pairs do not exist for these positions. Five nucleotides out of 11 in the U5 snRNA Loop1 are uracils, which can form isosteric pairs with any base. The only change observed for non-isosteric pairs is that they completely disappear in position −1 if +5G is missing in the following intron.

##### The Effect of the +5G Is Specific to U5 Rather Than U1 snRNA Interaction

**FIGURE 6:**
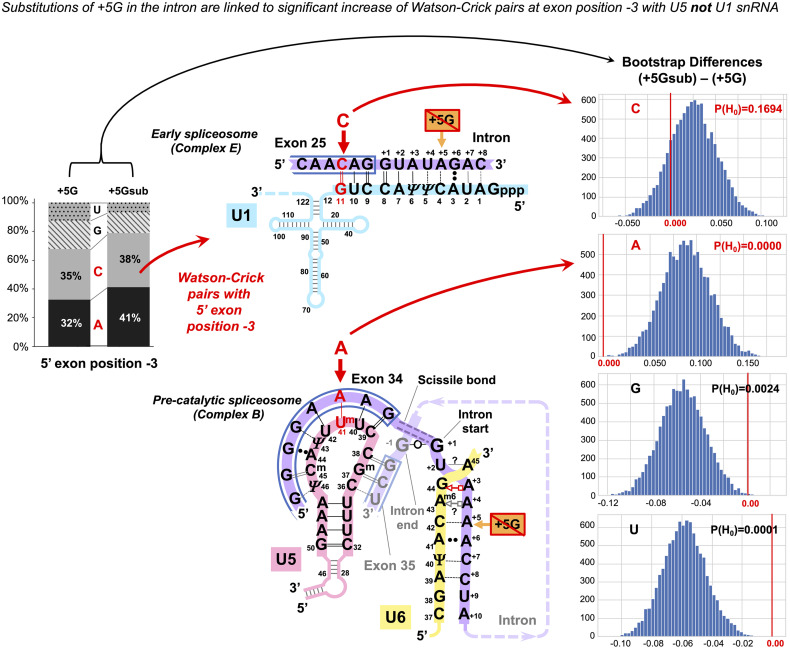
The +5G effect is specific to U5 snRNA, not U1 snRNA. The bar chart on the left shows the distribution of nucleotides for the 5′exon position −3 in the presence and absence of the conserved +5G at the start of the intron. The schematics show the different nucleotide preferences for U1 and U5 paired to exon position −3 (indicated in red). Histograms for distributions of 10,000 bootstrap differences for the frequency of each nucleotide at position −3 show that significant increase for A_–3_, creating a Watson–Crick pair in the U5 interaction, but not C_–3_ compensates for the loss of the U6 C_42_ = G_+__5_ pair.

In principle, it is possible that observed changes in U5 base pairs could be simply a consequence of the initial interaction of U1 snRNA with the 5′ splice site as this requires a threshold number of Watson–Crick pairs (5–6 bp, Ketterling et al., 1999), which can be on either side of the exon/intron boundary. The evidence against this argument is twofold: First, we observe an increase in Watson–Crick pairs at 5′exon position −5, and the initial interaction with U1 snRNA does not extend to this position. Second, U1 and U5 have different preferences for base-pairing at 5′exon position −3. We applied bootstrap analysis to nucleotide changes at position −3 linked to +5Gsub and +5G introns (Figure 6). There is a significant increase in the adenine required for forming Watson–Crick pairs with the U5 uracil U^m^_41_ and no significant change in cytosine for Watson–Crick pairs with U1 guanine G_11_. Thus, we can unambiguously link the observed changes to the interactions of U5 snRNA with the 5′exon.

##### U6 Watson–Crick Pairs With Intron Positions +5 to +8 Compensate for Substitutions of the Conserved −1G in the Preceding Exon

**FIGURE 7:**
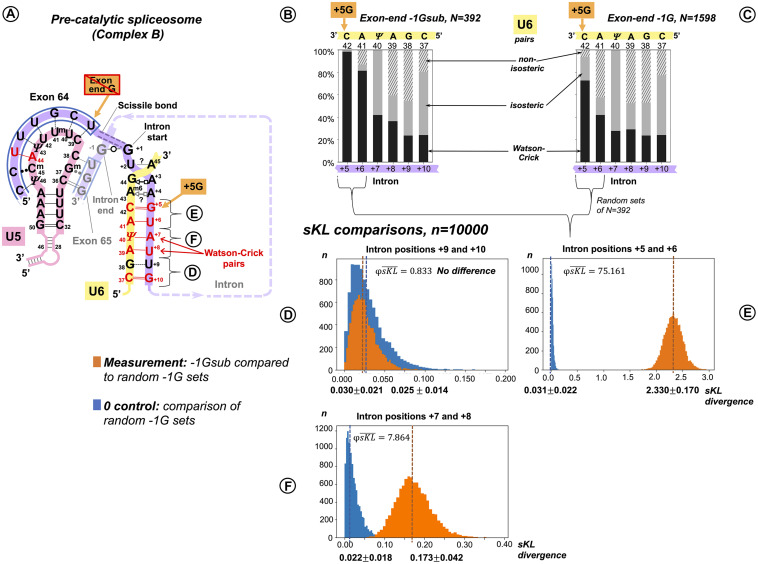
Additional U6 Watson–Crick pairs with the start of the intron at positions +5 to +8 compensate for substitutions of the conserved exon-end G. **(A)** Schematic of the examined interactions with U6 and U5 snRNAs (using human dystrophin intron 64 and the splice junction of exons 64 and 65 as an example). The U6 interaction with the intron positions +5 to +10 is subdivided into dinucleotides that correspond to sKL distributions **(D–F). (B)** Base pair type frequencies for 392 human introns preceded by exons lacking −1G (−1Gsub). **(C)** Base pair composition for 1,598 introns preceded by exons with conserved −1G. **(D–F)** Distribution of symmetrized Kullback–Leibler divergences (sKL) for each dinucleotide between the −1Gsub dataset and 10,000 random non-redundant −1G sets of *n* = 392 (orange histograms) compared to a control distribution of the same size between random non-overlapping and non-redundant subsets from the −1G dataset (blue histograms).

In a reciprocal experiment, we separated introns preceded by exons that conserved exon-end G (−1G) and introns preceded by exons with −1G substitutions (−1Gsub). For the sKL divergence comparison, we followed the same procedure as described for the exon junction (U5 binding site, +5G/+5Gsub, see above). Again, our two datasets were of unequal size: exons with conserved −1G, *N* = 1,598 and exons with substitutions −1Gsub, *N* = 392. Consequently, the control in this case is provided by pairs of random non-overlapping and non-redundant sets of size *N* = 392 drawn from the −1G dataset. One of the −1G sets from each pair was compared to the −1Gsub dataset. Investigating dinucleotide subsets, we observed a strong base pair type divergence corresponding to the loss of the exon −1G at intron positions +5 and +6, a smaller effect at +7 and +8, and no effect further downstream (Figures 7A,D–F). We can see an increase in Watson–Crick pairs in positions +5 to +8 in the absence of exon-end guanine (Figures 7B vs. 7C).

##### Functional Importance of Distinct U6 Base Pairs at Specific Positions

Exactly as we have done for the U5 binding site (see above), we applied bootstrap resampling to test the null hypothesis of zero difference for the frequency of each base pair type at each of the six intron positions +5 to +10. The result is summarized in three sets of violin plots (Figures 5D–F).

There is a rise in Watson–Crick pairs at positions +5 through +8 with the largest rise in position +6 (Figure 5D). The importance of these interactions downstream of position +6 only becomes apparent in the absence of −1G, with no detectable genomic conservation (Figure 1). The nature of changes is somewhat different from the reciprocal changes of the U5 binding site, as the increase in Watson–Crick pairs is accompanied by a significant decrease in both isosteric and non-isosteric pairs (Figure 5F). This is a result of non-isosteric pairs being tolerated at these positions in the dominant −1G case (Figure 7C), reflecting less constraints on the geometry of the helix for U6 binding with the intron than for U5 Loop1 presenting the exon junction in the catalytic core of the spliceosome.

##### Explaining Rare Introns Missing Both the Conserved +5G and End of Exon −1G

In our sample of 1,990 human introns, 6 (0.3%) lacked both these conserved guanines. We observed that multiple other Watson–Crick pairs stabilize both U5 and U6 interactions in these cases (Supplementary List S4).

#### The Effect of Intron −3C Substitutions at the 3′ Splice Site

The bootstrap difference analysis below shows that the 3′ intron end substitutions of the conserved −3C are supported by the increase in U5 Watson–Crick pairs with the 3′ exon position −1. This indicates a similar role of U5 in the correct recognition of the 3′ splice site, as at the 5′ splice site. The timing of the 3′ exon interaction with U5 and a possible RNA partner for intron position −3 are addressed in the Discussion.

##### U5 Watson–Crick Pairs With the 3′ Exon Compensate for Substitutions of the Conserved −3C in the Preceding Intron

**FIGURE 8:**
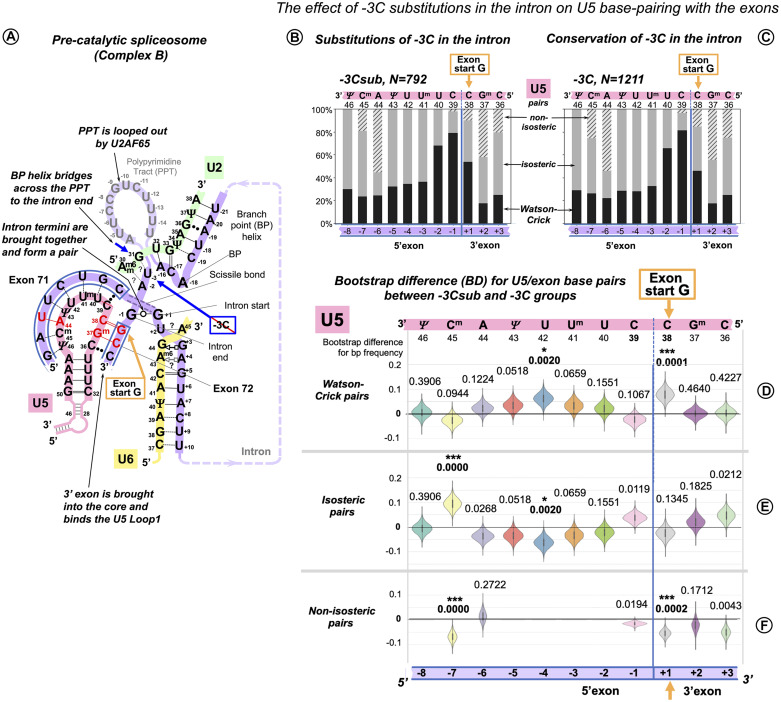
Additional U5 Watson–Crick pairs with the 3′ exon compensate for substitutions of the conserved −3C at the end of the intron. **(A)** Schematic of the examined interactions with U5 and U2 snRNAs (using human dystrophin splice junction of exons 71 and 72 and intron 71 as an example). We propose (see DISCUSSION) that BP helix bridges across the PPT to the end of the intron secured by the conserved −3C paired with U2 G_31_. This agrees with the previous mutation evidence (Brock et al., 2008; Corrionero et al., 2011), biochemical studies (Kent et al., 2003; Chen et al., 2010), and X-ray structures (Kent et al., 2003; Sickmier et al., 2006). The example here shows the U2 G_31_--U_–3_ pair instead, as dystrophin intron 71 lacks −3C. **(B)** Base pair-type frequencies for 792 human splice junctions related to introns lacking −3C (−3Csub). **(C)** Base pair-type composition for 1,211 splice junctions of introns with conserved −3C. **(D–F)** Distributions of 10,000 bootstrap differences for the frequency of Watson–Crick, isosteric, and non-isosteric base pairs at each position of the splice junction (U5 binding site). The null hypothesis probability, *P*(*H*_0_), of no difference between the two cases is indicated above each violin; asterisks mark significant changes after correction for multiple testing (see Materials and Methods for details).

Following up on our observation that in the human dystrophin gene the absence of −3C in the intron makes Watson–Crick pairs with the 3′ exon twice as likely, we sorted our sample of exon junctions of the major introns (*N* = 2,003, inclusive of introns with +2U substitutions, see above) into two subsets: −3Csub, *N* = 792 and −3C, *N* = 1,211 (Figure 8). In this case, the differences are smaller than for the previous comparisons (Figures 8B–F).

In the absence of −3C, U5 Watson–Crick pairs do increase at position +1 of the 3′ exon, replacing non-isosteric pairs and thus strengthening the U5 interaction with the 3′ exon. The 5′exon interaction with U5 shows an increased proportion of Watson–Crick pairs centered at position −4 and a rise in isosteric pairs at position −7 due to a drop in non-isosteric pairs, possibly indicating that stabilizing distal positions of the 5′exon helix is important for the intron complex overall (Figure 8A).

### U5 Watson–Crick Pairs With the 3′ Exon Promote Inclusion of Exons With +1G Mutations

The effect of human mutations of the conserved exon-end guanine (−1G) is currently explained by the base pairing with U1 snRNA, so we cannot use it as an evidence to support our new U5 model. Therefore, we concentrated on the mutations of the exon-start guanine (+1G). However, mutation databases do not document the effect of these mutations on splicing. Thankfully, Fu et al. (2011) specifically examined 14 mutations of the exon-start guanine and quantified their effect on exon inclusion using minigene constructs in human cells (HEK293). Each measurement was a mean result of a triplicate experiment. The authors report that six of these +1G mutations (in *LPL* exon 5, *HEXA* exon 13, *LAMA2* exon 24, *NEU1* exon 2, *COL6A2* exon 8, and *COL1A1* exon 23) did not have any effect on exon inclusion at all (percent spliced in, PSI = 100%). *PKHD1* exon 25 +1G → *T* mutation resulted in a cryptic 3′ splice site activation with 99% inclusion of a longer exon. On the other hand, the splicing effect for the other seven +1G mutations (in *CAPN3* exon 10, *CLCN2* exon 19, *EYA1* exon 10, *COL1A2* exon 37, *FECH* exon 9, *GH1* exon 3, and *CAPN3* exon 17) did not involve any cryptic sites and varied from 91% PSI to complete exon skipping (respectively). Variable branchpoint sequences did not offer a clear explanation; instead, Fu et al. (2011) proposed that long polypyrimidine stretch promotes exon inclusion in spite of +1G mutations. However, reducing the length of this stretch to 5 bp in *LPL* exon 5 minigene still produced PSI of 63–83% (depending on the position of pyrimidines). Only two pyrimidines in *HEXA* exon 13 minigene resulted in PSI of 59–69%. The observed highly variable efficiency of exon inclusion with +1G mutations and the fact that the length of PPT does not always provide a clear explanation points out that other factors are also involved: the BP helix and the conserved intron position −3 are also expected to contribute to splicing efficiency. Indeed, PSI was brought down to 7% for *HEXA* exon 13 minigene when −3C was substituted to G.

**FIGURE 9:**
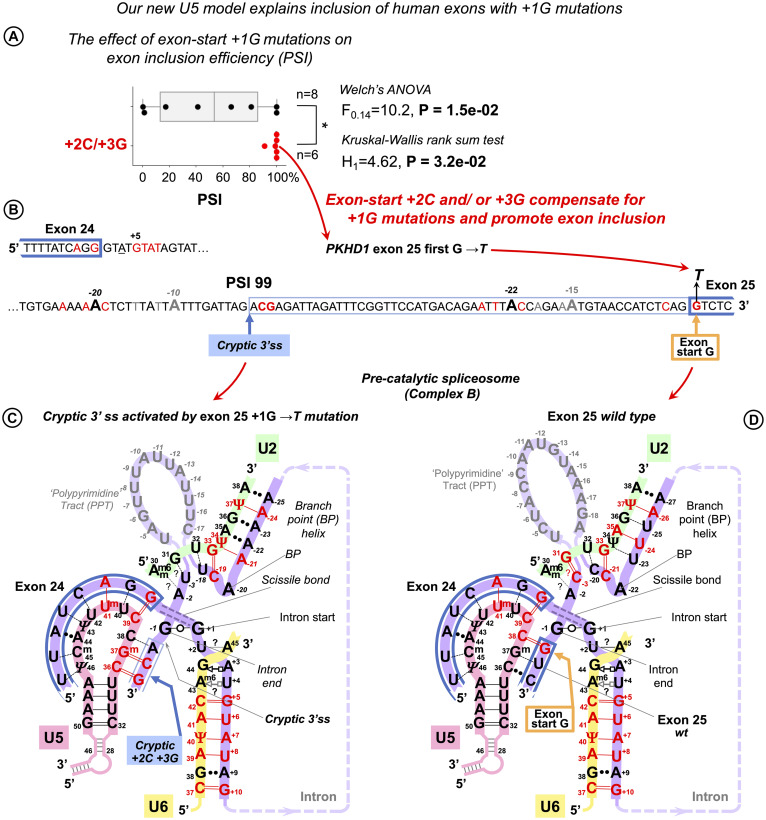
U5 Watson–Crick pairs with exon positions +2 and +3 promote inclusion of exons with +1G mutations. Fu et al. (2011) quantified the variable effect on splicing for 14 exon-start +1G mutations in human genes. **(A)** Boxplots show that exon inclusion (PSI, percent spliced-in) is strongly influenced by the presence of exon +2C or +3G. **(B)** Splice site sequences of the *PKHD* gene intron 24 and the flanking exons. +1G mutation in exon 25 completely blocks normal 3′ splice site and activates a cryptic 3′ss tag|ACG leading to the predominant inclusion of a longer exon and only 1% exon-skipping. **(C)** Base pairing scheme for the cryptic 3′ss with U5 snRNA Loop1 secured by U5 G^m^_37_ = C_+__2_ and C_36_ = G_+__3_ according to our new U5 model. **(D)** Base pairing of the normal wt exon 25 with U5 snRNA. +1G mutation abolishes the U5 C_38_ = G_+__1_ pair, which leads to exclusive use of the upstream cryptic 3′ss. **(C,D)** Recognition of all splice sites is complete in the pre-catalytic spliceosome (complex B) before *Brr2* promotes catalytic core formation (see DISCUSSION).

We re-examined the exon sequences for these 14 +1G mutations, looking specifically for cytosine in exon position +2 and guanine in exon position +3, because they form Watson–Crick pairs with U5 C_36_G^m^_37_ according to our proposed binding register. We found that cytosine occurs in position +2 in two mutations that did not affect splicing (PSI = 100%: *GH1* exon 3 and *FECH* exon 9) and in *CAPN3* exon 10 with 91% correct exon inclusion. Guanine occurs in position +3 in further two mutations with 100% PSI: *LAMA2* exon 24, *NEU1* exon 2. Finally, both +2C and +3G are involved in the cryptic 3′ss activated by +1G mutation in *PKHD1* exon 25. If we plot exon inclusion efficiency (PSI) for +1G mutations with +2C/+3G and without +2C/+3G, we can see that while the latter group is highly variable as can be expected if many factors are involved, the former group is clearly clustered at the top end, indicating that the presence of +2C or/and +3G is a very strong factor that promotes exon inclusion in spite of +1G mutations (Figure 9A). Although both ANOVA with Welch’s correction for unequal variances (the greater variance for the larger group makes false negatives more likely; McDonald, 2014) and non-parametric Kruskal–Wallis rank sum test show that there are significant differences between the means and locations of these two groups, statistical tests for *N* = 14 are implied only to complement the obvious differences between the boxplots (Figure 9A).

We further compared the effect of exon +2C/+3G with other factors that are expected to influence exon inclusion efficiency (Supplementary Figure S11 and Supplementary Table S4). Apart from the aforementioned PPS length (as in Fu et al., 2011), +1G → *A* mutation is much better tolerated than +1G → *T*, so a change for purine emerges as a second strongest factor after +2C/+3G, which is to be expected, as generally in the human exons, +1A is twice more likely than +1T. An example of the cryptic 3′ss activated by +1G mutation in *PKHD1* exon 25 is detailed in Figures 9B–D.

Identifying that exon +2C and +3G compensate for +1G mutations and strongly promote exon inclusion provides a clear explanation of the human mutation analysis and allows to conclude that the interaction of the 3′ exon with U5 Loop1 in the proposed binding register plays an important role in splicing precision. Moreover, this interaction of the 3′ exon, now confirmed by the mutation analysis, is possible only for the fully open 11 nt U5 Loop1 that we consider, as opposed to the 7nt version that prevails in Cryo-EM reconstructions (Discussion).

## Discussion

### The U5 Hypothesis Summary

#### Optimal Binding Register for Diverse Exons and U5 snRNA Loop1: The Exon Junction Is Positioned at U5 C_39_|C_38_

This U5/exons model is based on homologous interactions of a mobile Group IIA intron Id3 Loop with genomic retrotransposition sites in bacteria.

#### Common Mechanism of Base Pairing for U5 snRNA With Diverse Human Exons and *Ll*.LtrB Intron With New Loci in Retrotransposition

We suggest that these RNA loops recognize their variable target sequences by helix architecture, accepting Watson–Crick and isosteric base pairs and rejecting geometrically different pairs.

#### Significant Role of U5 snRNA in Specific Exon Recognition in the Pre-catalytic Spliceosome

U5 Watson–Crick pairs with the exons in the proposed binding register compensate for substitutions of the conserved intron positions. In addition, our binding register explains human mutation data: U5 Watson–Crick pairs with exon positions +2 and +3 compensate for +1G (exon-start) mutations and strongly promote exon inclusion.

This last point, based on statistical analyses of base pairs at specific positions and further supported by human mutation evidence, directly proves the first point that the exon junctions are positioned at U5 C_39_|C_38_ (The timing for the 3′ exon interaction with U5 Loop1 is specially discussed below). The second point on the geometric sequence recognition cannot be directly tested by statistics; however, it is our explanation of the observed common base pair types used by both RNA loops.

### Modeling U5 Loop1 Base-Pairing With Human Exons on Group IIA Intron Interactions With Retrotransposition Sites

**FIGURE 10:**
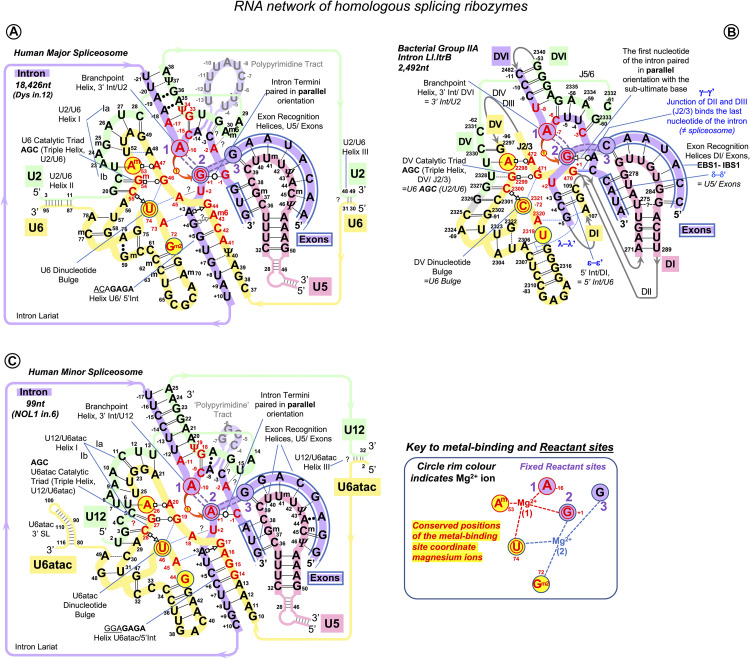
RNA network of the homologous ribozymes: human major and minor spliceosomes and Group IIA intron. First catalytic step, spliceosomal complex C (successive spliceosome complexes are detailed in Supplementary Table S1). The nucleophilic attack by the BP adenosine: curved red arrow. The intron breaks off the 5′exon end and bonds with the 2’O of the branching A: double purple dashed lines indicate the scissile (purple fill) and emergent (no fill) covalent bonds. Splicing catalysis requires two Mg^2+^ ions at a fixed distance from three reactant sites (Steitz and Steitz, 1993; Fica et al., 2013). At the first catalytic step, Mg^2+^(1) activates 2’OH of the BP A in the Reactant site 1. Mg^2+^(2) stabilizes the leaving 3’OH of the last nucleotide of the 5′ exon in Reactant Site 3. Both magnesium ions form a complex with the scissile phosphate of the N_+__1_ of the intron in Reactant site 2. **(A)** Human major spliceosome (intron 12 of the dystrophin gene as an example). The ribozyme is an assembly of three separate snRNAs with a record number of modified residues. The structure of the U6/U2 catalytic triplex is inferred from Keating et al. (2010) and Galej et al. (2016) and the U6/intron duplex as in Fica et al. (2017), see also Supplementary Table S3. Non-canonical RNA pairs are shown with Westhof geometric symbols (Leontis et al., 2002). Tertiary interactions as in Anokhina et al. (2013): blue dashed lines; Mimic Watson–Crick-like base pairing: black dashed lines; Base pairing with unknown non-Watson–Crick geometry: double dots. Base modifications as in Figure 1 and G^m2^: N2-methylguanosine. **(B)** LtrB Group IIA intron (*Lactococcus lactis*). Interactions of the catalytic triplex are extrapolated from the *O.i*. structure (Keating et al., 2010). Core motifs of this large RNA molecule are colored as homologous RNA components of the spliceosome. Greek letters: tertiary interactions in Group II introns, shown in blue. γ–γ’ and λ–λ’ interactions do not have homologs in the spliceosome. All other core interactions and catalytic structures of the ribozyme are labeled with spliceosome homologs in *italics*. Domains of the *Ll*.LtrB ribozyme: DI-DVI; Junctions between domains II and III or V and VI: J2/3 or J5/6. Double numbering is used for the residues starting from domain V, the negative number indicating the position from the 3′ end. **(C)** Human minor spliceosome (Tarn and Steitz, 1996a, b; Widmark et al., 2010; Edery et al., 2011; He et al., 2011; Younis et al., 2013; reviewed in Turunen et al., 2013). U5 is the only snRNA shared with the major spliceosome. A lot fewer residues are modified in U12 and U6atac snRNAs compared to U2 and U6 paralogs. Perfect conservation of the BP helix and the U6atac snRNA AAGGAGAGA box interaction with the 5′ intron end is characteristic of the minor spliceosome. (?): an odd U12 C_4_ bulge (see Supplementary Comment S1) here reproduced as in Tarn and Steitz (1996b) and Turunen et al. (2013). The minor introns are expression regulators of critical genes: the example here is intron 6 of the human **N**ucle**ol**ar Protein **1** (NOL1/NSUN1; Brock et al., 2008) gene encoding an RNA:5-methylcytosine-methyltransferase (known as proliferation antigen p120 overexpressed in virtually all types of cancer cells).

Our new model of the interactions of the exon junction with U5 Loop1 is inspired by the homologous interactions in *Ll.*LtrB, bacterial Group IIA intron (Figure 10).

Like Group II introns, human spliceosome is a metalloribozyme: protein-free small nuclear RNAs U6 and U2 are capable to catalyze splicing *in vitro* (Valadkhan et al., 2007; Jaladat et al., 2011). The core RNA components of the catalytic spliceosome resemble closely the domains of the Group II intron (Figures 10A,B): the branchpoint helix with the adenosine bulge, the intron termini pair with the parallel orientation of the RNA strands (specially discussed below), and the catalytic metal binding site (Keating et al., 2010; Fica et al., 2013; Galej et al., 2014, 2018; Nguyen et al., 2015; Zhao and Pyle, 2017a). The similarities are so great that the studies of the spatial organization and mechanism of pre-mRNA splicing are much in debt to the structural and genetic studies of Group II introns. Both in the spliceosome and in Group II introns, the two-step splicing mechanism (Steitz and Steitz, 1993) proceeds through the 2’O nucleophilic attack or “branching” of the sugar-phosphate backbone at the adenine base leading to the formation of an intron lariat excised after the exon ligation. Both steps of splicing are reversible. Group II introns use reverse splicing for retrohoming into the intronless alleles or retrotransposition into other genomic loci with sequence similarities (Griffin et al., 1995; Eskes et al., 2000; Ichiyanagi et al., 2002; Zhong and Lambowitz, 2003; Lambowitz and Zimmerly, 2011; Lambowitz and Belfort, 2015). Reverse splicing by the spliceosome was demonstrated *in vitro* (Tseng and Cheng, 2008) and suggested to be implicated for splicing quality control (Smith and Konarska, 2008).

In focus here are homologous U5 Loop1 and Group IIA Id3 Loop. Both these loops are 11nt long and contain five uracils. They bind both 5′ and 3′ exons aligned for ligation in the forward splicing process, and the exons to be separated by the intron precisely at the junction in the reverse splicing process. However, Group IIA intron self-splicing is based on near-perfect complementarity with the exons (Supplementary Figure S1). On the contrary, pre-mRNA splicing and Group IIA intron retrotransposition are equally challenged by variable exon junctions, and we looked for a common mechanism of sequence recognition by these homologous RNA loops.

The published data on the retrotransposition of the LtrB intron in *L. lactis* genome loci show without a doubt that the binding register for the Id3 Loop and the “exon” junctions in retrotransposition stays fixed and is the same as for the intron self-splicing: seven positions of the Id3 loop pair with the sequence upstream of the intron insertion as with the 5′ exon, and four positions of the loop form base pairs downstream of the retrotransposition site as with the 3′ exon (Ichiyanagi et al., 2002; Figures 2A,C). Retrotransposition sites are “similar” to the homing site in a sense that they have, on average, 55%–53% of sequence identity to the exon junction of the *L. lactis ltrB* gene interrupted by the LtrB intron. However, we gained a better insight into the mechanism of sequence recognition when we observed that the mismatched pairs are not random, and the preferred mismatches are limited to G--U/T--G, T--U, and C--U (Figure 2E).

By analogy, we manually aligned U5 Loop1 with the exon junctions for human dystrophin with maximum possible Watson–Crick pairs and the same preferred mismatches as for the Id3 Loop and found that indeed 95% of dystrophin junctions align to the same U5 positions and that the mismatched pairs are not random: C--U/U--C, A--C/C--A, G--U/U--G, and U--U are strongly preferred (Figures 2B,D,F). The mechanistic explanation for this preference is discussed in next section.

However, the first point of the U5 hypothesis, which we seek to prove by statistical analysis is that U5 Loop1 has a fixed optimal binding register for human exons: the end of the 5′ exon pairs with U5 C_39_, and the start of the 3′ exon pairs with U5 C_38_. This is contrary to the CryoEM models for U5 Loop1 of which the most recent places the conserved guanine at the end of the 5′ exon paired with U5 U_40_. Alignment of the interacting RNA sequences is an obvious starting point (surprisingly, it was never previously performed for U5 Loop1 and the exon junctions of pre-mRNA introns); however, the way to prove that our proposed U5 binding register is true can be by showing that it has a role in exon recognition, as is the case for the Id3 Loop of Group IIA introns. Our statistical analysis indeed shows this role: U5 Watson–Crick pairs with the exons in the proposed binding register compensate for substitutions of the conserved +5G and −3C in the intron splice sites (discussed below). Moreover, our model explains the effect of mutations in human exon sequences, which cannot be explained by Cryo-EM models (see further discussion).

### The Explanation for Acceptable Mismatches Can Be Base Pair Geometry

The geometric principle for variable exon junction recognition in Group IIA intron retrotransposition and pre-mRNA splicing was suggested by the mismatched pairs *Ll*.LtrB Id3 loop and human U5 Loop1 employ: G--U, U--U, A--C, and C--U. Bountiful literature on Watson–Crick-like geometry of these base pairs is very briefly discussed below.

**FIGURE 11:**
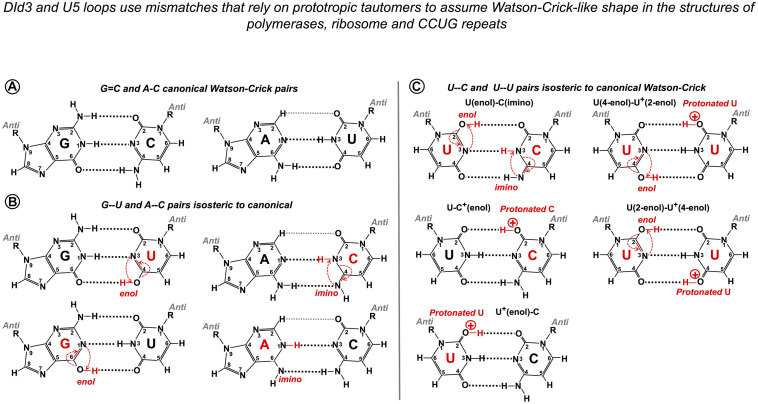
Watson–Crick-like geometry of G--U, A--C, C--U, and U--U pairs is supported by rare tautomerization and protonation. **(A)** Canonical Watson–Crick G = C and A-U pairs. **(B)** Predicted in the 1950s (Watson and Crick, 1953) and confirmed in 2011 by X-ray structures (Bebenek et al., 2011; Wang et al., 2011) Watson–Crick-like (isosteric to canonical) G--U pairs with either base in *enol* configuration and A--C pairs with *imino* tautomers of adenine or cytosine. Watson–Crick-like G--U is a high-frequency pair (NMR, Kimsey et al., 2015), which reflects the ease of proton repositioning provoked by the oxygen of the carbonyls. **(C)** Watson–Crick-like U--C and U--U pairs were reported by Rypniewski et al. (2016) in the XR structures of CCUG repeats, associated with the molecular pathology of myotonic dystrophy type 2. Possible configurations of U--C pairs are as in Rypniewski et al. (2016). The only configurations of the U--U pair that abolish the repulsion between the carbonyls and fit the reported structure (Rypniewski et al., 2016) are suggested here. Watson–Crick-like C--C pairs have not been reported; theoretical configuration requires imino tautomerization of one cytosine and protonation of the other (4-imino-C)--(2-enol-C^+^). Imino tautomerization is more difficult compared to enol, as the proton movement is between the two nitrogens.

In order to explain spontaneous mutagenesis in replication, Watson and Crick themselves put forward the idea that G--T or A--C pairs can assume dimensions of canonical pairs if one of the bases adopts its rare tautomeric configuration (Watson and Crick, 1953; Figures 11A,B). X-ray crystallography provided evidence of a G--T pair mimicking WC geometry in the active site of the human DNA polymerase λ and likewise an A--C pair adopting a clear WC-like shape within the active site of *Bacillus stearothermophilus* DNA polymerase I (Bebenek et al., 2011; Wang et al., 2011; reviewed by Kimsey and Al-Hashimi, 2014 as “high energy purine-pyrimidine base pairs”). Apart from provoking mistakes in DNA synthesis, the biological significance of mismatched pairs assuming WC geometry became further apparent when crystal structures of the codon–anticodon duplex of *Thermus thermophilus* 70S ribosome revealed that G--U mismatches in the first and second positions are isosteric to canonical pairs (Westhof, 2014; Westhof et al., 2014; Rozov et al., 2015). This finding proves that mispairs mimicking WC geometry are also responsible for translational infidelity. Mismatched pairs isosteric to canonical are recently discovered in the helix structures of accumulating human microsatellite expansion transcripts (reviewed in Błaszczyk et al., 2017). X-ray crystallography revealed WC-like C--U and U--U pairs stabilized by tautomerism or protonation (Figure 11C) in crystal structures of CCUG repeats associated with the molecular pathology of myotonic dystrophy type 2 (DM2, Rypniewski et al., 2016).

NMR analysis of synthetic RNA and DNA duplexes provided exciting evidence that G⋅U and G⋅T wobbles exist in dynamic equilibrium with short-lived WC-like G--U and G--T pairs, stabilized by tautomerization (one of the bases adopting a rare enol configuration) or ionization (one of the bases in anionic form; Kimsey et al., 2015). The authors estimate that these rare tautomeric and anionic nucleobases occur with probabilities 10^–3^–10^–5^ and imply the universal role of WC-like mispairs in routine cellular processes.

Here, we suggest that mimic pairs are routinely implicated in pre-mRNA splicing and Group IIA intron mobility. U5 and DId3 loops recognize their variable target sequences by helix architecture, accepting Watson–Crick and isosteric base pairs and rejecting geometrically different pairs, which perturb the helix architecture and make it unstable or incompatible with the spatial restrictions of the catalytic core. Although statistical testing cannot provide a direct proof of this second point of the U5 hypothesis, analysis of 2,000 human exon junctions shows that the base pairs that cannot support Watson–Crick geometry by prototropic tautomerization stay under 15% in the interactions of U5 and the exons, which means that, on average, there is only one such geometrically awkward pair per exon junction. Moreover, non-isosteric pairs are exceptionally rare in the 5′exon position −1, and cannot occur in positions −2 to −5, as these pair with U5 uracils, which are capable to form isosteric pairs with any other base, so the 5′ exon end normally has a perfect helix of at least 5 bp. In the absence of +5G in the following intron, significantly more Watson–Crick pairs are observed in these positions in place of isosteric pairs (statistical analysis discussed below). 3′ exon also very rarely has non-isosteric pairs in position +1, and in the absence of the conserved −3C in the upstream intron, there are significantly more Watson–Crick pairs in the exon position +1. Thus, generally geometrically awkward pairs occur in distal positions, and the shape of the U5 helix at the splice junction is preserved by isosteric pairs with prototropic tautomers, which allows for exon sequence diversity.

Finally, as we propose a mechanism that implies tautomerization of RNA bases, we remark that the predominant tautomers in RNA are a general convention for “physiological conditions” rather than a fact supported by evidence for the discussed U5 interactions with the exons in the spliceosomal ribozyme core. Tautomer diversity is often at the basis of RNA catalysis and ligand recognition, as demonstrated by structural studies of ribozymes, RNA aptamers, and riboswitches (reviewed in Singh et al., 2015).

### Statistical Testing of the New Model of the Interactions of U5 snRNA With Human Exon Junctions

We took advantage of our pilot investigation of the human dystrophin gene to plan our statistical analyses and looked specifically at the interactions of exons with U5 Loop1 linked to the introns that lack conserved positions +5G at the start and −3C at the end. We also took care to distinguish between U1 and U5 interactions with the 5′ exon, paying attention to the role of distinct positions.

**FIGURE 12:**
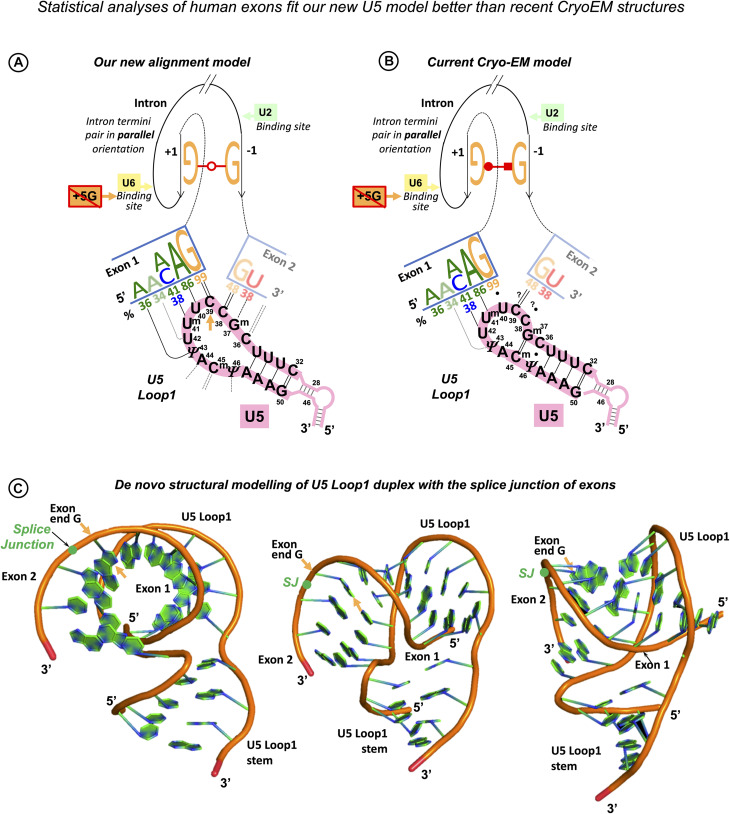
Our new U5 Loop1 interactions model compared to the current Cryo-EM model. The exon junction logo of the +5Gsub group (frequencies as in Figure 4B) reflects that substitutions of the conserved +5G in human introns are associated with the significant increase in −5A, −3A, −2A, and −1G in the 5′exon (Exon 1) and no changes in the sequence of the 3′exon (Exon 2)—compare to the exon junction logo for all human introns in Figure 1F. Here, we fit this +5Gsub exon junction logo alternatively to our new U5 model and the current CryoEM model and argue that the new model is a better match. **(A)** Our U5 Loop1 model is based on the initial alignment of human splice junctions with U5 Loop1 in parallel with the alignment of bacterial retrotransposition sites with the homologous *Ll.*LtrB Id3 loop. According to our model, substitutions of the conserved +5G in human introns are compensated by the additional Watson–Crick pairs with U5 Loop1 in the 5′ exon positions −1, −2, −3, and −5. In addition, our model explains the effect of mutations of exon-start G (Fu et al., 2011) by Watson–Crick base pairing of exon positions +2 and +3 with U5 Loop1 C_36_G^m^_37_ (Figure 9). The intron termini pair is shown in the configuration of the second Westhof geometric family in agreement with the previous mutation analyses (Scadden and Smith, 1995). This pair must be formed in the pre-catalytic spliceosome (complex B) to play a central role at the transition stage (complex C*). The intron termini pair brings the 3′ Exon 2 in contact with U5 Loop1 in the pre-catalytic spliceosome (see DISCUSSION). **(B)** The CryoEM model for U5 that currently prevails features a 7nt Loop1 and places the 5′ exon paired with U5 U_40_U^m^_41_U_42_ in the pre-catalytic complex B (Zhang et al., 2017, 2018, 2019). This eliminates the energy benefit of the G = C pair for the 82% conserved intron-end G. Accordingly, +5G substitutions are only supported by the increase in A-U pairs in exon positions +2 and +3. The intron termini pair was captured only in the post-catalytic spliceosome (complex P, Zhang et al., 2017), although the authors suggest that it must be present at the transition stage (complex C*). The configuration of this pair corresponds to the third Westhof geometric family, which is not consistent with the previous mutation analysis, as the covariant A⋅⋅C pair or compensatory A⋅⋅A and I⋅⋅I pairs are impossible in this configuration (Scadden and Smith, 1995, see DISCUSSION). Base pairing for the 3′ exon is still not resolved (Zhang et al., 2019). We placed 3′ exon aligned with only two possible unpaired positions of the U5 Loop1 (base pairing with question marks). However, exon +2C or +3G cannot form Watson–Crick pairs with U5 Loop1 in this binding register, so the fact that exon +2C/+3G promotes inclusion of exons with +1G mutations cannot be explained by the CryoEM model. The 7nt U5 Loop1 is too small to accommodate specific interactions with both exons. **(C)** Our *de novo* structural model of the U5 Loop1 duplex with the splice junction of exons. We used hypothetical exons complementary to U5 Loop1, while our comparison with the *Ll.*LtrB Id3 loop suggests that the real exon junctions form Watson–Crick-like pairs to fit diverse sequences and preserve the shape of the helix. Remarkably, a turn of the A-helix contains 11 bp, so the 11nt loop as shown here can well accommodate specific interactions with both exons. The U5 Loop1 helix appears hollow along the axis, which is typical of the A-helix (Heinemann and Roske, 2020).

We generated datasets of U5 and U6 base pairs in the interactions of 2,000 human splice junctions and their introns and analyzed these datasets for correlated base pair variation at specific positions. sKL divergence shows that +5G substitutions in the introns are associated with changes in the distribution of U5 base pairs with the 5′ exon, but not with the 3′ exon. While sKL divergence is the largest for 5′exon positions −1 and −2, which also pair with U1 snRNA in the early spliceosome, there is some divergence for 5′ exon positions −3 to −5. U1 does not bind positions −4 and −5 and selects a different base in position −3. Therefore, we observe the change in the distribution of U5 base pairs. Reciprocally, sKL divergence indicates that exon-end G substitutions are linked to changes in the distribution of U6 base pairs in the following intron positions +5 to +8. Divergence is largest for positions +5 and +6, and there is some divergence for positions +7 and +8, while positions +9 and +10 do not show a change in base pair distributions. We then enhanced the resolution of our analysis by bootstrap resampling of each U5 base pair frequency at each individual position of the exon junction and calculated bootstrap differences between splice junctions linked to introns that either carry +5G substitutions or conserve +5G. We found a significant increase in U5 Watson–Crick pairs with 5′ exon positions −1, −2, −3, and −5. Positions −3 and −5 indicate that this effect is specific to U5 rather than U1 snRNA. For the sake of comparison, we re-aligned the 5′ exon with U5 Loop1 according to the most recent Cryo-EM model, which means the loss of Watson–Crick pairs in positions −1 and −5. The lack of U6 C_42_ = G_+__5_ pair is compensated only by the increase in A-U pairs in 5′ exon positions −2 and −3 without the superior energy benefit of the G = C pair in exon position −1, which is an argument in favor of our new model (compare Figures 12A,B).

Furthermore, bootstrapping U6 base pair frequency at intron positions +5 to +8 shows significant increase in Watson–Crick pairs linked to substitutions of exon-end G. We conclude that U5 and U6 snRNAs collectively ensure the precise definition of the exon–intron boundary and mutually compensate for their variable splice sites by Watson–Crick base-pairing to stabilize the pre-catalytic complex.

We continued to examine the 3′ intron/exon boundary by bootstrap resampling of U5 base pair frequency at each individual position of the splice junction and found a significant increase in U5 Watson–Crick pairs with 3′ exon-start position +1 linked to the introns with substitutions of conserved −3C. This result shows that the U5 interaction with the 3′ exon is important for splicing fidelity.

### Splicing Effect of Human Exonic Mutations Can Be Explained by Our U5 Interactions Model

The effect of mutations of the 82% conserved exon-end guanine can be currently explained by base-pairing with U1 snRNA in the early spliceosome complex, which makes it difficult to prove the importance of base-pairing with U5 snRNA in the pre-catalytic complex. However, our new U5 model is the first clear base-pairing scheme for the exon-start guanine, which is 50% conserved in humans. We seek to connect our U5 interactions model with the real mutation data. Fu et al. (2011) examined the effect of 14 exon-start G mutations on exon inclusion using minigene constructs in cultured human cells and report highly variable PSI ranging from 0 to 100%. The authors suggested that the length of the polypyrimidine stretch is an explanation for the observed variation. However, mutant exons with 100% PSI were included persistently at 59–83% efficiency even if most of these pyrimidines in the preceding intron were changed for purines. While we expect that the efficiency of exon inclusion also depends on the BP sequence and conserved intron position −3, we opted to check if the exon interaction with U5 snRNA has an influence on PSI. We re-examined the exon sequences from Fu et al. (2011) according to our scheme of base-pairing with U5 snRNA and found that the presence of our predicted Watson–Crick pairs at exon positions +2 and +3 emerges as a strong factor that promotes exon inclusion in spite of +1G mutations (Figure 9A). These real mutation data have two important implications: they support our proposed binding register of the exon junction and U5 snRNA and require fully open 11nt U5 Loop1 for compensatory pairs with +2C and +3G (Figures 9C, 12A,C). On the contrary, we cannot explain these mutation data according to the recent CryoEM reconstruction of U5 Loop1 (Zhang et al., 2019). Base-pairing with the exon start (or 3′exon, Exon 2) has not been resolved yet; however, if we align the exon start to U5 C_38_C_39_ left unpaired because of the 5′exon shifted by one nucleotide in the 7nt loop, exon +2C or +3G cannot provide any obvious energy benefit to this structure (Figure 12B).

### Structural Model of U5 Loop1 Interactions With the Exons

We suggest that specific binding of both exons by U5 can be spatially resolved only if Loop1 extends to 11nt, as a turn of the A-helix accommodates 11 bp (recently reviewed in Heinemann and Roske, 2020). To demonstrate this, we created a *de novo* structural model of U5 Loop1 using the simRNAweb server (Magnus et al., 2016)^2^, which features a hypothetical exon junction complementary to the loop, while the real exon junctions will contain mimic Watson–Crick-like pairs to preserve the geometry and the overall helix architecture (Figure 12C). Our challenge now is a structural model of U5 and the exons before the ligation, which should include the intron termini pair. The configuration of this universally conserved pair is specifically discussed below.

In this study, we examine pre-mRNA interactions with snRNAs and in particular the role of U5 snRNA interactions with the exons on splicing precision, leaving out the role of protein components of the spliceosome in stabilizing the exon interactions. CryoEM studies confirm that the 273.6-kDa U5snRNP protein Prp8 stabilizes U5 Loop1 interactions with the variable exon sequences (Galej et al., 2016; Bai et al., 2017; Wan et al., 2020; Wilkinson et al., 2020). Remarkably, Prp8, the most conserved large nuclear protein in eukaryotes, includes reverse transcriptase (RT) Fingers/Palm, Thumb, DBD/Linker, and endonuclease domains homologous to Group II intron encoded protein (IEP), which also participates in exon recognition (Lambowitz and Belfort, 2015; Fica and Nagai, 2017; Zhao and Pyle, 2017b; Wan et al., 2020). While the conservation of human exons, our statistical analyses, and human mutation data show that the exon ends bind U5 C_38_|C_39_ and the specific U5 interactions with both exons play a key role in splicing precision, still U5 Loop1 inevitably forms multiple mismatched pairs. Most of these mismatches may assume the configuration of Watson–Crick by prototropic tautomerization and therefore rely on special electrostatic conditions to preserve a perfect A-helix shape. It is reasonable to suggest that Prp8 that envelops U5 Loop1 supports these isosteric pairs to ease the recognition of the multitude of diverse exons. To visualize the specific recognition of exons in the pre-catalytic complex, it will be necessary to integrate our new U5 Loop1 model with the CryoEM structure of spliceosome protein components.

### The New U5 Model Implies Changes to the Putative RNA Network in the Spliceosome

Statistical analysis of human exon and intron sequences and the available human mutation data show that the interaction of U5 Loop1 with the 3′ exon is important for splicing fidelity. The effect of −3C substitutions (see RESULTS) suggests that at the 3′ intron/exon boundary, a similar mechanism is at work to that of the 5′exon/intron boundary. If so, what is the RNA partner for intron position −3? When does the collective recognition by U5 and this other RNA partner occur? Hence, we are obliged to discuss the interactions of the 3′ intron end and the timing of the 3′ exon interaction with U5.

#### U2 snRNA Pairs With the 3′ Intron End Skipping the PPT in Spliceosomal Complex A

In the spliceosome, the branchpoint adenosine is distanced from the intron end by the highly variable polypyrimidine tract, a protein interface for alternative splicing regulation, which does not belong to the ribozyme core. A solution first proposed by Kent et al. (2003) (on the basis of the Fe-EDTA probing of the U2AF^65^/RNA interactions and a comparison with X-ray structures of related RNA Recognition Motifs (RRM) is that “U2AF65 bends the RNA to juxtapose the branch and 3′ splice site.” This model is in agreement with the later X-ray structure of U2AF^65^ bound to polyU, which reports 120° kink in the RNA strand (Sickmier et al., 2006). Indeed, the flexibility of the RNA chain is essential for U2AF^65^ binding, as it is blocked if uracils in the PPT are converted to pseudouridines (Chen et al., 2010), which conveys rigidity to the sugar-phosphate backbone (Charette and Gray, 2000). In fact, U2 snRNA binds the branchpoint site only after U2AF^65^ appropriately shapes the PPT. It can be explained if we imagine that U2 bridges across the looped-out PPT and pairs with the end of the intron. Crucially, human mutation analysis indicates the involvement of the conserved intron −3C in the BP helix. Corrionero et al. (2011) explored the underlying mechanism of splicing failure caused by −3C substitutions in intron 5 of the *Fas/CD95* gene (a jammed apoptotic receptor switch in T cells leads to **A**utoimmune **L**ympho**p**roliferative **S**yndrome (ALPS). They demonstrated that while the U2AF^65^ binding efficiency is not affected, substitutions of −3C block the U2 snRNA binding. The nucleotide distribution at position −3 in human introns (Figure 1A) is consistent with the co-variation of C_–3_ = G/U_–3_--G pairs, which points at U2 G_31_ as the only possible partner base (Figure 10A), doubled by U12 G_16_ in the minor spliceosome, a paralogous complex that processes 0.4% of human introns (Figure 10C). The question remains if the invariant A-_2_ can pair with U2 A^m,m6^_30_ (2’O-methyl,N6-methyladenosine). However, the Hoogstein edge of A_–2_ interacts with BP A according to CryoEM (Wilkinson et al., 2020). In the minor spliceosome that has a lot fewer base modifications in snRNAs, the U12 U_15_-A_–2_ pair is a perfect match (Figures 10A,C). Regardless of the possible A_–2_ partners, the proposed U2 G_31_ = C_–3_ pair explains the need for the protein co-factors SF1 and U2AF^65,35^ to bind the branchpoint, polypyrimidine tract, and the 3′ intron end before the U2 RNA component: PPT needs to be looped out to allow the U2 snRNA to bridge across it to the end of the intron. Extending the BP helix beyond the variable PPT brings the bulged adenosine at a fixed distance of 4nt from the intron termini pair. Accordingly, in Group IIA introns, 4 bp is a conserved distance between the BP A and the pair formed by the first nucleotide of the intron and the sub-ultimate base (bacterial *Ll*.LtrB intron—Figure 10B, Group IIA introns in general—Zimmerly Lab Group II intron database^1^; Candales et al., 2012).

#### Intron Termini Pair Is Formed and the 3′ Exon Binds U5 Loop1 in the Pre-catalytic Spliceosome (Complex B)

**FIGURE 13:**
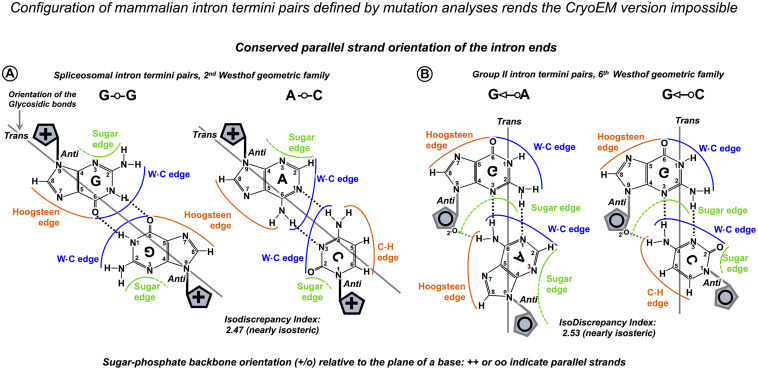
Interactions of the intron termini are base pairs with parallel strand orientation. **(A)** The configuration of mammalian intron termini pairs was defined by mutation analyses (Scadden and Smith, 1995) as G⋅⋅G: N1, carbonyl symmetric, A⋅⋅C: reverse wobble, both corresponding to the second Westhof geometric family. CryoEM configuration corresponds to the third geometric family, which is impossible for A⋅⋅C pairs (for further explanation, see text). **(B)** Base pair configuration for Group II intron first and sub-ultimate nucleotides captured in the recent crystal structure (after Costa et al., 2016, confirmed by personal communication with Professor Eric Westhof) and shown here with additional hydrogen bonds formed by 2’O of the riboses (after Leontis et al., 2002). Parallel strand orientation is characteristic of these pairs. Ribose is located on the perpendicular plane and is shown as a schematic blue pentagon with **+** and **o** indicating the opposite directions of the sugar-phosphate backbone. For further explanation, see Supplementary Figure S3. The IsoDiscrepancy index is a numerical measure of geometric similarity (isostericity) of base pairs (online RNA base pair catalog, http://ndbserver.rutgers.edu/ndbmodule/services/BPCatalog/bpCatalog.html).

The principal interaction between the bases at the intron termini provides the necessary structural link for the transition between the two catalytic steps of splicing. This interaction is universally conserved in all Group II and pre-mRNA introns and involves non-Watson–Crick base pairing (Chanfreau and Jacquier, 1993; Parker and Siliciano, 1993; Chanfreau et al., 1994; Scadden and Smith, 1995). In eukaryotic introns, the first and last guanines form such a pair; however, human introns occasionally accommodate A⋅⋅C in place of G⋅⋅G (Table 1B). Compensatory double mutation analysis showed that it is also true for *S. cerevisiae* introns (G⋅⋅G can be exclusively substituted for A⋅⋅C; Parker and Siliciano, 1993; Chanfreau et al., 1994). Scadden and Smith (1995) explored the exact configuration of the intron termini pair in mammalian introns and showed that substitution of guanines for inosines does not affect the pair formation. Inosine is a guanine analog that lacks the N2-amino group, which means that −*NH*_2_ hydrogen bonds are not involved in the pair configuration. In addition, it appears that A⋅⋅A also weakly supports splicing. The predicted configuration that does not involve N2-amino groups of guanines and allows G⋅⋅G to be exchanged for A⋅⋅C and A⋅⋅A involves H-bonds between Watson–Crick edges with the *trans* orientation of glycosidic bonds and parallel sugar-phosphate backbone orientation (Figure 13A, explanatory Supplementary Figure S3) as opposed to the *cis* glycosidic bonds orientation of the canonical Watson–Crick pairs with antiparallel strands orientation (Supplementary Figure S4). CryoEM studies (Bai et al., 2017, reviewed in Wilkinson et al., 2020) differ from the configuration predicted by mutation analyses as the Watson–Crick edge of intron G_+__1_ appears to form H-bonds with the Hoogsteen edge of intron G_–1_ (with glycosidic bonds in *cis* orientation and parallel strands). This is problematic, as this configuration involves the N2-amino group of G_+__1_ and A⋅⋅C or A⋅⋅A does not exist in this configuration (third geometric family according to Westhof classification---online RNA base pair catalog)^3^.

**TABLE 1:** Intron termini pairs conserve parallel local orientation of the RNA strands.

(A) Examples of different pairs between the first and sub-ultimate bases of Group II introns*.				

Base pairs	Group IIA	Group IIB	Group IIC	Compensatory mutations
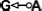	*Ll*.LtrB aI1 (*S.c.* cox1) aI2 (*S.c.* cox1) *O.i.*I2	ai5γ *Avi.*groEL (*A.v.*I1) *E.c.*I3, I5, I8 *Tel*3c, 4c, 4f (*Th.el.*)	*O.i.*I1 *A.v.*I2 *E.c.*I7 *Sr.me.*I5	
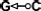		RmInt1 (*Sr.me.*I1) *E.c.*I2		
U-G		*Tel*4h		Chanfreau and Jacquier, 1993
C-G				Chanfreau and Jacquier, 1993
G-U		*Sr.me.*I2		

(B) Occurrences of different base pairs at the intron termini in humans**.				

**Base pairs**	**All human introns**	**Major (U2) spliceosome**	Minor (U12)*** spliceosome	**Compensatory mutations**

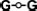	**99%**	**99%**	**83%**	
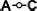	**1%**	0.01%	**13%**	
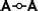	0.01%		1%	Scadden and Smith, 1995
A-G	0.01%	0.003%	1%	
A-U	0.005%		0.7%	
U-G	0.005%	0.005%		
G-A	0.004%	0.005%		
G-U	0.003%	0.002%	0.3%	
G-C	0.0004%	0.0005%		

*Eskes et al. (1997, 2000), Ferat et al. (2003), Plante and Cousineau (2006), Toor et al. (2008), Mohr et al. (2010), Chillón et al. (2014), Somarowthu et al. (2014), and Zimmerly Database (http://webapps2.ucalgary.ca/~groupii/). **Based on Sheth et al. (2006) and Parada et al. (2014). ***Minor spliceosome (U12) processes less then 0.4% of all human introns (Sheth et al., 2006).

**FIGURE 14:**
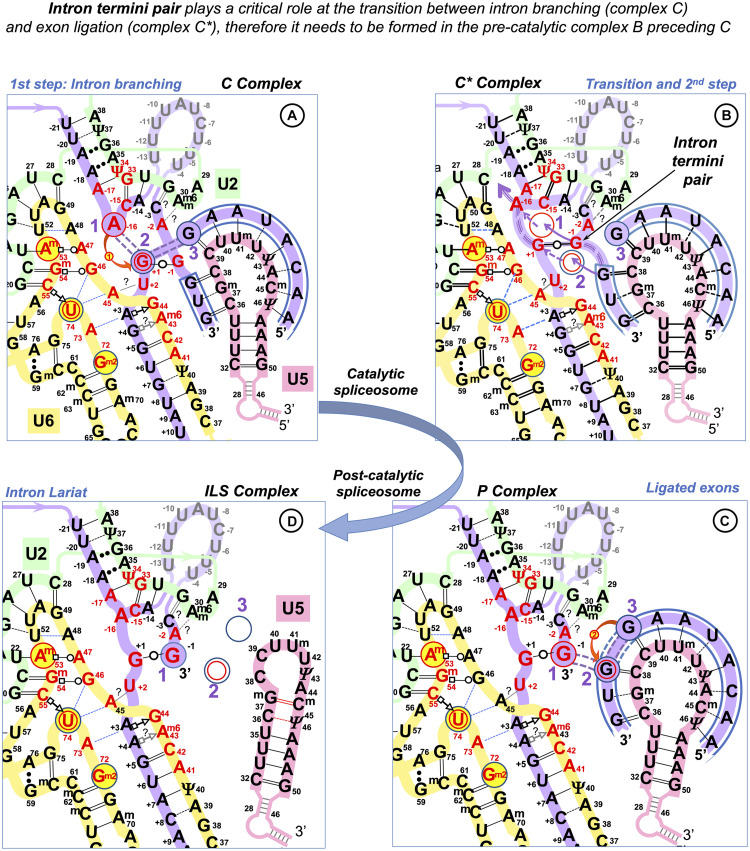
The central role of the intron termini pair in the transition between the two catalytic steps of splicing. The bond between the intron and the 5′exon is broken at the first catalytic step, and the formation of the covalent bond between the BP adenosine and the 5′ end of the intron (branching) triggers a rotation of the BP helix on its axis (Somarowthu et al., 2014; Bertram et al., 2017a). The correct repositioning of the branched intermediate absolutely requires U6 non-Watson–Crick pair(s) at the start of the intron at position +3 and/or +4 (Konarska et al., 2006). The momentum of the revolving BP helix is transmitted by the intron termini pair and drives the translocation of the 3′ exon. This movement is enabled by the relaxation of the U5 Loop 1 due to the disruption of the covalent bond between the intron and the 5′ exon. The overall configuration of the U6/U2 metal binding site stays unchanged, and the reacting residues are transitioned to the fixed Reactant sites (Steitz and Steitz, 1993; Fica et al., 2013; Semlow et al., 2016— Supplementary Comment S2). **(A)** RNA network of the first step spliceosome—as in Figure 10. **(B)** The RNA–RNA interactions at the moment of transition between the two steps of splicing: The biggest purple arrow indicates the repositioning (rotation) of the BP adenosine after branching, and the purple triple dashed line shows the transmission of the motion *via* the intron termini pair to the 3′ exon. Dashed purple arrows trace the movement of the residues out of the Reactant Sites 1 and 2, and the incoming nucleotides follow the path of continuous purple arrows. The position of the 5′ exon is unchanged in the Reactant Site 3. For the second reaction, the metal ions reverse their actions: the Mg^2+^ (2) activates the 3’OH group of the 5′ exon in Reactant Site 3, an attack is launched at the 5’PO_4_ of the 3′ exon in Reactant Site 2, while Mg^2+^ (1) stabilizes the leaving 3’OH of the last nucleotide of the intron at Reactant Site 1. The fact that the two metal ions play the activation role in turn enables a single catalytic core to accommodate both steps of splicing and also implies the ease of the reverse process (Fica et al., 2013). Although the second reaction (*curved red arrow 2*) happens in the C* complex following the transition, here the emergent covalent bond is shown in the successive P complex. **(C)** RNA network after the second catalytic step. Double purple dashed lines indicate the emergent (purple fill) and previous (no fill) covalent bonds. The exons are ligated and are still paired with U5 Loop1. The intron lariat stays paired with U6 and U2 snRNAs (Zhang et al., 2019). **(D)** The joined exons are disassociated from U5 Loop1 by Prp22 (Wan et al., 2017; Zhang et al., 2019); without RNA partners, the loop changes to “closed” (7nt) conformation. The intron lariat stays paired to U2/U6 and associated with U5 snRNP. The ILS complex is homologous to Group II intron RNP ready for reverse splicing (RNA network dynamics of successive spliceosomal complexes is summarized in Supplementary Table S1).

Group II intron ends are joined by base-pairing of the first and sub-ultimate nucleotides. This is often the G⋅⋅A pair featured in diverse introns of IIA, IIB, and IIC subclasses (Table 1A), and the configuration of this pair was captured by X-ray crystallography (Costa et al., 2016; Figure 13). Although the position of the Group II intron termini pair is shifted to the sub-ultimate nucleotide and the pair itself is different from eukaryotic introns, the evolutionary conserved feature is the parallel strand orientation of the intron ends. Plausibly, this conformation brings the exons together and supports splice junction binding to U5 Loop1 (or DId3 loop). Certainly, the quintessential intron termini pair is central for the two-step splicing mechanism, as it provides a structural link necessary for the transition between the intron branching and the exon ligation. Once the intron breaks off from the 5′ exon and the branchpoint helix rotates on its axis (Somarowthu et al., 2014; Bertram et al., 2017a), the 3′ exon is towed into the reactant site by the intron termini pair (Figure 14). In order to be functional at the transition stage, this link must be formed at the pre-catalytic stage.

The formation of the intron termini pair and the earlier involvement of the intron end −3C in the branchpoint helix guarantee the proximity of the 3′ exon to U5 at the pre-catalytic stage. In fact, 3′ exon pairing with U5 Loop1 can be responsible for the transformation of the Loop from the closed 7nt conformation, which it is likely to adopt without RNA partners, to the fully open 11 nt form, which can also accommodate the extended 5′ exon helix.

#### Overall Arrangement of Pre-mRNA Before the Catalytic Activation of the Spliceosome Supports Splicing Precision

The intron termini pair and the 3′ exon pairing with U5 in the pre-catalytic spliceosome imply that all the core snRNA interactions with pre-mRNA splice sites are already formed prior to the remodeling by the *Brr2* helicase and the configuration of the catalytic Mg^2+^ binding site. The trigger for *Brr2* activation is considered to be the U6 helix with the start of the intron (Charenton et al., 2019), which is imperfectly conserved in humans. Our statistical analysis of human splice sites uncovers the mechanism that compensates for this variability of the intron start: the stability of the U6 helix depends on the Watson–Crick pairs that the end of the exon forms with U5 snRNA. Similarly, U5 Watson–Crick pairs with the start of the exon stabilize substitutions at intron position −3, which we suggest pairs with U2 G_31_. In addition, mutations of the exon-start G are compensated by U5 Watson–Crick pairs with exon positions +2 and +3. Re-considering the variation of the splice site sequences and their collective recognition by snRNAs in the pre-catalytic complex, U6 helix cannot be the sole activation trigger. More likely, the overall stability of all the recognition helices between the substrate and U6, U5, and U2 snRNAs in the pre-catalytic complex B is a fidelity checkpoint for spliceosome activation (Successive spliceosome complexes are detailed in Supplementary Table S1).

### The New U5 Model Agrees With Increasing Exon Sequence Diversity During Protein Evolution

The evidence of alternative conservation of the intron and exon consensus in higher eukaryotes was presented previously as the evolutionary migration of the splicing signals from exons to introns. Indeed, molecular evolutionists had long identified that “old” introns have a conserved intron consensus and “new” introns, on the contrary, have a conserved exon consensus (Sverdlov et al., 2003). In view of the U5 hypothesis, gradual replacement of Watson–Crick pairs with isosteric mismatches has provided more diversity of the U5 binding sites, relaxing constraints for the sequence of the exons and aiding protein evolution. This process was supported by the conservation of specific Watson–Crick pairs at the U6 and U2 binding sites in the introns, ensuring the preservation of the impeccable splicing fidelity.

### The Need of Mutation Analyses of the U5 Interactions With Human Exons and the Proposed U2 Interaction With the 3′ Intron End

Experimental validation of the U5 hypothesis requires checking base-pairing by double mutagenesis, which aims to introduce covariant pairs between the interacting RNAs. A Watson–Crick pair is geometrically interchangeable for another Watson–Crick pair; however, if we account for the energy benefit, it is better to swap G and C between the interacting RNAs rather than introduce an A-U pair.

We do not know if changes in the U5 sequence will affect the conformation of Loop 1 or cause a shift in the binding register. Presumably, diverse sequences of Id3 loops in Group IIA introns (Supplementary Figure S1A) follow the same spatial scheme. As a precaution, while subjecting one exon interaction with U5 to double mutagenesis, it might be safer to choose another exon complementary to U5 Loop1 to secure the position of the exon junction.

#### 3′Exon and U5 Loop1

It is best to start with the 3′ exon binding register to avoid the ambiguity of U1 binding at the 5′ splice site. As we observed that exon +2C/+3G promotes the inclusion of exons with +1G mutations, we suggest changing nucleotides at exon positions +2 and +3 in minigenes from the study of Fu et al. (2011). For example (Figure 9B), will introducing +2C and +3G into exon 25 of the *PKHD* gene and removal of +2C, +3G at the cryptic 3′ss suppress the effect of the +1G → *T* mutation and re-activate the normal 3′ss? Furthermore, to verify base pairing, changes of exon positions +2 and +3 in the minigenes can be combined with mutations at positions 36 and 37 of Loop1 in a U5 expression construct and followed by co-transfection and quantification of the splicing outcome in human cells.

#### 5′Exon and U5 Loop1

Mutation analyses for the 5′ exon interaction with U5 is complicated by the initial U1 interaction across the exon/intron boundary. Presumably, nucleotide changes at the exon end will not block U1 binding if we make sure that complementarity to U1 extends over 5–6 base pairs overall (Ketterling et al., 1999). The second complication is that the 5′ exon binds a stretch of four uridines of the U5 loop1, making any four nucleotides acceptable at exon positions +2 to +5 as uridine is prone to form isosteric pairs, and there is a mechanism of compensation by U6 snRNA for the poor U5 binding affinity. The safest strategy will be to start with the most conserved exon-end G and its partner U5 39C and swap these nucleotides between the interacting RNAs. We include examples of suitable human mutations for the proof-of-principle laboratory testing, some of which are the continuations of the previous studies introduced and discussed above: Juan-Mateu et al. (2013), Supplementary Figure S5; Vincent et al. (2013), Supplementary Figure S6; Carmel et al. (2004); Scalet et al. (2017)Scalet et al. (2018), and Breuel et al. (2019) (experimental designs for these examples are described in Supplementary Section S5).

#### Intron −3C and U2 snRNA

We suggest that U2 snRNA interacts with the 3′end of the intron: U2 G_31_ = C_–3_ (discussed above). Testing this U2 pair is more straightforward, and any human intron in a minigene construct can do; however, the *Fas/CD95* intron 5 (Corrionero et al., 2011) is an excellent study to follow by swapping the proposed U2 G_31_ = C_–3_ for a double-mutant U2 C_31_ = G_–3_ (Supplementary Figure S7; described in Supplementary Section S5).

### The Incentive: Re-targeted Spliceosomes for Therapeutic Applications

#### Small Nuclear RNAs Targeting Splicing Mutations

Suppression of splicing mutation by matching modifications of U1 snRNA was discovered by Zuang and Weiner in 1986. In 1989, the same authors demonstrated that modifications of U2 snRNA to increase complementary to the target branchpoint site in the human β-globin gene were able to suppress a mutation, which created a cryptic 3′ss. Hwang and Cohen (1996) followed with suppression of the 5′ splice site mutations by compensatory changes in U6 snRNA, which increased complementarity to intron positions +5 to +9. However, while U5 snRNA with modified Loop1 sequence was shown to promote the use of cryptic splice sites, the base-pairing model involved the 5′ intron end, which unfortunately jumbled up experimental planning and conclusions (Cortes et al., 1993). The important outcome of these early studies is that exogenous modified snRNAs, which still have their protein-binding sites unchanged, are recognized by spliceosomal protein components and undergo normal assembly process to form functional ribonucleoproteins (spliceosomal snRNPs).

In spite of these previous studies on specific recognition of pre-mRNA by U2, U5, and U6, today, snRNA therapeutics is largely limited to a U1-based approach (the recent studies include Scalet et al., 2017; Yamazaki et al., 2018; Breuel et al., 2019; Balestra et al., 2020). The efficiency of splicing correction is variable depending on individual mutations and gene context, and a combination of adapted U1 snRNA and antisense oligos that block cryptic slice sites is often used to increase the ratio of normal to aberrant products (recent examples include: Balestra et al., 2015; Breuel et al., 2019; Lee et al., 2019). Encouragingly, modified U1 snRNAs proved to be safe *in vivo* (Lee et al., 2016; Donadon et al., 2019; Balestra et al., 2020), possibly because of the competition with the endogenous wt U1 snRNA and due to nonsense-mediated decay mechanism that removes any jumbled transcripts of off-target genes.

U6 snRNA modification was again attempted by Carmel et al. (2004). The authors used alternatively U1 and U6 adapted to match a substitution of +6T and achieved partial splicing correction of the human *IKBKAP* gene with U1, but not with U6 snRNA. More recently, Schmid et al. (2013) demonstrated that a combination of modified U1 and U6 snRNAs targeting a substitution of +5G in human cells was more effective than U1 alone to rescue the splicing of the *BBS1* gene (Bardet–Biedl Syndrome, a ciliopathy associated with severe vision loss in children).

Scalet et al. (2018) provide experimental evidence of the endogenous U5 supporting modified U1 snRNA to achieve the correction of aberrant splicing of the *FAH* gene (encodes an enzyme of the tyrosine I catabolism; *FAH* deficiency, **H**ereditary **T**yrosinemia type **I**, HTI is associated with cirrhosis and hepatocellular carcinoma). U1 snRNA modified to be fully complementary to the mutant exon/intron boundary CCG/gtga**a**t (the frequent *FAH*c1062+5G>A mutation in intron 12) failed to rescue normal splicing. However, a compensatory effect of a second mutation at the end of exon 12 −2C>A was discovered in a patient with somatic mosaicism and the *FAH* enzyme present in the liver. Expression of a minigene construct bearing both mutations at the exon/intron boundary C**A**G/gtga**a**t in HepG2 cells produced predominantly aberrant splicing products. However, addition of the U1 complementary to CCG/gtga**a**t yielded mostly correct splicing product, although U1 was not complementary to the A_–2_ change. This effect points at the improved U5 pairing in the pre-catalytic complex, which succeeds U1 snRNA binding in the early complex.

The U5 hypothesis provides the binding register for U5 modification to match the target exon junction, and the proposed U2 interaction with the 3′ intron end completes the base-pairing scheme for small nuclear RNAs and pre-mRNA splice sites. We propose that re-targeting all snRNAs, rather than just U1, as is the current practice, will produce a spliceosome with very high affinity to the target intron and splice junction. We can further limit intermixing with endogenous snRNPs by swapping the strands of helix II between the designer U6 and U2 snRNAs. The use of such designer spliceosome with a full set of modified snRNAs will aid both efficiency and precision. Safe transient delivery of small nuclear RNA molecules (human U1, 164 nt; U2, 191 nt; U5, 116 nt; U6, 107 nt), rather than expression constructs, is facilitated by the fact that their maturation involves a cytoplasmic stage, after which they are transported back to the nucleus (Becker et al., 2019).

#### Future Adaptation of snRNAs to Manipulate Regulatory Alternative Splicing Switches

Importantly, applications of snRNA therapeutics are not limited to the correction of individual splicing mutations. Targeting AS switches can be beneficial for patients with common conditions, such as thrombosis. Hemostasis regulation by alternative splicing of coagulation factor V (Vincent et al., 2013, detailed in Supplementary Section S5) is but one example. Such isoform switches are often at the crux of cell fate regulation, providing many clinically important splicing targets. Alternative splicing of the *Fas/CD95* receptor is another example: inclusion of an alternative exon changes a cytoplasmic anti-apoptotic isoform into the transmembrane death receptor (Corrionero et al., 2011). Thus, promoting splicing of a pro-apoptotic isoform by a full set of complementary snRNAs can help to develop tumor suppressor drugs.

#### Future Gene Repair by Reverse Splicing

The future for safe genome engineering eliminating the dangers of bacterial endonucleases will be adapting the human U5 snRNA for correction of genomic mutations by specific reverse splicing. Indeed, human snRNAs form a ribozyme identical to that of mobile introns, which are routinely used for genetic engineering in bacteria (Karberg et al., 2001; Mohr et al., 2013). Reverse splicing was previously demonstrated for the spliceosome *in vitro* (Tseng and Cheng, 2008). It is also known that Group II introns reverse splice into DNA and RNA with comparable efficiency, indicating that 2’O of the target does not affect the reverse splicing process (Griffin et al., 1995). The challenges ahead include increasing the U5 snRNA target recognition specificity and exploring the reverse splicing pathway. However, these are challenges worth taking, as spliceosomes are perfectly placed for endogenous gene therapy tools: highly abundant in transcription loci next to vulnerable single-stranded DNA.

Development of gene repair by specific insertion is needed for the treatment of Duchenne muscular dystrophy (DMD), a sporadic X-linked fatal condition affecting 1:3,500 newborn boys. It is caused predominantly by dystrophin gene deletions that frequently arise within a region of genomic instability, a common fragile site (CFS) in human populations worldwide (Mitsui et al., 2010). The current therapeutic approach uses antisense oligos for exon skipping to restore the reading frame and slow the disease progression. U5 targeting individual deletion site and insertion of missing exons as a fused cassette can offer personalized dystrophin gene repair to cure DMD.

## Materials and Methods

### Creating a Dataset of Base Pairs Between Interacting RNAs *in silico*

We aimed to include approximately 2,000 introns (splice junctions) from human genes ranging from well known in medical genetics practice to genes with experimentally confirmed function and expression (one splice isoform with maximum exons per gene; details and the full list in Supplementary List S1). The sequences of all exons and all introns were downloaded from ensembl.org; we extracted specifically the 11 nt splice junctions (8 nt of the 5′exon end joined to 3 nt of the 3′exon start), intron starts (10 nt), and intron ends (60 nt); introns processed by the minor spliceosome and introns with unusual ends were identified and excluded from the data (excluded introns are detailed in Supplementary Lists S2, S3). Program splice_sites.py calls functions from U5.py (all the code available *via* Git from the U5_hypothesis repository)^4^.

### sKL Divergence

+5G_sKL.py detects +5G substitutions in the major GU_AG introns and sorts exon junctions accordingly into two groups, +5Gsub and +5G (*N_+__5Gsub_* = 445, *N_+__5G_* = 1545), and computes U5 base pair distributions for each group (Figures 4B,C).

The 11 positions of the splice junction were divided into four subsites: 5′exon positions −8 to −6, −5 to −3, −2 to −1 and 3′exon positions +1 to +3. The sKL divergence between the distributions of the base pairs at each of the subsites was calculated as follows:

$$sKL=\sum_{i=1}^{I}\left(p_{i}\log_{2}\frac{p_{i}}{q_{i}}+q_{i}\log_{2}\frac{q_{i}}{p_{i}}\right)$$

where *I* = number of positions number of base pair types, *p*_*i*_ or *q*_*i*_ = probability of a base pair at position *i* in ditribution P or Q, an increment of 0.0001 was added to each probability *p*_*i*_ and *q*_*i*_ to avoid division by 0.

The base line control of 0 divergence is provided by comparing subsets of the larger +5G set to each other, as opposed to the divergence from the smaller +5Gsub. +5G_sKL.py generates 10,000 pairs of random non-overlapping and non-redundant sets of 445 (*N_+__5Gsub_*) splice junctions from the +5G set and calculates sKL divergence for each pair (+5G/+5G, control) as well as sKL divergence between one of the random +5G sets from each pair and the +5Gsub (+5G/+5Gsub). +5G_sKL.py returns the histograms of sKL distributions (Figures 4D–G).

File exG_sKL.py performs analogous operations for the effect of the conserved exon-end G (−1G) on the U6 bp with the start of the intron: initially, it detects exon-end G substitutions and sorts the introns accordingly, exG and exGsub (*N*_*exGsub*_ = 392, *N*_*exG*_ = 1598), and returns bar charts and histograms (Figures 7B–F).

### Bootstrap Procedure

The bootstrap re-samples data with replacement and enables estimates of standard error in properties of the sample that can be used to test the hypotheses of difference (Efron, 1979). Comparing any parameter of two datasets with a bootstrap tests the null hypothesis of no change for this parameter, returning *P*(*H*_0_) for conventional “statistical significance” (McShane et al., 2019). In comparing the +5Gsub and +5G datasets, we bootstrap individually the frequency difference of every base pair type at each position of the splice junction. The algorithm +5G_boots.py performs re-sampling with replacement for both datasets: selecting splice junctions at random allowing for chance reprises and generates a re-sampled set of the same size as the original dataset (*N* = 445 for +5Gsub and *N* = 1,545 for +5G). The program then computes the bootstrap difference (BD) of the frequency of each base pair at each position between the two re-sampled sets. This procedure is iterated 10,000 times to generate a BD distribution for each type of base pair at each position of the splice junction (Supplementary Figure S8). The probability of the null hypothesis *P*(*H*_0_) of no difference between the +5Gsub and +5G is returned for each individual frequency. The standard definition for *P*(*H*_0_) for the bootstrap hypothesis testing is the proportion of the smaller part of the BD distribution lying beyond 0 line (Figure 5). The program +5G_boots.py also summarizes all the histograms as three sets of violin plots (Figures 5A–C). The same program further deals separately with 5′ exon position −3 shared by U1 and U5 binding sites, but with different nucleotides required for Watson–Crick pairs with U1 and U5 snRNA. +5G_boots.py returns the stacked bar chart of the nucleotide frequencies (rather than base pair-type frequency) for this position in +5Gsub and +5G datasets, bootstraps these frequencies, and computes *P*(*H*_0_) (Figure 6).

Script exG_boots.py compares exGsub and exG datasets (*N-_1Gsub_* = 392, *N*_–1G_ = 1598) using the same algorithm to generate the BD distribution for each individual bp frequency at each position of the U6/intron interaction and returns violin plots and *P*-values (Figures 5D–F).

The file −3C_boots.py first detects substitutions of the conserved −3C in the dataset of introns processed by the major spliceosome, including GC(A)_AG introns along with GU_AG, sorts the exon junctions into two groups, −3Csub and −3C (*N_–3Csub_* = 792, *N-_3C_* = 1211), and computes the U5/exons bp frequency (Figures 8B,C). The program then follows the bootstrap procedure algorithm as described above and returns violin plots (Figures 8D–F).

### Bonferroni Correction for the Multiple Significance Tests (Dunn’s Method)

The danger of testing multiple hypotheses is that some “significant” result may occur by chance alone (Bland and Altman, 1995). The simple Bonferroni correction or Dunn’s α-splitting (Lee and Lee, 2018) implies that the widely used threshold of statistical significance α = 0.05 must be divided by the number of tests *m* performed on each dataset.

$$\alpha^{\prime }=\frac{\alpha }{m}=\frac{0.05}{m}$$

Accordingly, the corrected *p*-value thresholds for significant changes for base pair frequency tests here are as follows: For the +5G/+5Gsub experiment, *m* = 31, α′ = 0.0016; for the −1G/−1Gsub experiment, *m* = 17, α′ = 0.0029; for the −3C/−3Csub experiment, *m* = 28, α′ = 0.0018; and considering all tests in this study, *m* = 76, α′ = 0.0007. The *p*-values in Figures 5, 8 are marked with triple asterisks or double asterisks if below their respective thresholds for all tests or individual experiments.

Dunn’s application of Bonferroni correction is a stringent method, which is more likely to reject a true positive (Type II error) than to accept a false positive (Type I error) (Lee and Lee, 2018). The application of this method is justified if the outcomes of the hypothesis tests are not related. The comparisons here are independent for the positions of the sites, but strongly correlated for base pair types at each individual position, e.g., an increase in Watson–Crick pairs means the decrease in isosteric pairs if there are only two pair types or the decrease in either non-isosteric or isosteric pairs (or both) if there are three pair types at any given position. Therefore, we can adjust *m* for the correlated tests (Shi et al., 2012):

$$m^{\prime }=\left(m+1\right)-\left[1+\left(m-1\right)\times R\right]$$

where *R* is the interclass correlation correction such as 0 ≤ *R* ≤ 1.

In simple terms, positions with only two pair types account for two perfectly correlated tests, so *R = 1*, and these two tests will count as one. For tests with three types, we can approximate *R* ≈ 0.5 by splitting the correlation between them and *R* ≈ 0.33 when there are four tests. Following this procedure, for the +5G/+5Gsub experiment, *m*′ = 20, α′ = 0.0025; for the −1G/−1Gsub experiment, *m*′ = 11, α′ = 0.0045; and for the −3C/−3Csub experiment, *m*′ = 17, α′ = 0.0029. *P*-values below their respective experiment thresholds accounting for correlated tests are marked with a single asterisk in Figures 5, 8.

Here, we remark on the current debate on “statistical significance” among the statisticians: McShane et al. (2019) point out that the null-hypothesis significance testing—and generally accepted *p*-value threshold of 0.05—is a misleading paradigm for research and instead *P*(*H*_0_) should not be prioritized over other factors, such as plausibility of mechanism and related prior evidence (in this case, genomic conservation and mutation data).

## Data Availability Statement

The original contributions presented in the study are included in the article/Supplementary Material, further inquiries can be directed to the corresponding author/s.

## Author Contributions

OA-I performed the initial sequence alignments of U5 with human exon junctions and *Ll.*LtrB DId3 with bacterial retrotransposition sites, designed the analyses of the relative distributions of base pairs by position in human exon and intron interactions, wrote the Python code and performed the analysis. OA-I is responsible for the new U5 interactions model and comparison with the current CryoEM model. OA-I linked human mutation data to the new U5 model, wrote the codes in Python and R and performed the tests. OA-I wrote the manuscript and preparation the figures. AP directed the choice of the journal and preparation for the experimental verification by exploring the role of U5 in the alternative splicing of the human F5 gene. AP revised and corrected the manuscript and figures. Both authors contributed to the article and approved the submitted version.

## Conflict of Interest

The authors declare that the research was conducted in the absence of any commercial or financial relationships that could be construed as a potential conflict of interest.

## Footnotes

1 http://webapps2.ucalgary.ca/~groupii/

2 https://genesilico.pl/SimRNAweb

3 http://ndbserver.rutgers.edu/ndbmodule/services/BPCatalog/bpCatalog.html

4 https://github.com/oartem01/U5_hypothesis
