# Supplementary material for: U5 snRNA Interactions With Exons Ensure Splicing Precision
Source: Front Genet. 2021 Jul 2;12:676971. doi: 10.3389/fgene.2021.676971 (PMC8283771; doi:10.3389/fgene.2021.676971)
Supplement: Supplementary file 1 [file Data_Sheet_1.pdf]

SUPPLEMENTARY MATERIAL

Contents

Section S1: Tables	page
**Table S1.** Spliceosomal RNA network at successive stages of splicing, defined as distinct RNP complexes	3
**Table S2.** Diverse views of the U5 snRNA Loop1 interactions with exons were obtained by crosslinking experiments and Cryo electron microscopy by different research groups.	
**S2.A.** Budding yeast *(Saccharomyces cerevisiae)* spliceosome	4
**S2.B.** Human spliceosome with adenovirus pre-mRNA substrates	5
**Table S3**. Spliceosomal RNA-RNA interactions confirmed by genetic and biochemical studies are not always captured by Cryo electron microscopy	
**S3.1** Budding yeast *(Saccharomyces cerevisiae)* spliceosome	6
**S3.2** Human spliceosome built on the miniature adenovirus pre-mRNA substrate (MINX)	7
**Table S4 (See Section S4)**	

Section S2: Figures	
**Figure S1|** Diverse group II introns recognise their exons mainly by Watson-Crick base pairing with the Id3 loop in a common asymmetrical fashion.	8
**Figure S2|** 'Legal' C--U pairs in the U5 interactions prompted by the initial U1 selection	9
**Figure S3|** Quick guide to sugar-phosphate backbone orientation relative to the plane of a base	10
**Figure S4|** Canonical Watson-Crick pairs with antiparallel strands	10
**Figure S5** (see **Section S5**)	42
**Figure S6** (see **Section S5**)	43
**Figure S7** (see **Section S5**)	44
**Figure S8** (see **Section S4**)	49-54
**Figure S9** (see **Section S4**)	55-57
**Figure S10** (see **Section S4**)	58-63
**Figure S11 (**see **Section S4)**	24
**Figure S12 (**see **Section S4)**	15
**Figure S13 (**see **Section S4)**	16

Section S3: Comments to Figures	
**Comment S1** (to **Figure 12**) The odd feature of the of U12 interaction with the U6atac catalytic triad in the human minor spliceosome: a bulge or a Watson-Crick-like mimic pair?	11
**Comment S2** (to **Figure 14**) The paradox of the stalled transition in the absence of the Prp16 helicase	11

Section S4: Results and Methods	
**List S1.** List of human genes included in the analysis by sKL divergence and Bootstrap	12-14
**Description of 132 human genes included in the analysis**	15
**Figure S12|** Functional classification of human genes included in the analysis	15
**Figure S13|** Characterisation of introns in human genes included in the analysis	16
The *ensembl* isoform number is featured in the sequence file names	17-19
**Unusual introns** (identification and isolation)	20
**List S2.** Minor introns	21
**List S3.** Major introns with substitutions of +2U: GC(A)_AG introns	21
**List S4.** Introns missing both conserved exon-end -1G and intron +5G	22
**Figure S8|** Histograms of bootstrap difference for U5 bp types at each position of the exon junctions between +5Gsub and +5G datasets (Violinplots of the same **Figure 6A-C**)	49-54
**Figure S9|** Histograms of bootstrap difference for U6 bp types at the start of intron position +5 to +10 between -1Gsub and -1G datasets (Violinplots of the same **Figure 6D-F**)	55-57
**Figure S10|** Histograms of bootstrap difference for U5 bp types at each position of the splice junction between -3Csub and -3C datasets (Violinplots of the same **Figure 9D-F**)	58-63
**Human mutation data explained by the U5 hypothesis**	23
**List S5.** Mutations of exon-start guanine: $G_{+1} \rightarrow$ *T* or $G_{+1} \rightarrow$ *A* (Fu et al., 2011)	23
**Figure S11|** Inclusion of exon affected by +1G mutations is influenced by multiple *cis* factors	24
**Table S4. F**actors that promote efficient exon inclusion (PSI 81-100%) in spite of +1G mutations	25
Kruskal-Wallis rank sum test and Welch's ANOVA (t-test) for **+2C/+3G**	26
Welch's ANOVA and Kruskal-Wallis rank sum test for **substitute A or T**	30
Welch's ANOVA and Kruskal-Wallis rank sum test for **-3C**	33

Boxplots for PSI dependent on +2C/+3G, substitute A or T and -3C 36
Spearman's correlation for PPS length 37
Spearman's correlation for branchpoint matches 38
Scatterplots for PSI dependent on PPS length and branchpoint matches 39

Section S5: Future work: Molecular cell biology testing of the U5 hypothesis
Human mutations suitable for the proof-of-principle laboratory testing 40
Figure S5| Study design for the correction of the dystrophin gene splicing mutation c9563+5G>C in intron 65 from a Becker muscular dystrophy patient reported by Juan-Mateu et al., 2013. 42
Figure S6| Study design targeting the alternative intron (pseudo-intron) splicing of coagulation F5. 43
Figure S7| Testing U2 snRNA interaction with the end of the intron (the proposed U2 G_{31}=C_{-3} pair) following the study of Corrionero et al., 2011 on Fas/CD95 intron 5. 44

Supplementary References 45-48

Section S1: Tables

Table S1. Spliceosomal RNA network at successive stages of splicing, defined as distinct RNP complexes

Spicing stage	**RNP complex**[1]	Proteins in action	**RNA network**
Initial 5'splice site (5'ss) selection	**E** Early complex	SF1 binds BP	**5'ss/U1** - binds across exon-intron boundary
Branch point (BP) and 3'splice site (3'ss) recognition	**A** Pre-spliceosome	**$U2AF^{65}$ binds the PPT and curves it into a sharp loop**[2] $U2AF^{35}$ binds 3'ss - the intron end AG	**BP/U2** - **BP helix bridges over the PPT bringing the bulged A to the fixed 4nt distance from the intron end**[3]
Spliceosome Assembly	**Pre-B**		U4/U6•U5 tri-snRNP joins in, all snRNPs are assembled together
	**B** Pre-catalytic spliceosome	Prp28 displaces U1C and initiates 5'ss/U1 unwinding	U1 quits 5'ss<br>**U2, U5 and U6 cooperate in specific sequence recognition to achieve splicing fidelity**<br>**5' intron end pairs with U6-$A_{39}\Psi_{40}A_{41}C_{42}\boxed{A^{m6}_{43}G_{44}}A_{45}$ (bases forming conserved non-WC pairs are boxed)**<br>**3' and 5' exons align on the U5-Loop 1.**<br>**Intron termini form a non-canonical pair.**<br>**Pre-mRNA is completely arranged for the catalysis**
Re-arrangement of the catalytic core	**Bact** Activated spliceosome	**Brr2 unwinds U4/U6 stem I helix translocating 3' to 5' along U4**<br>NTC/NTR join in	U4 quits U6<br>**Formation of the ribozyme catalytic core: U2/U6 triple helix and U6 dinucleotide bulge coordinate the two catalytic Mg^{2+}ions.**
Catalytic activation	**B*** Catalytically activated spliceosome	Prp2 promotes binding of the step 1 factors Cwc25, Yju2, and Isy1	
Branching reaction	**C** Catalytic step 1 spliceosome		Intermediates: 5' exon cut off the intron, 3' exon with the intron lariat<br>**Both exons stay bound to the U5 Loop 1**
Transition between the two steps of splicing	**C*** Catalytic step 2 spliceosome	Prp16 promotes the release of the step 1 factors and binding of Prp22 and the step 2 factors Prp18 and Slu7	**Branching triggers a rotation of the BP helix on its axis. The intron termini pair pulls on the 3'exon. U5 loop 1 facilitates the translocation of the 3'exon due to the relaxation caused by the snipping of the intron bond from the 5'exon. The G_{-1} and 3' exon position for the step 2 catalysis.**
Exon ligation	**P** Post-splicing spliceosome		Final products:<br>Ligated exons and the intron lariat
Release of the ligated exons	**ILS** Intron-lariat spliceosome	Prp22 promotes the release of step 2 protein factors	Disassociation of the ligated exons
Spliceosome disassembly	**U6, U5, U2 snRNPs**	Prp43	Release of the intron lariat

[1] Wahl et al. 2009 and 2015, Scheres and Nagai 2017, Bertram et al. 2017[b]; [2] Sickmier et al. 2006, Chen et al. 2010, Kent et al. 2003; [3] based on Corrionero et al. 2011 and Brock et al. 2008; **in blue**: see Discussion.

Table S2. Diverse views of the U5 snRNA Loop1 interactions with exons were obtained by crosslinking experiments and Cryo electron microscopy by different research groups.

S2.A. Budding yeast *(Saccharomyces cerevisiae)* spliceosome

**Crosslinking with 4-thioU**<br>Newman et al.1995;<br>Teigelkamp et al. 1995	**Luhrmann CryoEM**<br>Rauhut et al. 2016	**Nagai CryoEM**<br>Galej et al. 2016;<br>Fica et al. 2017;<br>Scheres and Nagai, 2017, Wilkinson et al, 2017	**Shi CryoEM**<br>Wan et al. 2016[a,b], 2019;<br>Yan et al. 2016, 2017;<br>Bai et al. 2017
5' exon<br>***CYH2* 5'ex 4-thioU**<br>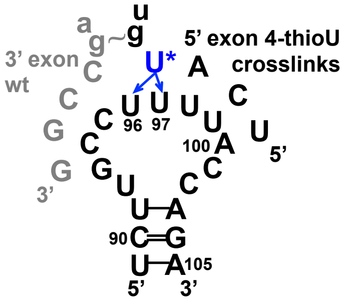<br><br>Detected before and after the 1st step (**Bact** and **C** complexes[1]).<br>**4-thioU replaced a G** (see wt 5' exon below).<br>The non-canonical intron termini pair is cited in this paper after Parker and Siliciano 1993, Chanfreau et al. 1994, Scadden and Smith 1995.	5' exon<br>***ACT1***<br>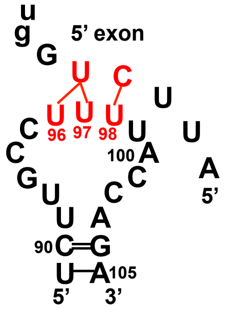<br><br>**Bact** complex	5' exon<br>***UBC4***<br>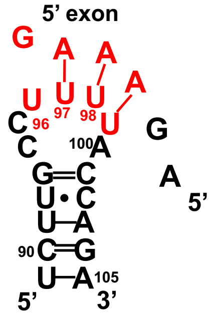<br><br>**C** complex<br>5' exon binding is the same in **C*** and presumed the same in **Bact**	5' exon<br>***TUB3***<br>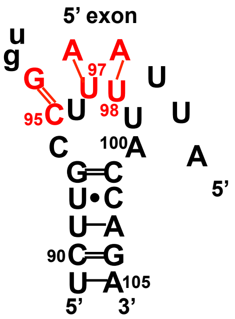<br><br>**B** complex<br>***UBC4***<br>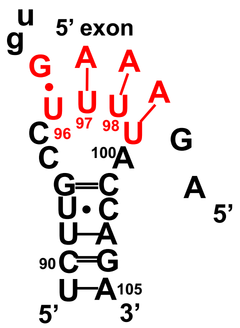<br><br>**Bact** complex<br>5' exon binding register is the same in **B***, **C** and **C***
3' exon<br>***CYH2* 3'ex 4-thioU**<br>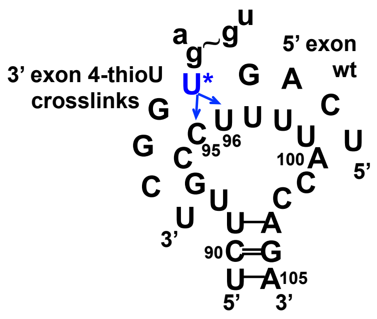<br><br>Detected in the lariat intermediate (**C** complex). 3'		3' exon<br>***UBC4***<br>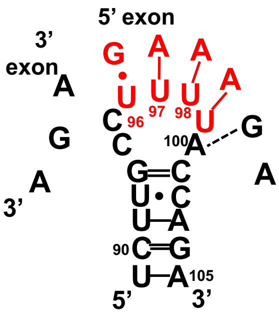<br><br>**P** complex<br>3' exon base-pairing not defined	3' exon<br>***TUB3***<br>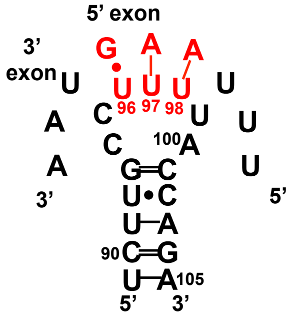<br><br>**P** complex

S2.B. Human spliceosome with adenovirus pre-mRNA substrates[1]

Crosslinking with 4-thioU<br>Sontheimer and Steitz 1993	Luhrmann CryoEM<br>Bertram et al. 2017a,b	Shi and **Nagai** CryoEM<br>Zhan et al., 2018[a,b]<br>Zhang et al. 2017, 2018, 2019; **Fica et al., 2019**

5' exon

***Ad5-1* 5'ex 4-thioU**

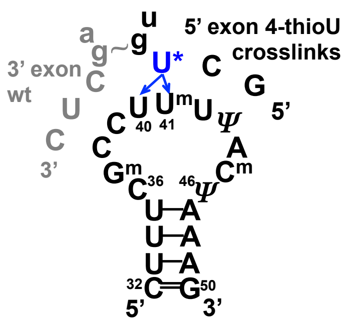

Detected before and after the 1st step (**Bact** and **C** complexes[2]). **4-thioU replaced a G** (the subultimate G was also changed to C to avoid a GU, see wt 5' exon below). The non-canonical intron termini pair is cited in this paper after Parker and Siliciano 1993.

5' exon

MINX

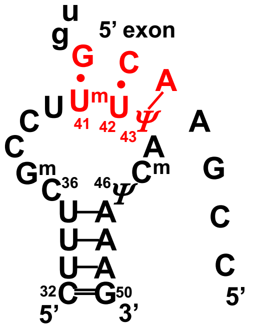

B complex

MINX

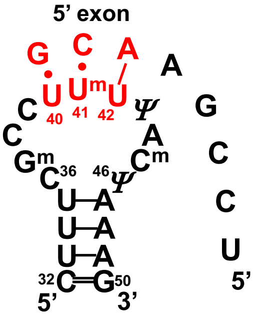

C* complex

5' exon

MINX

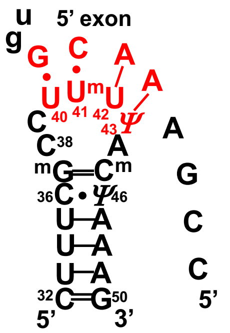

B and Bact complex

MINX

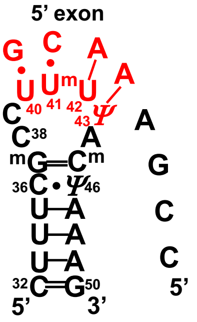

C* complex
5' exon binding is presumed the same in **C**

3' exon

***Ad3+1* 3'ex 4-thioU**

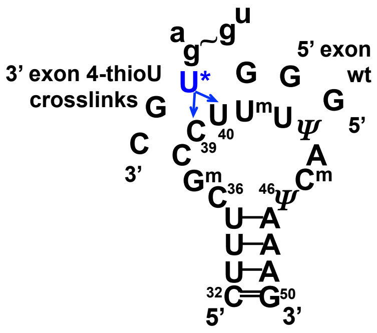

Detected in the lariat intermediate (**C** complex). 3' exon sequence is not the wt (see above).

MINX

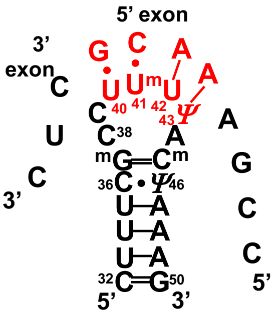

P complex
No base-pairing is defined for 3' exon and U5 Loop1

[1] The pre-mRNA substrates are the derivatives of the Adenovirus-2 major late transcription unit. *Ad5-1* and *Ad3+1* are 4-thioU 5' and 3'ss modifications of the *Adeno* 'standard splicing substrate' (409nt): late exons 1 and 2 separated by the intron 1 with most of it deleted Δ198-982 (Sontheimer and Steitz 1993; Wyatt et al.1992; Solnick 1985).
MINX - **Mini**ature pre-mRNA substrate (220nt) contains 3' and 5' parts of the late exon 2 separated by a small composite intron consisting of the start of intron 2 (5'ss) followed by a short stretch of the plasmid vector and the end of intron 1 with the branch point and 3'ss (Zillmann et al. 1988; Padgett et al. 1984).
[2] See **Table S1** for definitions of the stages of splicing as distinct RNP complexes

Table S3. Spliceosomal RNA-RNA interactions confirmed by genetic and biochemical studies are not always captured by Cryo electron microscopy. Different stages of splicing are defined as distinct RNP complexes: **B** to **ILS** (see **Table S1**). RNA bp are defined by Westhof geometry (Leontis et al. 2002)

S3.1 Budding yeast *(Saccharomyces cerevisiae*[1]*)* spliceosome

**Laboratory**		**R. Lührmann**	**K. Nagai**	**Y. Shi**
**Spliceosomal complexes**<br>References<br>Methods used to stall at a particular stage of splicing *in vitro* or enrich for particular complexes by affinity purification of the isolated total spliceosomes[4]	**B**		Plaschka et al., 2017 Low ATP	Wan et al. 2016[a]<br>Prp6 tagged
	**Bact**	Rauhut et al. 2016 prp2-1 heat inactivation		Yan et al. 2016<br>Cef1 affinity[3], 2D/3D image classification
	**B*, C**		Galej et al. 2016<br>3'ss mutation	Wan et al. 2019, 2016[b]<br>Cef1 afinity[3],<br>B*: Bact$^{\Delta Prp2}$ + Prp2 and Spp2
	**C***		Fica et al. 2017<br>3'ss mutation	Yan et al. 2017<br>Cef1 affinity[3]
	**P**		Wilkinson et al, 2017 Prp22^{S635A}	Bai et al. 2017<br>Cef1[3], Prp22^{K512A}
	**ILS**			Wan et al. 2017<br>Cef1[3], Yju2 tagged
**Pre-mRNA substrate**		*ACT1:* ex1-end, in1, ex2-start, 3x MS2 aptamers[2]	*UBC4*<br>**C:** uacAC<br>**C*:** ua(^{d}g)AG<br>**P, B**: uagAG (wt)<br>3' or 5' 2-3x MS2[2]	Endogenous pre-mRNA<br>**B*:** *UBC4, ACT1* both 5' 3x MS2[2]
**Intron termini pair presence and configuration**<br>G_{+1}–○–G_{-1}<br>Confirmed by mutation analysis, interchangeable for a double mutant<br>A_{+1}–○–C_{-1}<br>nearly isosteric in this configuration (Parker and Siliciano 1993, Chanfreau et al. 1994)		Absent in **Bact**	Absent in **B**;<br>**C** and **C***: cannot form due to G_{-1} mutations;<br>G_{+1}●–■G_{-1} in **P**<br>*parallel strands, however, this configuration is impossible for A-C and A-A pairs*	Absent in **B-**<br>**-Bact-B*-C-C*-ILS**;<br>G_{+1}●–■G_{-1} in **P**
**5' intron end interaction with U6**<br>U_{+2}—A_{51}<br>Detected by cross-linking experiments in **Bact** (stalled by prp2-1 heat inactivation) and **C** complexes (Kim and Abelson 1996, Fabrizio and Abelson 1990)<br>A_{+3}□–▷G_{50}<br>Confirmed by mutation analysis (*RPL30* gene), interchangeable with<br>A_{+4}□–▷A_{49}<br>isosteric in this configuration (Konarska et al. 2006)		U_{+2} is unpaired in **Bact**<br><br>A_{+3} is unpaired in **Bact**	Presumed in **Bact** (Plaschka – 2017); absent in **C**;<br>U_{+2}○–□A_{51} in **C***<br><br>Presumed in **Bact** (Plaschka – 2017);<br>A_{+3}□–▷G_{50} in **C**;<br>absent in **C***	U_{+2} is unpaired in **B-Bact-B*-C-C*-P-ILS**<br><br>A_{+3} is unpaired in **B-Bact-B*-C-C*-P-ILS**

[1] The structure for *Schizosaccharomyces pombe* **ILS** complex is reported by Yan et al., 2015
[2] **MS2:** RNA loops added to the 3' or 5' end or inside the intron to bind an aptamer coat fusion protein MS2-MBP for subsequent affinity purification.
[3] Cef1 protein is a component of the NTC complex, which joins the spliceosome when the catalytic core is formed, stays part of it during both steps of catalysis and remains with the intron lariat after the release of the ligated exons. Therefore, isolation by Cef1 affinity renders a mixture of **Bact, B*, C, C*, P** and **ILS** complexes.
[4] To facilitate isolation of complexes with U4/U6.U5 tri-snRNP Wan et al. 2016[a] used yeast expressing tagged Prp6, a protein component of the U5 snRNP. To enrich for spliceosomes stalled before exon ligation Bai and co-workers added Prp22-K512A ATPase defective mutant to the yeast cultures. To eliminate the 1st step spliceosomes from the isolated mixture Wan et al. 2016[b] used yeast expressing tagged 1st step factor Yju2.

S3.2 Human spliceosome built on the miniature adenovirus pre-mRNA substrate (MINX[1])

Laboratory		R. Lührmann	K.Nagai	Y. Shi
**Spliceosomal complexes**<br>References<br>Methods used to stall at a particular stage of splicing *in vitro*	**Pre-B**<br>**B**	Bertram et al. 2017[b] Low Mg^{2+}, *Brr2* stalled	Charenton et al., 2019 Prp28 mut DEAD → AAAD	Zhan et al., 2018[b] Phosphatase inhibitor
	**Bact**	Haselbach et al., 2018 Pre-mRNA 3' deletion		Zhang et al. 2018 Pre-mRNA 3' deleletion
	**C**			Zhan et al. 2018[a] BN82685 inhibition[3]
	**C***	Bertram et al. 2017[a]<br>Low pH 6.4[2]		Zhang et al. 2017 3'ss mutation
	**P**		Fica et al., 2019 Prp22 mutants S717A, K594A	Zhang et al., 2019 Prp43 mut T429A
	**ILS**			Zhang et al., 2019 Prp43 mut T429A
**Pre-mRNA substrate**		**B** and **C*: MINX** uniformly $[^{32}P]$-labeled<br>**Bact: AdML[1]** derivative **PM5**-10 3' part deletion starting 10nt after BP, 5' 3x MS2	**Pre-B** complex: **AdML** pre-mRNA, 3' 3x MS2<br>**P** complex: **MINX** with 3'end 3x MS2 loops	**Pre-B**: mod **MINX,** 3' 2x MS2<br>**Bact**: **MINX-15**: 3' del 19nt after BP;<br>**C**: **MINX**<br>**C***: **MINX-GG**, intron 3' ag to gg<br>**P, ILS**: mod **MINX,** 2x MS2 in intron
**Intron termini pair presence and configuration**<br>**G-○-G** (G_{+1}, G_{-1})<br>Confirmed by mutation analysis, interchangeable for double mutants<br>**A-○-C** (A_{+1}, C_{-1}) **A-○-A** (A_{+1}, A_{-1})<br>nearly isosteric in this configuration (Scadden and Smith 1995)		Cannot form in **Bact** due to the deletion of the 3' part of the intron<br><br>Absent in **B** and **C***	Absent in **Pre-B**;<br>**G●-■G** (G_{+1}, G_{-1}) in **P**<br>*parallel strands, however, this configuration is impossible for A-C and A-A pairs*	Absent in **B**; cannot form in **Bact** due to the deletion of the 3' part of the intron; absent in **C** and **C***;<br>**G●-■G** (G_{+1}, G_{-1}) in **P** and presumably **ILS**
**5' intron end interaction with U6**<br>**U—A** (U_{+2}, A_{45})<br>Detected by cross-linking experiments in **C** complex (lariat intermediate) and **P** complex (lariat intron product). (Sontheimer and Steitz 1993)<br><br>**A□-▷G** (A_{+3}, G_{44}) **A□-▷A^{m6}** (A_{+4}, A_{43})<br>These pairs can be inferred from the mutation analysis in yeast (*RPL30* gene by Konarska et al. 2006) as both $\mathbf{A_{+3}}$ and $\mathbf{A_{+4}}$ are conserved in humans (78 and 68% of the human dystrophin gene introns respectively). The presence of at least one of these adenines supports the formation of the key non-WC pair.		$\mathbf{U_{+2}}$ is unpaired in **B, Bact** and **C***<br><br>$\mathbf{A_{+3}}$ and $\mathbf{A_{+4}}$ are aligned with U6 $\mathbf{G_{44}}$ and $\mathbf{A^{m6}_{43}}$, in **B** and **C***, base-pairing is not specified, in **Bact** PM5-10 substrate has $\mathbf{U_{+4}}$ and WC-pair with $\mathbf{A^{m6}_{43}}$	**U○-□A** (U_{+2}, A_{45}) in **P**<br><br>$\mathbf{A_{+3}}$ and $\mathbf{A_{+4}}$ are paired with U6 $\mathbf{G_{44}}$ and $\mathbf{A^{m6}_{43}}$ in **B** and **P** complex, base-pairing configuration is not specified	$\mathbf{U_{+2}}$ is unpaired in **B, Bact, C** and **C*;**<br>**U○-□A** (U_{+2}, A_{45}) in **P** & presumably **ILS**<br><br>$\mathbf{A_{+3}}$ and $\mathbf{A_{+4}}$ are aligned with U6 $\mathbf{G_{44}}$ and $\mathbf{A^{m6}_{43}}$, in **B,** base-pairing is not specified; $\mathbf{A_{+3}}$ is unpaired in **Bact, C** and **C*,** $\mathbf{A_{+4}}$ is near $\mathbf{A^{m6}_{43}}$ in **Bact, C** and **C***, but base-pairing is not specified.

[1] **MINX** – see **Table S2B** footnote [2]**; AdML:** Ad major late (Anderson and Moor, 1997), **PM5** (Bessonov et al., 2008), **MS2:** RNA loops added to the 3' or 5' end or inside the intron to bind an aptamer coat protein for subsequent affinity purification.
[2] *In vitro* splicing proceeds normally at pH7.9 in the presence of HeLa nuclear extracts
[3] BN82685 is a specific inhibitor of pre-mRNA splicing stalling the reaction at or before the 1st step of splicing (C-complex)

Section S2: Figures

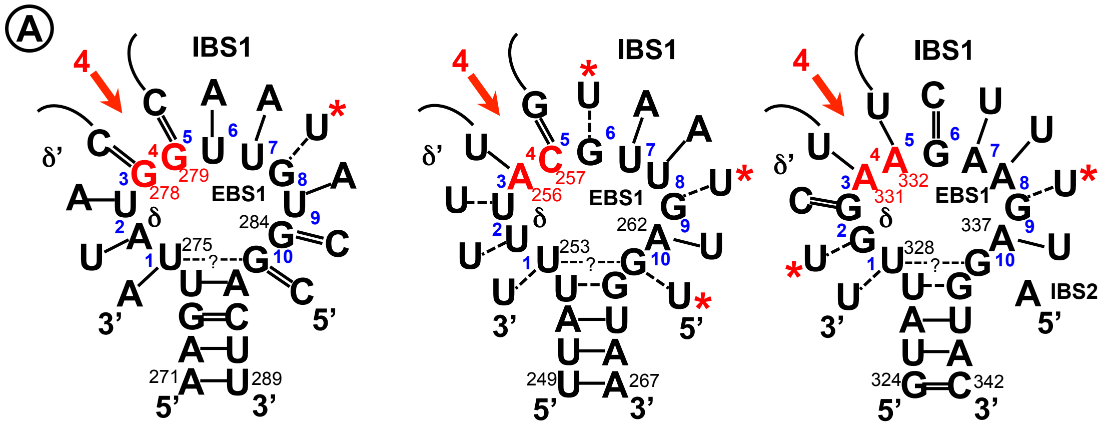

Ll.LtrB (L.l.I1)
intron of the *ltrB* gene encoding a relaxase found in the conjugative elements of *Lactococcus lactis*

aI1 (cox1.I1) **aI2** (cox1.I2)
introns of the mitochondrial gene *cox1* encoding cytochrome c oxidase subunit I in *Saccharomyces cerevisiae*

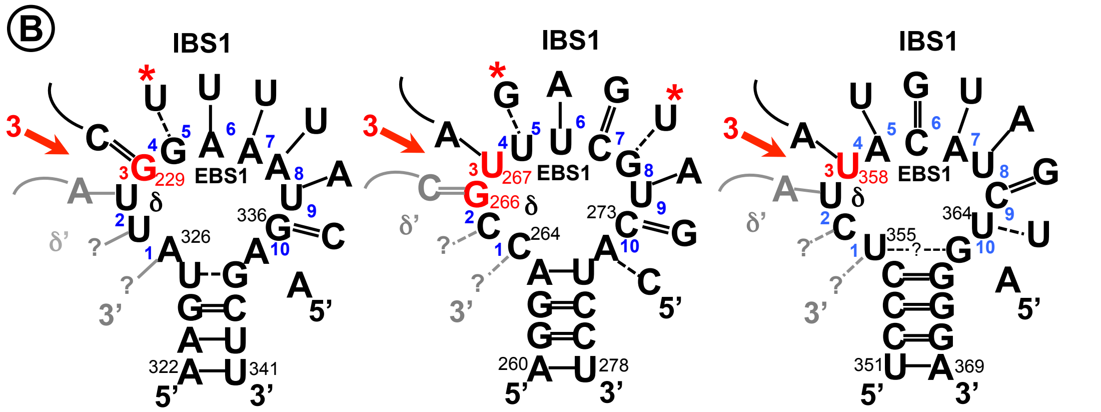

ai5γ
intron of the mitochondrial gene *cox1* encoding cytochrome *c* oxidase subunit I in *Saccharomyces cerevisiae*

RmInt1 (Sr.me.I1)
intron found within the insertion sequence IS *Rm2011-2* in bacterium *Sinorhizobium meliloti,* a nitrogen-fixing symbiont of alfalfa *(Medicago sativa)*

Avi.groEL (A.v.I1)
intron of an essential housekeeping gene required for protein folding, chaperonin *groEL*, found in *Azotobacter vinelandii*

Figure S1| Diverse group II introns recognise their exons mainly by Watson-Crick base pairing with the Id3 loop in a common asymmetrical fashion. The arows indicate positions for the exon junction 3 or 4 referring to all 'possible' positions numbered in blue.
A. Id3 loops of these **Group IIA** introns bind both exons: 6-7nt at the end of the 5' exon and 1-4nt at the start of the 3' exon (Plante and Cousineau 2006, Zhong and Lambowitz 2003, Ichiyanagi et al. 2002, Eskes et al. 1997, 2000).
IBS1, Intron Binding Sequence 1 is the end of the 5'exon, δ' sequence is start of the 3'exon. EBS1, Exon Binding Sequence 1 and δ sequence of the Id3 loops bind the 5' and 3' exons respectively.
B. Id3 loop of **Group IIB** introns typically interacts with 7-8nt of the 5' exon and secures the 3' exon via a coordination loop and a further EBS3 – IBS3 interaction (Somarowthu et al. 2014, Ferat et al. 2003, Chillón et al. 2014). EBS3 of the coordination loop binds IBS3 of the 3' exon. δ' sequence of the coordination loop is shown in grey.

* G-U and U-G pairs are indicated with red asterisks

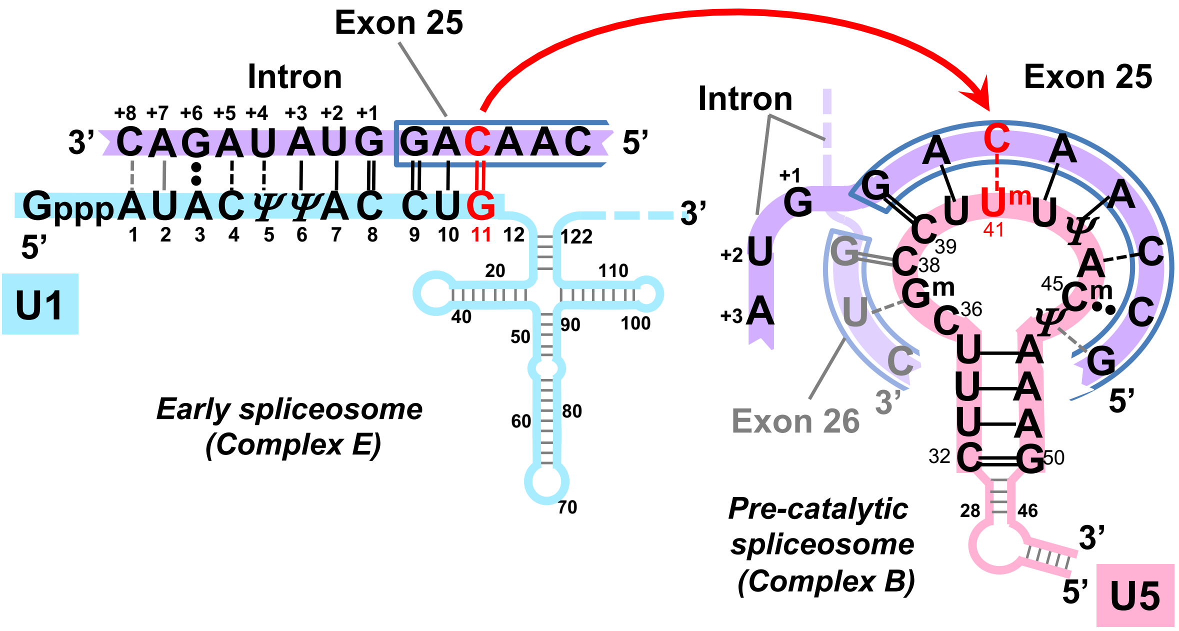

Figure S2| 'Legal' C--U pairs in the U5 interactions prompted by the initial U1 selection (Human dystrophin exon/intron 25 as an example).
Base modifications as in **Figure 1** caption.

Sugar-phosphate backbone orientation (+/o) relative to the plane of a base

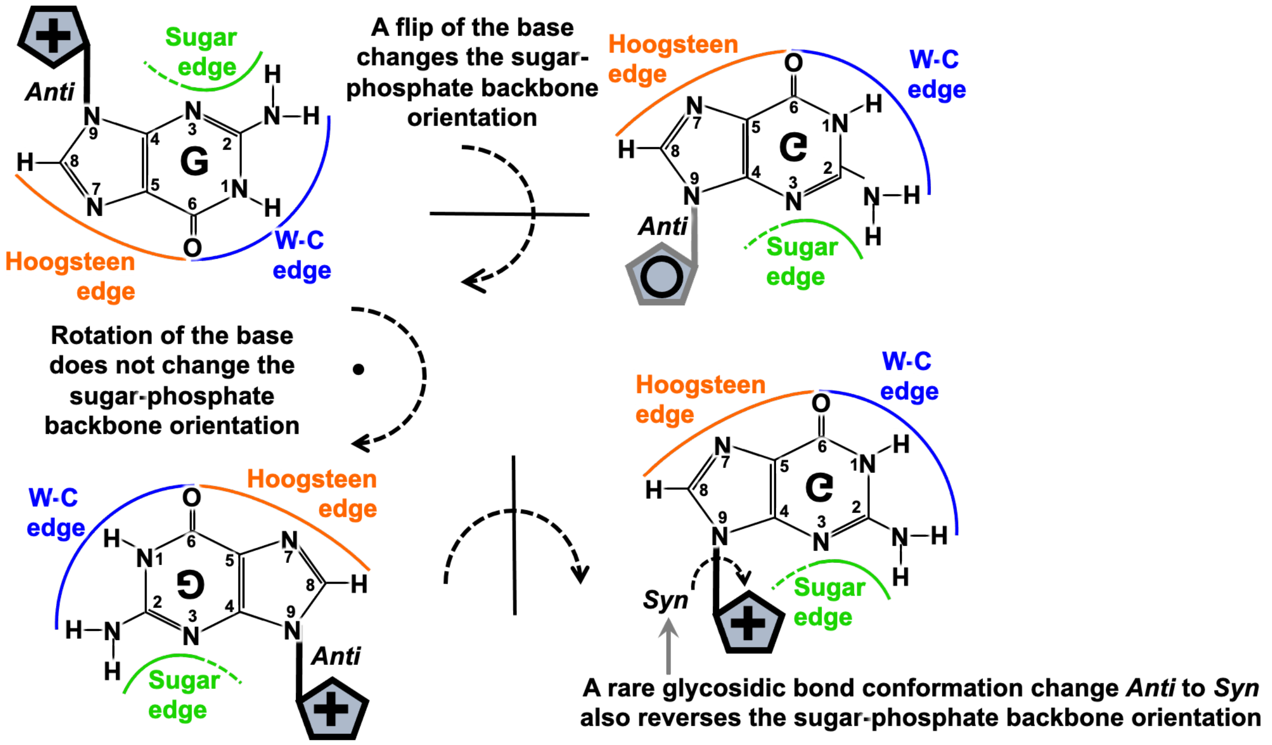

Figure S3| Quick guide to sugar-phosphate backbone orientation relative to the plane of a base (explanatory schematics to accompany Figure 13). Ribose is located on the perpendicular plane and is shown as a schematic blue pentagon with **+** and **o** indicating the opposite directions of the sugar-phosphate backbone.

Canonical Watson-Crick, antiparallel strands, 1st Westhof geometric family

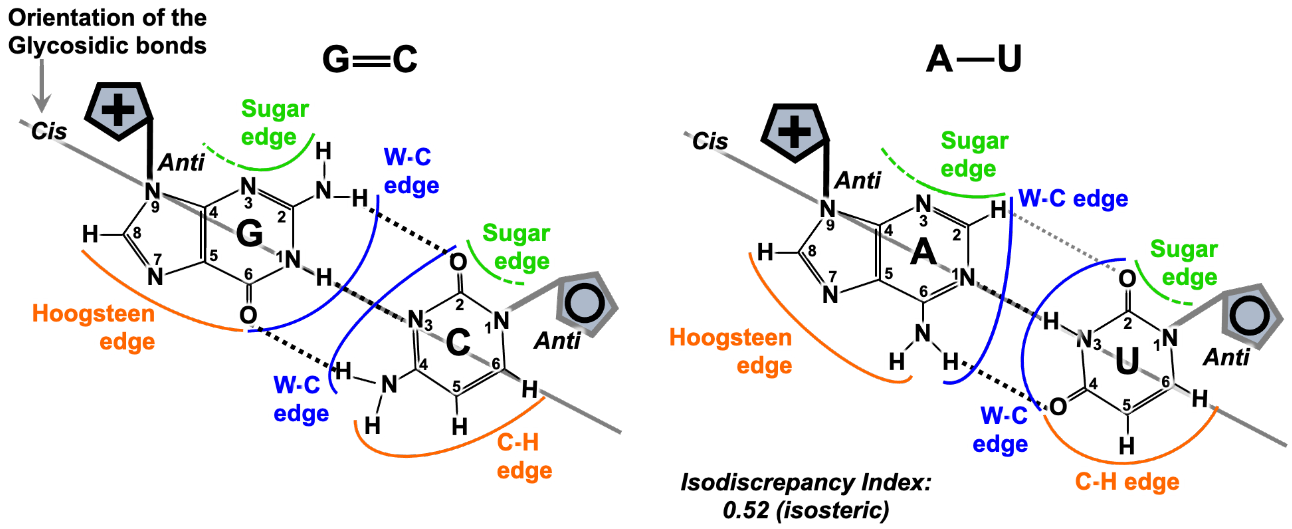

Figure S4| Canonical Watson-Crick pairs with antiparallel strands. Isodiscrepancy index is a numerical measure of geometric similarity (isostericity) of base pairs (on-line RNA base pair catalogue http://ndbserver.rutgers.edu/ndbmodule/services/BPCatalog/bpCatalog.html).

Section S3: Comments to Figures

Comment S1 (to Figure 12)
The odd feature of the of U12 interaction with the U6atac catalytic triad in the human minor spliceosome: a bulge or a Watson-Crick-like mimic pair?

According to the review of Turunen et al. 2013, which faithfully reproduces the original Tarn and Steitz 1996[b], U12 C_4 is bulging out of the helix U12 forms with the minor spliceosome catalytic triad U6atac $A_{26}G_{27}C_{28}$ (compare Helix Ib in **Figure 10C** and **10A**). This is a surprising feature, as we assume the same spatial organisation of the key catalytic triple helix in the paralogous spliceosomes. In the minor spliceosome of *Arabidopsis* there is no bulging inside Helix Ib (Shukla and Padget 1999), although the binding register is currently disputed (Ciavarella et al., 2020).
There is a possibility, hitherto unexplored, that bulging inside Helix Ib can be avoided by formation of a Watson-Crick-like pair of A_{26}--C_5 followed by G_{27}=C_4 (residue numbers as in **Figure 10C**).
To test this, we propose alternative substitutions in the sequence of U12 snRNA:

1) U_6 for A or G to abolish U6atac A_{26}-U_6: this mutant can only be functional if the binding register includes A_{26}--C_5 and G_{27}=C_4
2) C_5 for U, to introduce A_{26}-U_5, which should be functionally equivalent to A_{26}--C_5
3) C_4 for A or G, which maybe partly functional if the proposed new binding register is wrong and C_4 is indeed a bulge

Comment S2 (to Figure 14)
The stalled transition between 2 steps of splicing in the absence of the Prp16 helicase

The conclusion that the transition between the two steps of splicing is triggered and transacted by the RNA component is consistent with the conservation of the lariat intermediate between the spliceosome and Group II introns, but it seems to disagree with the fact that spliceosome is stalled after the first reaction in the absence of Prp16. But let us consider the dual role of helicases: their role in remodelling of the spliceosome and alternative splicing. Prp16 uses its helicase activity to dissociate suboptimal branch point sites from U2 snRNA, as it is conclusively proven in the experiment with deoxy A substitutions at the branch point (Semlow et al. 2016), but there is no evidence that it translocates along the RNA strand and unwinds RNA duplexes in order to mediate the transition between the 1st and 2nd steps. In fact, there is a proof that it does not translocate across the branch point interaction (Semlow et al. 2016). What is known is that the function of Prp16 is blocked if the nucleotides -4 to -11 at the end of the intron are substituted by their deoxy counterparts. This is the region analogous to the mammalian PPT (**Figure 10A**), the least conserved of the 3' intron end sequences and likely to be involved in RNA-protein interactions. The example of Prp28 highlights, that the helicase activity as such might be not employed in the spliceosome remodelling. Instead, Prp28 initiates the dissociation of the 5' splice site from the U1snRNA by displacing another protein, U1C from the RNA duplex to destabilise it (Chen et al. 2001). Could it be that Prp16 also has a protein target and dissociation of this target facilitates the conformational change of the BP adenosine after branching? This would be a logical explanation, as the preserved ancient mechanism of ribozyme activity would need to be adjusted to maintain the complexity of the spliceosomal proteome, which becomes largely influential for the regulation of alternative splicing in the metazoan genome.

Section S4: Results and Methods - Corelated base pair variation analyses

The purpose of selecting transcripts, in contrast to massive approaches, excludes inferred splicing events and provides real data.

List S1. List of human genes included in the analysis by sKL divergence and Bootstrap
Human genes ranging from well known in medical genetics practice to genes with experimentally confirmed function and expression were chosen at random avoiding paralogues (for example: dystrophin is included, but utrophin is not). Aiming for approximately 2000 introns (splice junctions), 132 genes were included altogether.
(SJ=number of splice junctions, Ch=chromosome)

#	*Gene name*	*SJ*	*Ch*
1	*DMD*, dystrophin	78	X
2	*F5,* coagulation factor V	24	1
3	*CFTR*, cystic fibrosis transmembrane conductance regulator	26	7
4	*F8*, coagulation factor VIII	25	X
5	*F9*, coagulation factor IX	7	X
6	*VWF*, von Willebrand factor	51	12
7	*SMN1*, survival of motor neuron 1	8	5
8	*PAH*, phenylalanine hydroxylase (PKU, phenylketonuria)	12	12
9	*APOE*, apolipoprotein E	3	19
10	*BRCA1*, breast cancer 1, DNA repair associated	23	17
11	*HPRT*, hypoxanthine phosphoribosyltransferase 1	8	X
12	*HBB*, haemoglobin subunit beta	2	11
13	*Fas/CD95*, Fas cell surface death receptor	8	10
14	*TP53*, tumor protein p53	11	17
15	*TNF*, tumour necrosis factor	3	6
16	*UBC*, ubiquitin C	1	12
17	*EGFR*, epidermal growth factor receptor	27	7
18	*VEGFA*, vascular endothelial growth factor	6	6
19	*IL6*, interleukin 6	5	7
20	*TGFB1*, transforming growth factor, beta	6	19
21	*ESR1*, oestrogen receptor 1	9	6
22	HLA-DRB1, major histocompatibility complex, class II, DR beta 1	5	6
23	*NFKB1*, nuclear factor of kappa	23	4
24	*IL10*, interleukin 10	4	1
25	*AKT1*, AKT serine/threonine kinase 1	12	14
26	*CD4*, cluster of differentiation 4 glycoprotein	9	12
27	*GRB2*, growth factor receptor-bound protein 2	5	17
28	*ELP1/IKBKAP,* elongator complex protein 1	36	9
29	*NOVA1*, alternative splicing regulator 1 Neuro-Oncological Ventral Antigen 1	4	14
30	*TOP1*, topoisomerase I	20	20
31	*INS*, insulin	1	11
32	*FHL5/ACT*, four and a half LIM domains 5	6	6
33	*TPM1*, tropomyosin	8	15
34	*DAG1*, dystroglycan 1	5	3
35	*POLR2A*, RNA polymerase II subunit A isoform 201	9	17
36	*PRPF8*, Prp8, pre-mRNA processing factor 8	42	17
37	*DDX8*, Prp22, pre-mRNA processing factor 22	22	17
38	*SNRNP200*, sn ribonucleoprotein U5 subunit 200, Brr2 helicase,	44	2
39	*MYC*, proto-oncogene, bHLH transcription factor	2	8
40	*TBP*, TATA-box binding protein	7	6
41	*LAMB1*, laminin subunit beta 1	33	7
42	*NOTCH1*, notch receptor 1	33	9
43	*SGCA*, sarcoglycan alpha	9	17
44	*TUBA1A,* tubulin	3	12
45	*MYO7A*, myosin VIIA	48	11
46	*NUP155*, nucleoporin 155	34	5
47	*PTBP1*, polypyrimidine tract binding protein 1	14	19
48	*U2AF2*, U2AF65, U2 small nuclear RNA auxiliary factor 2	11	19
49	*GAPDH*, glyceraldehyde-3-phosphate dehydrogenase	8	12
50	*TK1*, thymidine kinase 1	6	17
51	*ATM*, serine/threonine kinase (ataxia telangiectasia mutated)	62	11

52	*TERT*, telomerase reverse transcriptase	15	5
53	*ADAMTS13*, metallopeptidase with thrombospondin type 1 motif 13	28	9
54	*NEU1*, neuraminidase 1	5	6
55	*GLB1*, beta-galactosidase	15	3
56	*CTSA*, cathepsin A	14	20
57	*ACE*, angiotensin I converting enzyme	24	17
58	*ERN1*, endoplasmic reticulum to nucleus signaling 1	21	17
59	*BMP2*, bone morphogenetic protein 2	2	20
60	*CDK6*, cyclin dependent kinase 6	7	7
61	*CCND1*, cyclin D1	4	11
62	*EZH1,* enhancer of zeste 1 polycomb repressive complex 2 subunit	20	17
63	*HAT1*, histone acetyltransferase 1	10	2
64	*ITGB3*, integrin subunit beta 3	14	17
65	*IFNG*, interferon gamma	3	12
66	*ERBB2*, erb-b2 receptor tyrosine kinase 2	30	17
67	*APP*, amyloid beta precursor protein	17	21
68	*EGF*, epidermal growth factor	23	4
69	*CTNNB1*, catenin beta 1	14	3
70	*IGF1*, insulin like growth factor 1	5	12
71	*MAPK1*, mitogen-activated protein kinase 1	8	22
72	*IGHG1*, immunoglobulin heavy constant gamma 1	5	14
73	*TFRC*, transferrin receptor	18	3
74	*CRP*, C-reactive protein	1	1
75	*MET*, proto-oncogene, receptor tyrosine kinase	20	7
76	*RGS6*, regulator of G protein signaling 6	17	14
77	*BCL2*, apoptosis regulator	1	18
78	*XRCC6,* X-ray repair cross complementing 6, Ku70	12	22
79	*RAD52,* RAD52 homolog, DNA repair protein	11	12
80	*MSTN*, myostatin (GDF8, growth and differentiation factor 8)	2	2
81	*PNMT*, phenylethanolamine N-methyltransferase	2	17
82	*CELF1*, CUGBP Elav-like family member 1 (splicing regulator)	15	11
83	*BLM,* RecQ like helicase (Bloom syndrome)	21	15
84	*PSEN1*, FAD, presenilin 1 (γ-secretase subunit, processing of APP)	11	14
85	*AMY1A*, amylase alpha 1A (salivary)	10	1
86	*SPSB1*, splA/ryanodine receptor domain, SOCS box containing-1 (SSB1, ssDNA binding-1)	2	1
87	*G6PD*, glucose-6-phosphate dehydrogenase	12	X
88	*USB1*, U6 snRNA biogenesis phosphodiesterase 1	6	16
89	*ERCC6*, excision repair 6, chromatin remodeling factor	20	10
90	*ATP2B1*, ATPase plasma membrane Ca2+ transporting 1	19	12
91	*ETV4*, ETS variant transcription factor 4	12	17
92	*HNF1A*, HNF1 homeobox A	9	12
93	*NAT2*, N-acetyltransferase 2, AAC2	1	8
94	*LPL*, lipoprotein lipase	9	8
95	*FADS1*, fatty acid desaturase 1	11	11
96	*GDF5*, growth differentiation factor 5	1	20
97	*ACADM*, acyl-CoA dehydrogenase medium chain	11	1
98	*GATA1,* GATA binding protein 1	5	X
99	*GSTM1*, glutathione S-transferase mu 1	7	1
100	*CDH1*, cadherin 1	15	16
101	*FMR1*, fragile X mental	16	X
102	*POLD1*, DNA polymerase delta 1, catalytic subunit	26	19
103	*RPA*, replication protein A	16	17
104	*WRN*, WRN RecQ like helicase	34	8
105	*SP1*, Sp1 transcription factor	5	12
106	*TWIST1*, twist family bHLH transcription factor 1	1	7
107	*TAF1*, TATA-box binding protein associated factor 1	37	X
108	*POLB,* DNA polymerase beta	13	8
109	*TERF2*, telomeric repeat binding factor 2	9	16
110	*RFC1*, replication factor C, subunit 1	24	4
111	*MAPT*, microtubule associated protein tau	14	17
112	*ACTA2, actin alpha 2, smooth muscle*	8	10
113	*PCGF2*, polycomb group ring finger 2	10	17
114	*SSBP3*, single stranded DNA binding protein 3	17	1

115	*CHEK1,* checkpoint kinase 1	12	11
116	*BRIP1,* BRCA1 interacting protein C-terminal helicase 1	19	17
117	*NFATC2*, nuclear factor of activated T cells 2	10	20
118	*PRKDC*, protein kinase, DNA-activated, catalytic subunit, DNA-PKC	85	8
119	*OPTN, optineurin*	14	10
120	*ZBP1,* Z-DNA binding protein 1	7	20
121	*LDHA,* lactate dehydrogenase A	7	11
122	*RBPJ,* recombination signal binding protein for immunoglobulin κ J region	11	4
123	*FKTN,* fukutin	9	9
124	*SFPQ*, splicing factor proline and glutamine rich	9	1
125	*MAD1L1,* mitotic arrest deficient 1 like 1	16	7
126	*RAD51,* recombinase, RecA	9	15
127	*POLA1,* DNA polymerase alpha 1, catalytic subunit	36	X
128	*PCNA,* proliferating cell nuclear antigen	5	20
129	*PRIM2,* DNA primase subunit 2	4	6
130	*ITPR1,* inositol 1,4,5-trisphosphate receptor type 1	58	3
131	*EFNA5,* ephrin A5	4	5
132	*OPA1,* mitochondrial dynamin like GTPase	29	3
	Total SJ: **2007**		

Further description of these genes is provided below, **Figures S12 and S13**.

Description of 132 human genes included in the analysis

The selected genes are responsible for a wide-range of functions (**Figure S12**) and their exon/intron structure is representative of human protein-coding genes (**Figure S13**).

Code: `human_genes_function_pie.py`

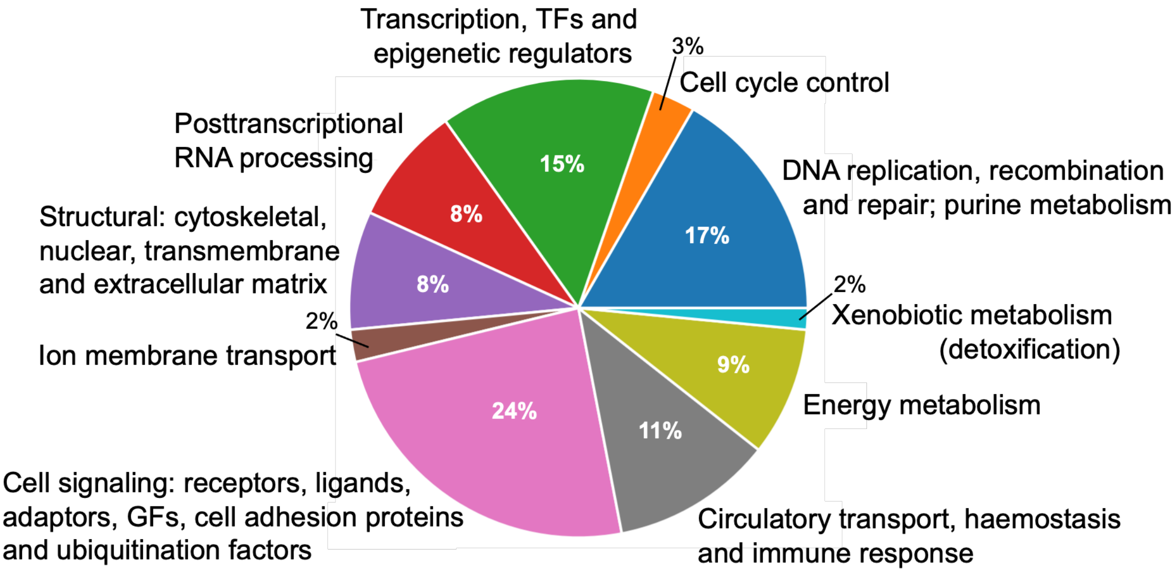

Function	Human genes	Total
**DNA replication, recombination and repair; purine metabolism**	*BRCA1, TOP1, TK1, ATM, TERT, XRCC6, RAD52, BLM, ERCC6, POLD1, RPA1, WRN, POLB, TERF2, RFC1, BRIP1, PRKDC, RAD51, POLA1, PCNA, PRIM2, HPRT*	22
**Cell cycle control**	*CDK6, CCND1, CHEK1, MAD1L1*	4
**Transcription, TFs and epigenetic regulators**	*TP53, ESR1, NFKB1, ELP1/IKBKAP, FHL5, POLR2A, MYC, TBP, EZH1, HAT1, ETV4, HNF1A, GATA1, SP1, TWIST1, TAF1, PCGF2, SSBP3, NFATC2, RBPJ,*	20
**Posttranscriptional RNA processing**	*SMN1, NOVA1, PRPF8, DDX8, SNRNP200, PTBP1, U2AF2, USB1, FMR1, SFPQ, CELF1*	11
**Structural: cytoskeletal, nuclear, transmembrane and extracellular matrix**	*DMD, TPM1, DAG1, LAMB1, SGCA, TUBA1A, MYO7A, NUP155, MAPT, ACTA2, FKTN*	11
**Ion membrane transport**	*CFTR, TFRC, ATP2B1*	3
**Cell signalling: receptors, ligands, adaptors, GFs, cell adhesion proteins and ubiquitination factors**	*Fas/CD95, TNF, UBC, EGFR, VEGFA, TGFB1, AKT1, GRB2, INS, NOTCH1, ERN1, BMP2, ITGB3, ERBB2, APP, EGF, CTNNB1, IGF1, MAPK1, MET, RGS6, BCL2, MSTN, PNMT, PSEN, SPSB1, GDF5, CDH1, OPTN, ZBP1, ITPR1, EFNA5*	32
**Circulatory transport, haemostasis and immune response**	*F5, F8, F9, VWF, APOE, HBB, IL6, HLA-DRB1, IL10, CD4, ADAMTS13, ACE, IFNG, IGHG1, CRP*	15
**Energy metabolism**	*PAH, GADPH, NEU1, GLB1, CTSA, AMY1A, G6PD, LPL, FADS1, ACADM, LDHA, OPA1*	12
**Xenobiotic metabolism (detoxification)**	*NAT2, GSTM1*	2

Figure S12| Functional classification of human genes included in the analysis
This summary is reduced to only 1 function per gene relevant to the transcript isoform included in the analysis. Functional classification of human protein-coding genes is challenging, as more than 1 function can be usually assigned to each individual protein isoform. This problem is much better addressed by co-regulation mapping, that allows flexible and meaningful functional grouping (Kustatscher et al., 2019). The purpose of this rough guide is to show that the datasets of introns and exon junctions were sourced from protein-coding genes with a wide-range of functions.

Code: `human_genes_statistics_Reviewer2.R`

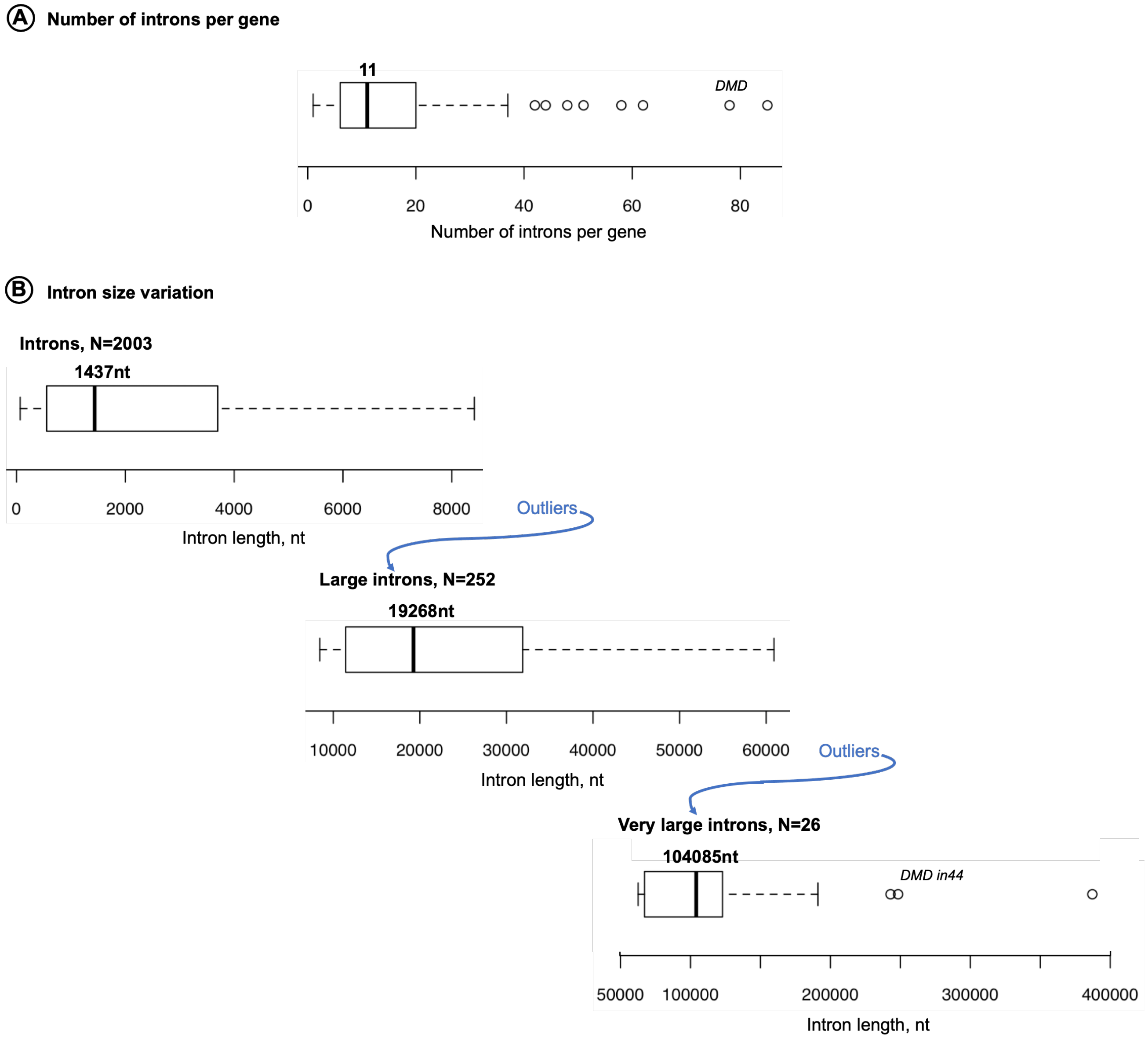

Figure S13| Characterisation of introns in human genes included in the analysis
A: Number of introns per gene: median=11, confidence interval for median: 9.1 to 12.9, range: 1 to 85, mean=15.2, SD=14.6. Statistics for 19116 human protein-coding genes (Piovescan et al., 2019) indicates: median=8, range: 0 to 362 (inclusive of 1068 intron-less genes, 5.6%), mean=10.3, SD=8.6. The average numbers are slightly higher in our selection as we did not include intron-less genes. **B:** Intron size variation: median=1437nt, confidence interval for median: 1326nt to 1548nt, range: 67 to 387219nt, mean=5519nt, SD=17607nt. Piovescan et al., 2019 indicate: median =1742nt, range: 26 to 1160411nt, mean=7397nt, SD=24263nt. The average length estimates are lower in our selection, as the overall giant intron outliers can reach 10^6 nt. The median is less affected by outliers, than the mean and its' standard deviation (SD), therefore the previously reported median is close to our confidence interval, while the differences are larger between means and SDs. Overall, the number of introns per gene and the lengths of introns included in our analysis agree with the statistics for human protein coding-genes. The purpose of selecting transcripts, in contrast to massive approaches, excludes inferred splicing events and provides real data.

>>> splice_sites.py

Human gene sequences were downloaded from *ensembl* with two separate files for exons and introns. The splice isoforms were selected to have:

- Experimental evidence (RefSeq, **TSL1**=transcript support level 1)
- Maximum splice junctions for a particular gene

The *ensembl* isoform number is featured in the sequence file names.

Extracting slice junctions from 132 human genes

```
001_DMD_203_exons.txt
Splice junctions in this gene:  78
002_F5_202_exons.txt
Splice junctions in this gene:  24
003_CFTR_201_exons.txt
Splice junctions in this gene:  26
004_F8_202_exons.txt
Splice junctions in this gene:  25
005_F9_201_exons.txt
Splice junctions in this gene:  7
006_VWF_201_exons.txt
Splice junctions in this gene:  51
007_SMN1_202_exons.txt
Splice junctions in this gene:  8
008_PAH_215_exons.txt
Splice junctions in this gene:  12
009_APOE_201_exons.txt
Splice junctions in this gene:  3
010_BRCA1_210_exons.txt
Splice junctions in this gene:  23
011_HPRT1_201_exons.txt
Splice junctions in this gene:  8
012_HBB_206_exons.txt
Splice junctions in this gene:  2
013_FAS_221_exons.txt
Splice junctions in this gene:  8
014_TP53_223_exons.txt
Splice junctions in this gene:  11
015_TNF_208_exons.txt
Splice junctions in this gene:  3
016_UBC_201_exons.txt
Splice junctions in this gene:  1
017_EGFR_201_exons.txt
Splice junctions in this gene:  27
018_VEGFA_205_exons.txt
Splice junctions in this gene:  6
019_IL6_204_exons.txt
Splice junctions in this gene:  5
020_TGFB1_201_exons.txt
Splice junctions in this gene:  6
021_ESR1_207_exons.txt
Splice junctions in this gene:  9
022_HLA_DRB1_201_exons.txt
Splice junctions in this gene:  5
023_NFKB1_201_exons.txt
Splice junctions in this gene:  23
024_IL10_202_exons.txt
Splice junctions in this gene:  4
025_AKT1_208_exons.txt
Splice junctions in this gene:  12
026_CD4_201_exons.txt
Splice junctions in this gene:  9
027_GRB2_203_exons.txt
Splice junctions in this gene:  5
028_ELP1_201_exons.txt
Splice junctions in this gene:  36
029_NOVA1_206_exons.txt
Splice junctions in this gene:  4
030_TOP1_201_exons.txt
Splice junctions in this gene:  20
031_INS_203_exons.txt
Splice junctions in this gene:  1
032_FHL5_201_exons.txt
Splice junctions in this gene:  6
033_TPM1_206_exons.txt
Splice junctions in this gene:  8
034_DAG1_222_exons.txt
Splice junctions in this gene:  5
035_POLR2A_201_exons.txt
Splice junctions in this gene:  9
036_PRPF8_201_exons.txt
Splice junctions in this gene:  42
037_DHX8_201_exons.txt
Splice junctions in this gene:  22
038_SNRNP200_201_exons.txt
Splice junctions in this gene:  44
039_MYC_207_exons.txt
Splice junctions in this gene:  2
040_TBP_202_exons.txt
Splice junctions in this gene:  7
041_LAMB1_201_exons.txt
Splice junctions in this gene:  33
042_NOTCH1_205_exons.txt
Splice junctions in this gene:  33
043_SGCA_201_exons.txt
Splice junctions in this gene:  9
044_TUBA1A_202_exons.txt
Splice junctions in this gene:  3
045_MYO7A_202_exons.txt
Splice junctions in this gene:  48
046_NUP155_201_exons.txt
Splice junctions in this gene:  34
047_PTBP1_203_exons.txt
Splice junctions in this gene:  14
048_U2AF2_202_exons.txt
Splice junctions in this gene:  11
049_GAPDH_201_exons.txt
Splice junctions in this gene:  8
050_TK1_201_exons.txt
Splice junctions in this gene:  6
051_ATM_201_exons.txt
Splice junctions in this gene:  62
052_TERT_201_exons.txt
Splice junctions in this gene:  15
053_ADAMTS13_206_exons.txt
Splice junctions in this gene:  28
054_NEU1_206_exons.txt
Splice junctions in this gene:  5
055_GLB1_201_exons.txt
Splice junctions in this gene:  15
056_CTSA_204_exons.txt
Splice junctions in this gene:  14
057_ACE_202_exons.txt
Splice junctions in this gene:  24
058_ERN1_201_exons.txt
Splice junctions in this gene:  21
059_BMP2_201_exons.txt
Splice junctions in this gene:  2
060_CDK6_201_exons.txt
Splice junctions in this gene:  7
061_CCND1_201_exons.txt
Splice junctions in this gene:  4
062_EZH1_202_exons.txt
Splice junctions in this gene:  20
063_HAT1_201_exons.txt
Splice junctions in this gene:  10
064_ITGB3_201_exons.txt
Splice junctions in this gene:  14
065_IFNG_201_exons.txt
Splice junctions in this gene:  3
066_ERBB2_219_exons.txt
Splice junctions in this gene:  30
067_APP_201_exons.txt
Splice junctions in this gene:  17
068_EGF_201_exons.txt
Splice junctions in this gene:  23
069_CTNNB1_201_exons.txt
Splice junctions in this gene:  14
070_IGF1_203_exons.txt
Splice junctions in this gene:  5
071_MAPK1_201_exons.txt
Splice junctions in this gene:  8
072_IGHG1_202_exons.txt
Splice junctions in this gene:  5
073_TFRC_201_exons.txt
Splice junctions in this gene:  18
074_CRP_201_exons.txt
Splice junctions in this gene:  1
075_MET_201_exons.txt
Splice junctions in this gene:  20
076_RGS6_209_exons.txt
Splice junctions in this gene:  17
077_BCL2_202_exons.txt
Splice junctions in this gene:  1
078_XRCC6_202_exons.txt
Splice junctions in this gene:  12
079_RAD52_202_exons.txt
Splice junctions in this gene:  11
080_MSTN_201_exons.txt
Splice junctions in this gene:  2
081_PNMT_201_exons.txt
Splice junctions in this gene:  2
082_CELF1_214_exons.txt
Splice junctions in this gene:  15
083_BLM_201_exons.txt
Splice junctions in this gene:  21
084_PSEN1_201_exons.txt
Splice junctions in this gene:  11
085_AMY1A_201_exons.txt
Splice junctions in this gene:  10
086_SPSB1_201_exons.txt
Splice junctions in this gene:  2
087_G6PD_202_exons.txt
Splice junctions in this gene:  12
088_USB1_201_exons.txt
Splice junctions in this gene:  6
089_ERCC6_201_exons.txt
Splice junctions in this gene:  20
090_ATP2B1_201_exons.txt
Splice junctions in this gene:  19
091_ETV4_201_exons.txt
Splice junctions in this gene:  12
092_HNF1A_201_exons.txt
Splice junctions in this gene:  9
093_NAT2_201_exons.txt
Splice junctions in this gene:  1
094_LPL_207_exons.txt
Splice junctions in this gene:  9
095_FADS1_201_exons.txt
Splice junctions in this gene:  11
096_GDF5_201_exons.txt
Splice junctions in this gene:  1
097_ACADM_202_exons.txt
Splice junctions in this gene:  11
098_GATA1_202_exons.txt
Splice junctions in this gene:  5
099_GSTM1_201_exons.txt
Splice junctions in this gene:  7
```

100_CDH1_201_exons.txt
Splice junctions in this gene: 15
101_FMR1_205_exons.txt
Splice junctions in this gene: 16
102_POLD1_205_exons.txt
Splice junctions in this gene: 26
103_RPA1_201_exons.txt
Splice junctions in this gene: 16
104_WRN_201_exons.txt
Splice junctions in this gene: 34
105_SP1_201_exons.txt
Splice junctions in this gene: 5
106_TWIST1_201_exons.txt
Splice junctions in this gene: 1
107_TAF1_203_exons.txt
Splice junctions in this gene: 37
108_POLB_201_exons.txt
Splice junctions in this gene: 13
109_TERF2_201_exons.txt
Splice junctions in this gene: 9
110_RFC1_201_exons.txt
Splice junctions in this gene: 24
111_MAPT_204_exons.txt
Splice junctions in this gene: 14
112_ACTA2_201_exons.txt
Splice junctions in this gene: 8
113_PCGF2_207_exons.txt
Splice junctions in this gene: 10
114_SSBP3_204_exons.txt
Splice junctions in this gene: 17
115_CHEK1_213_exons.txt
Splice junctions in this gene: 12
116_BRIP1_201_exons.txt
Splice junctions in this gene: 19
117_NFATC2_201_exons.txt
Splice junctions in this gene: 10
118_PRKDC_201_exons.txt
Splice junctions in this gene: 85
119_OPTN_202_exons.txt
Splice junctions in this gene: 14
120_ZBP1_201_exons.txt
Splice junctions in this gene: 7
121_LDHA_205_exons.txt
Splice junctions in this gene: 7
122_RBPJ_205_exons.txt
Splice junctions in this gene: 11
123_FKTN_201_exons.txt
Splice junctions in this gene: 9
124_SFPQ_201_exons.txt
Splice junctions in this gene: 9
125_MAD1L1_201_exons.txt
Splice junctions in this gene: 16
126_RAD51_201_exons.txt
Splice junctions in this gene: 9
127_POLA1_201_exons.txt
Splice junctions in this gene: 36
128_PCNA_201_exons.txt
Splice junctions in this gene: 5
129_PRIM2_201_exons.txt
Splice junctions in this gene: 4
130_ITPR1_203_exons.txt
Splice junctions in this gene: 58
131_EFNA5_201_exons.txt
Splice junctions in this gene: 4
132_OPA1_205_exons.txt
Splice junctions in this gene: 29

Splice junctions total: 2007

Extracting introns (starts and ends) from 132 human genes

001_DMD_203_introns.txt
Introns in this gene: 78
002_F5_202_introns.txt
Introns in this gene: 24
003_CFTR_201_introns.txt
Introns in this gene: 26
004_F8_202_introns.txt
Introns in this gene: 25
005_F9_201_introns.txt
Introns in this gene: 7
006_VWF_201_introns.txt
Introns in this gene: 51
007_SMN1_202_introns.txt
Introns in this gene: 8
008_PAH_215_introns.txt
Introns in this gene: 12
009_APOE_201_introns.txt
Introns in this gene: 3
010_BRCA1_210_introns.txt
Introns in this gene: 23
011_HPRT1_201_introns.txt
Introns in this gene: 8
012_HBB_206_introns.txt
Introns in this gene: 2
013_FAS_221_introns.txt
Introns in this gene: 8
014_TP53_223_introns.txt
Introns in this gene: 11
015_TNF_208_introns.txt
Introns in this gene: 3
016_UBC_201_introns.txt
Introns in this gene: 1
017_EGFR_201_introns.txt
Introns in this gene: 27
018_VEGFA_205_introns.txt
Introns in this gene: 6
019_IL6_204_introns.txt
Introns in this gene: 5
020_TGFB1_201_introns.txt
Introns in this gene: 6
021_ESR1_207_introns.txt
Introns in this gene: 9
022_HLA_DRB1_201_introns.txt
Introns in this gene: 5
023_NFKB1_201_introns.txt
Introns in this gene: 23
024_IL10_202_introns.txt
Introns in this gene: 4
025_AKT1_208_introns.txt
Introns in this gene: 12
026_CD4_introns.txt
Introns in this gene: 9
027_GRB2_203_introns.txt
Introns in this gene: 5
028_ELP1_201_introns.txt
Introns in this gene: 36
029_NOVA1_206_introns.txt
Introns in this gene: 4
030_TOP1_201_introns.txt
Introns in this gene: 20
031_INS_203_introns.txt
Introns in this gene: 1
032_FHL5_201_introns.txt
Introns in this gene: 6
033_TPM1_206_introns.txt
Introns in this gene: 8
034_DAG1_222_introns.txt
Introns in this gene: 5
035_POLR2A_201_introns.txt
Introns in this gene: 9
036_PRPF8_201_introns.txt
Introns in this gene: 42
037_DHX8_201_introns.txt
Introns in this gene: 22
038_SNRNP200_201_introns.txt
Introns in this gene: 44
039_MYC_207_introns.txt
Introns in this gene: 2
040_TBP_202_introns.txt
Introns in this gene: 7
041_LAMB1_201_introns.txt
Introns in this gene: 33
042_NOTCH1_205_introns.txt
Introns in this gene: 33
043_SGCA_201_introns.txt
Introns in this gene: 9
044_TUBA1A_202_introns.txt
Introns in this gene: 3
045_MYO7A_202_introns.txt
Introns in this gene: 48
046_NUP155_201_introns.txt
Introns in this gene: 34
047_PTBP1_203_introns.txt
Introns in this gene: 14
048_U2AF2_202_introns.txt
Introns in this gene: 11
049_GAPDH_201_introns.txt
Introns in this gene: 8
050_TK1_201_introns.txt
Introns in this gene: 6
051_ATM_201_introns.txt
Introns in this gene: 62
052_TERT_201_introns.txt
Introns in this gene: 15
053_ADAMTS13_206_introns.txt
Introns in this gene: 28
054_NEU1_206_introns.txt
Introns in this gene: 5
055_GLB1_201_introns.txt
Introns in this gene: 15
056_CTSA_204_introns.txt
Introns in this gene: 14
057_ACE_202_introns.txt
Introns in this gene: 24
058_ERN1_201_introns.txt
Introns in this gene: 21
059_BMP2_201_introns.txt
Introns in this gene: 2
060_CDK6_201_introns.txt
Introns in this gene: 7
061_CCND1_201_introns.txt
Introns in this gene: 4
062_EZH1_202_introns.txt
Introns in this gene: 20
063_HAT1_201_introns.txt
Introns in this gene: 10
064_ITGB3_201_intons.txt
Introns in this gene: 14
065_IFNG_201_introns.txt
Introns in this gene: 3
066_ERBB2_219_introns.txt
Introns in this gene: 30
067_APP_201_introns.txt
Introns in this gene: 17
068_EGF_201_introns.txt
Introns in this gene: 23
069_CTNNB1_201_introns.txt
Introns in this gene: 14
070_IGF1_203_introns.txt
Introns in this gene: 5
071_MAPK1_201_introns.txt
Introns in this gene: 8
072_IGHG1_202_introns.txt
Introns in this gene: 5
073_TFRC_201_introns.txt
Introns in this gene: 18
074_CRP_201_introns.txt

Introns in this gene: 1
075_MET_201_introns.txt
Introns in this gene: 20
076_RGS6_209_introns.txt
Introns in this gene: 17
077_BCL2_202_introns.txt
Introns in this gene: 1
078_XRCC6_202_introns.txt
Introns in this gene: 12
079_RAD52_202_introns.txt
Introns in this gene: 11
080_MSTN_201_introns.txt
Introns in this gene: 2
081_PNMT_201_introns.txt
Introns in this gene: 2
082_CELF1_214_introns.txt
Introns in this gene: 15
083_BLM_201_introns.txt
Introns in this gene: 21
084_PSEN1_201_introns.txt
Introns in this gene: 11
085_AMY1A_201_introns.txt
Introns in this gene: 10
086_SPSB1_201_introns.txt
Introns in this gene: 2
087_G6PD_202_introns.txt
Introns in this gene: 12
088_USB1_201_introns.txt
Introns in this gene: 6
089_ERCC6_201_introns.txt
Introns in this gene: 20
090_ATP2B1_201_introns.txt
Introns in this gene: 19
091_ETV4_201_introns.txt
Introns in this gene: 12
092_HNF1A_201_introns.txt
Introns in this gene: 9
093_NAT2_201_introns.txt
Introns in this gene: 1
094_LPL_207_introns.txt
Introns in this gene: 9
095_FADS1_201_introns.txt
Introns in this gene: 11
096_GDF5_201_introns.txt
Introns in this gene: 1
097_ACADM_202_introns.txt
Introns in this gene: 11
098_GATA1_202_introns.txt
Introns in this gene: 5
099_GSTM1_201_introns.txt
Introns in this gene: 7
100_CDH1_201_introns.txt
Introns in this gene: 15
101_FMR1_205_introns.txt
Introns in this gene: 16
102_POLD1_205_introns.txt
Introns in this gene: 26
103_RPA1_201_introns.txt
Introns in this gene: 16
104_WRN_201_introns.txt
Introns in this gene: 34
105_SP1_201_introns.txt
Introns in this gene: 5
106_TWIST1_201_introns.txt
Introns in this gene: 1
107_TAF1_203_introns.txt
Introns in this gene: 37
108_POLB_201_introns.txt
Introns in this gene: 13
109_TERF2_201_introns.txt
Introns in this gene: 9
110_RFC1_201_introns.txt
Introns in this gene: 24
111_MAPT_204_introns.txt
Introns in this gene: 14
112_ACTA2_201_introns.txt
Introns in this gene: 8
113_PCGF2_207_introns.txt
Introns in this gene: 10
114_SSBP3_204_introns.txt
Introns in this gene: 17
115_CHEK1_213_introns.txt
Introns in this gene: 12
116_BRIP1_201_introns.txt
Introns in this gene: 19
117_NFATC2_201_introns.txt
Introns in this gene: 10
118_PRKDC_201_introns.txt
Introns in this gene: 85
119_OPTN_202_introns.txt
Introns in this gene: 14
120_ZBP1_201_introns.txt
Introns in this gene: 7
121_LDHA_205_introns.txt
Introns in this gene: 7
122_RBPJ_205_introns.txt
Introns in this gene: 11
123_FKTN_201_introns.txt
Introns in this gene: 9
124_SFPQ_201_introns.txt
Introns in this gene: 9
125_MAD1L1_201_introns.txt
Introns in this gene: 16
126_RAD51_201_introns.txt
Introns in this gene: 9
127_POLA1_201_introns.txt
Introns in this gene: 36
128_PCNA_201_introns.txt
Introns in this gene: 5
129_PRIM2_201_introns.txt
Introns in this gene: 4
130_ITPR1_203_introns.txt
Introns in this gene: 58
131_EFNA5_201_introns.txt
Introns in this gene: 4
132_OPA1_205_introns.txt
Introns in this gene: 2

Introns total: 2007

Unusual introns

Order for all sequences printed out below: splice junction (8+3nt), intron start (10nt), intron end (60nt)

Isolating all minor (U12*) introns

Looking for U6atac* binding site motif **AT(+5C)C**

723 CT**T**C**A**TT**G**/CT**G** ATATCCGTGC U6atac/ U12
TCCGGACCCCAGGCCAGTGCTCCCTCTATTCGGCACAAGCCCTT**CCTT**G**AC**AGTCCCCA**G** unusual intron ends combination: AT-AG
735 GT**T**TGG**A**C/ATA GT**ATCCT**ATT U6atac/ U12(?) or U2 **CT**T**AC**
GAAACAAGTTAAAGAAGAATAAGATTCACCTCTGTCTG**C**C**T**T**AC**ATCCACACTACCTCAG
1141 **A**AAG**A**TGT/ATA GT**ATCCTTT**T U6atac/ U12
TTCAATGCTTTGCTAAATGTGACCAGCTAATTGGTGTTTTA**CCTTAAC**CTGTGCAAACAG
1940 TACTCC**AG**/TTT GT**ATCC**AC**T**A U6atac/ U12(?) or U2 **TAAC**
CAGAGTCAAGATGGTATGTTAATG**TAAC**CAATC**T**GTT**T**CC**A**TTTCCA**C**T**T**G**AA**TCTTTAG
[722, 734, 1140, 1939]

Total U12 introns: 4 0.20 %

Looking for U12 binding site

520 TA**T**GTC**AG**/**G**T**G** GTGG**G**CTGGA U6/ U2, major class intron
AAACTCTGATTTTAATTGGGCTT**CCTTAAC**AAGTCTTAATCTCTCCATGTTTTCCTTCAG
833 TT**T**TC**A**G**G**/**G**GA GTAT**GTA**CAT U6/ U2, major class intron
CTGGTCTATGAACAAAACTTTTTAAAACGATGACTGTATTTTTTC**CCTTAAC**TCTGTTAG
1141 **A**AAG**A**TGT/ATA GT**ATCCTTT**T U6atac/ U12
TTCAATGCTTTGCTAAATGTGACCAGCTAATTGGTGTTTTA**CCTTAAC**CTGTGCAAACAG

Caution: U12 motif CCTTAAC and U2 motif TAAC are not easy to separate, U6atac motif (see above) is a safer option

---

**U6atac and U4atac are the names originally given to these snRNA homologues of U6 and U4, as the first minor introns identified had AT_AC ends. It later appeared that most of minor introns still have GT_AC ends and occasionally major introns can also have AT_AC ends. U2 and U1 minor homologues were always named U12 and U11.*

Isolating AU-AC introns

Testing for the first intron G

723 CT**T**C**A**TT**G**/CT**G** ATATCCGTGC U6atac/ U12
TCCGGACCCCAGGCCAGTGCTCCCTCTATTCGGCACAAGCCCTT**CCTT**G**AC**AGTCCCCA**G** unusual intron ends combination: AT-AG

Testing for the last intron G

All the examined introns end with a G

List S2. Minor introns

Minor (U12) introns

#	File	Gene, (ensembl isoform)	Intron	In. bp	Ch
723	045_MYO7A_202_introns	*MYO7A*, myosin VIIA (202)	38 of 48	858	11
735	046_NUP155_201_introns	*NUP155*, nucleoporin 155 (201)	2 of 34	262	5
1141	071_MAPK1_201_introns	*MAPK1*, mitogen-activated protein kinase 1 (201)	2 of 8	604	22
1940	130_ITPR1_203_introns	*ITPR1,* inositol 1,4,5-trisphosphate receptor type 1 (203)	24 of 58	3751	3

GA-AG intron

1942	130_ITPR1_203_introns	*ITPR1,* inositol 1,4,5-trisphosphate receptor type 1(203)	26 of 58	1967	3

Excluding the minor spliceosome (U12) introns from the analysis
Total splice junctions of major introns only: 2003

Isolating GC-AG introns

List S3. Major introns with substitutions of +2U: GC(A)_AG introns

Order for all sequences printed out below: splice junction (8+3nt), intron start (10nt), intron end (60nt)

30 AAGCCCAGAAA GCAAGTACAT
CTTGGAAAGTTAGTTGTTCTTTGTAGAGCATGCTGACTAATAATGCTATCCTCCCAACAG
84 TTTGCAAGCTG GCAAGAAACT
TTTCAATCAACCATAATTTCTTCTCTTGAGTTATTTCATTGTCTTTCTGTCCTAACTCAG
489 GCTATAAGGTG GCACGTAGCA
TGTCCTTTTATTCTTAGATACCTCCTTCACTGAGACCTTTTCCTTACCTCACCTCTCTAG
645 GTGGACAGGTC GCGCGTATAC
GGGGACAGGGAGCTCAGGGAGTGGCAGCTGCCCGGGGCCGACAGCTCCTGTTCCCTGCAG
674 CCTCGTGGTTC GCAAGTTGGG
TTATCCCCTGCCCAGGACTGAGGCTGGCCTGTGTGTTTGGGACTTGTGGGGTCCCCACAG
856 TGATGAAGAGA GCAAGTGTTA
AGATGTGAGAATATTTGAAATACCTTGTTTCTTAATTTTGTGTCTTTTTTTTAATGGTAG
883 CAGGACAGCCC GCAAGTGTGG
AGGGTTGGAGGATGCCACCTCTGGCCTCTTCTGGAACGGAGTCTGATTTTGGCCCCGCAG
1016 AAGCACAGATT GCAAGAAGGG
CCTGCATTTGCAGAGCTCCCTAGGGCCGTGACTGAGACCTGTCCTTCCTGTCTTTTTCAG
1320 AATTACAGGTT GCAAGTGCTC
GCAGTTGATGACTGTATATGCTCTTTCATGGTTGTTTTCTTCTTGGACGTTTGGATGCAG
1643 GGTGCCAGGGA GCAAGTCTGG
CCTCCTGGGTCTCCTGGTTCTTCCTGGGTCTTCCTAGCTTCATTCTTTCTCCGTCCCCAG
1676 CCAGACAGTTA GCAAGTCGGA
GTTCTCTGGTTTTCTTTGTAAGGCGTGTCTCAATATTTGACATTTTCATTTCTGTTGTAG
1942 GGAGGAAGTGA GAAAGTATAT (?)
GGGGCGTGAGAGGAGGCATTTGTCATTCATTTGGCCTTTCCCCACCTTGTGCTCCTTTAG
1961 AACTGAAGAAC GCAAGTAGGA
TTCAGGCCGGGCCCCCAAGCAGCCCCTCTACATTTCCTCTCCCTTTGTTTCTCCAACTAG
[29, 83, 488, 644, 673, 855, 882, 1015, 1319, 1642, 1675, 1941, 1960]

Total GC(A)_AG introns: 13 0.65 %. All GC(A)-AC introns have multiple W-C pairs in U5 and U6 helices at the ex/in boundary.

Isolating the exon/intron boundaries missing both conserved Gs: Exon-end -1Gsub and intron +5G sub

List S4. Introns missing both conserved exon-end -1G and intron +5G
Sequences of exon junctions (8nt/3nt), intron starts (10nt) and intron ends (60nt). Substitutions of the conserved guanines are in **red**. **Bold and underlined** are positions that form Watson-Crick pairs with U5 snRNA (exon junctions) and U6 snRNA (intron starts). In **bold not underlined** are the conserved adenines at intron positions +3 and +4, that form non-canonical pairs with U6 positions 43 and 44. ID numbers refer to lists `splice_junctions_u6, intron_starts_u6, introns_ends_u6` (numbers in brackets refer to lists `splice_junctions, intron_starts, introns_ends` before the data cleaning, which allow to trace the gene and intron number).

899 (908) GCAGTCCA/**G**T**G** GTT**A**T**G**TC**C**T *compensation with +9C=G pair?*
GAGCCTGCCCTGCTGGGAATCGGGGAAGCAC**T**G**CT**T**AC**CTGTCTCCTGCTCCCTTTT**C**AG *[very strong 3' end interactions]*

Compensation with multiple other W-C pairs of the U5 and U6 binding sites
984 (993) **A**AAG**AAAA**/**G**T**G** GT**AAC**T**T**TCGC
AGGAAGGCTTCTGGGCTGTCTGATTGCACTTTCTTCTTATCCTCCCGTCTCCTCCTTTAG
1220 (1231) **A**T**TAA**TCA/AGA GT**AAATA**C**C**T
TGCTTTCTGTGAGAATACTTCATTATTTGCTGATGACGCACTTTTCCTTGACCTCTGTAG
1278 (1289) TACTT**AAA**/**G**AA GT**AAATA**AAT
AGTTACTCTCAAACTATTGTGAAATGATACATCAACGTATATCTTATGTTTCAAAAATAG
1370 (1382) CAG**AAAAA**/**G**GT GT**AATTA**AAT
TTTGTGAACAGTGCTTTTGATTGTTCTACATGGCATATTCACATCCATTTTCTTCCACAG
1714 (1728) **A**AACT**AAA**/**G**TT GT**A**CT**T**C**T**AT
AATATTCTCTTGTCATCAACGTGATAACTTAAACATTGCTGACCTCCTGGTCTGTTTTAG

Total exon/intron boundaries missing both conserved Gs: 6   0.30 %
>>>

Human mutation data explained by the U5 hypothesis

Fu et al., 2011 Mutations of exon-start guanine: G_{+1} → *T* or G_{+1} → *A*

List S5. Mutations of exon-start guanine: G_{+1} → *T* or G_{+1} → *A* (Fu et al., 2011)

Gene	Ex	PSI	U5 base pairs: Watson-Crick / Isosteric / Non-isosteric	U5 model support	U2 base pairs: Watson-Crick (BP, BP+C_{-3}) / Isosteric / Non-isosteric	PPT Stretch (PPS)
GH1	3	1	*T*AA	Yes	cgtagA-26cc… t-3 (1,1)	10
FECH	9	17	*T*TT	Yes	cacttA-25cg…t-3 (4,4)	6
EYA1	10	66	*T*AT	Yes	tcttcA-22cc…t-3 (3,3)	5
LPL	5	100	*T*CC	Yes	aatttA-34ca…a-3 (4,4)	14
HEXA	13	100	*A*CC	Yes	gcccaA-27tc…c-3 (2,3)	13
PKHD1	25	99<br>0*	upstream cryptic agACG<br>instead of *T*TC	Yes	aaaaaA-20ct…t-3 (3,3)<br>atgtaA-10cc…c-3 (3,4)	6<br>3
COL1A2	37	41	*T*GT	Yes	ctgtaA-18ct…c-3 (3,4)	5
CLCN2	19	81	*A*GA	No⟶	tgggaA-6ca…c-3 (4,5)	4
LAMA2	24	100	*A*TG	Yes	tataaA-28ct…c-3 (4,5)	9
CAPN3	10	91	*T*CT	Yes⟶	ctgtgA-31cc…a-3 (2,2)	10
CAPN3	17	0	*T*TT	Yes	cattcA-36ca…a-3 (4,4)	9
NEU1	2	100	*A*TG	Yes	tgttgA-22cc…c-3 (3,4)	14
COL6A2	8	100	*T*GC	No⟶	cactaA-26tg…c-3 (4,5)	15
COL1A1	23	100	*A*GC	No⟶	*cct*ctA-27tc…t-3 (0,0)**	16

* 0% PSI for *PKHD1* exon 25. Longer exon with cryptic 3'ss is included with PSI 99% and exon skipping amounts for 1%. We used alternatively PSI 0 or PSI 99 for the plots, Welch's ANOVA and Kruskal-Wallis test.

>PKHD1-202 intron 24:protein_coding

GTATGTATAGTATCCCTTCTAGTGGCATGGATTTGCTGCCCTGGAGAAATTCTGCAAAAAAAAAAATCAGA
ATTATACAAATGGAAACTGTCATGAATTATTAATTTTGTTGGAATATTTGAGTAAAGGGATTCTATGAACTTT
CTTTCTGGAAGTTTTCAACACATATTTTGTATATCATATCCCTGAAAAAGCAATTTAAACCCAACATCTCCC
TTCCTAACTCAGTGTAGGTAGTGAAGTTATTGGTCAGTGTCCACCATGCAATAATTCTACATCTGTTATTG
GATAATTTAAGCAGATGTGATTGACTTAGATGATTGTGGAAGCAGATGGCATAGCAGAAGGAATTTTAGAA
AAGAATTTGCTGTCTTTGGGTAATGACAAAACCAGATGCTCTTGCATATTTAGGGTCTTAAAAAATAATCTA
ATGATCAACTAGCATTTGGTTATTATAAAATGAAGCCCAAGATGAAAATTTTTCCTAGTTGTATGGTGTTGT
GGGTTATCTGGTTTTCAATCGAAATTCTAGAAAGGAGATGGAGGAAGAATGGGAGACCTTGCTCTTATCT
CATGGCACTGTTTCTTTCCACTTTATTTTAATATTTTGGATTGCAAACATATAAAATGGAAAAGCATGGAGA
AGCAAAGTGTCTCAAGACTTAGAGAGAAGATTGATTTATTGTACCTCTTTTGGCAATCCACTTGCAAATGA
ATATTTCTCTAATCTACTGGGACTTTCCTCAGCCATTATTTACTGTTACGTTTCTATATTTCAATTCTCTGTT
ACTCTGCTTTCACTTTCTAGCTGTCTGATTATCTGAAACACAATGTGAAAAAACTCTTTATTATTTGATTAG|

BP PPS Cryptic 3'ss

ACGAGATTAGATTTCGGTTCCATGACAGAATTTACCAGAAATGTAACCATCTCAG

** *COL1A1* intron 22 is only 113bp long, it has only 1 conserved major intron position (+5G), however, it has 5 conserved minor intron positions: +6C and +7T and branchpoint helix CCT. There is also a super perfect PPT: an uninterrupted stretch of 16 uridines. As it is likely a U12 intron we also tested excluding this data point.

>COL1A2-201 intron 22:protein_coding

GTAAG*CT*GTCTATCACTTACTTCCTAGAAAGGGGCTTGCTGCTTCTGGTGGTGGGTGTGTCATTAGCTTTA
GCATCCTCCT*CCT*CTATCTGTTTTTTTTTTTTTTTTGAATAG

BP PPS

Can we state that the presence of +2C or/and +3G that form Watson-Crick pairs with U5 Loop1 according to our new model is a strong factor that promotes exon inclusion in spite of +1G mutations? We explore the other factors that are expected to influence PSI:

The effect of the exon-start +1G mutations on the exon inclusion efficiency (PSI)

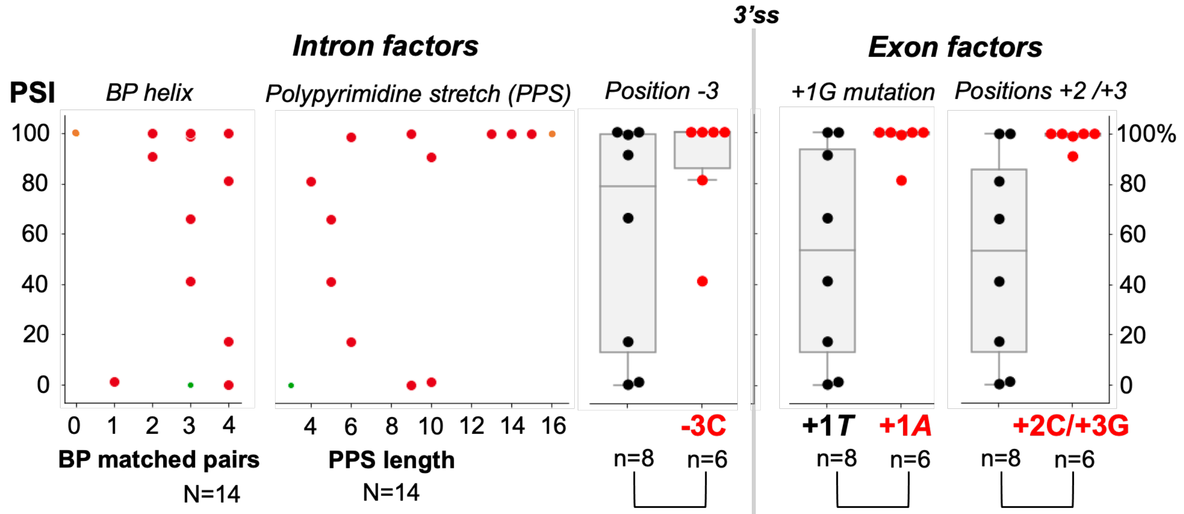

Spearman's correlation	ρ=–0.05 P=8.7e-01	ρ=0.64 **P=1.4e-02**	*Welch's ANOVA*	$F_{0.09}$=2.2, P = 1.7e-01	$F_{0.13}$=8.3, **P = 2.2e-02**	$F_{0.14}$=10.2, **P = 1.5e-02**
			Kruskal-Wallis	H_1=2.18, P = 1.4e-01	H_1=4.06, **P = 4.4e-02**	H_1=4.62, **P = 3.2e-02**

Excluding U12 datapoint •

Spearman's correlation	ρ=0.09 P=7.6e-01	ρ=0.58 **P=3.9e-02**	*Welch's ANOVA*	$F_{0.11}$= 2.8, P = 1.3e-01	$F_{0.13}$=7.9, **P = 2.3e-02**	$F_{0.16}$=13.0, **P = 1.1e-02**
			Kruskal-Wallis	H_1=3.11, P = 7.8e-02	H_1=3.27, P = 7.1e-02	H_1=6.24, **P = 1.2e-02**

Figure S11| Inclusion of exon affected by +1G mutation (data from Fu et al., 2011) is influenced by multiple *cis* factors. In agreement with Fu and colleagues PPS length comes up as a significant intronic factor (PPS, polypyrimidine stretch, uninterrupted by purines, as opposed to PPT, polypyrimidine tract, used generally in this article, for variable sequence, not exclusively pyrimidines, between the branchpoint and intron position -3). We add that the exon-start sequence is also a strong factor of exon inclusion. Nine of these 14 mutations were +1G → *T* with PSI ranging 0-100% and five were +1G → *A* with consistently high PSI 81-100%. Given that exon-start +1G is conserved at 50% in humans, we can expect that a change for purine is better tolerated (generally +1A is twice more likely than +1T). Specifically, we looked for cytosines in exon position +2 and guanines in exon position +3 that form Watson-Crick pairs with U5 Loop1 according to our new model. Indeed, all +1G mutations followed by +2C/+3G have PSI above 90%, which suggests a compensatory mechanism in agreement with our new U5 binding register. **PSI:** percent spliced-in

Excluding U12 datapoint • in scatterplots refers to +1G mutation in *COL1A1* exon 23
Unusual *COL1A1* intron 22 is only 113bp long, it has only 1 conserved major intron position (+5G), however, it has 5 conserved minor intron positions: +6C and +7T and branchpoint motif CCT. There is also a super perfect PPT: an uninterrupted stretch of 16 uridines. As it is likely a U12 intron we also tested excluding this data point.

>COL1A2-201 intron 22:protein_coding
GTAA**G***CT*GTCTATCACTTACTTCCTAGAAAGGGGCTTGCTGCTTCTGGTGGTGGGTGTGTCATTAGCTTTA
GCATCCTCCT*CCT*CT**A**TCTGTTTTTTTTTTTTTTTTGAATAG
BP PPS

• datapoint in scatterplots refers to *PKHD* exon 25 PSI 0, not included in the boxplots. *PKHD* cryptic 3'ss (longer exon 25) with PSI 99 appears both in scatterplots and in the boxplots and was used exclusively for statistical tests presented here (see below pages 23-32 for all alternative calculations).

Table S4. Factors that promote efficient exon inclusion (PSI 81-100%) in spite of exon-start (+1G) mutations (red dots on the boxplots **Figure S11**). Exon inclusion for five +1G mutations (grey background) are supported by at least two factors out of +2C/+3G, +1G→*A* and -3C. *COL1A1* exon 23 is associated with U12 intron 22 and can be excluded from the analysis (see legend to **Figure S**). Therefore, +1G→*A* does not come up as an independent factor. *LPL* exon 5 and *COL6A2* exon 8 are both additionally supported by 4 matched (Watson-Crick) pairs in the branchpoint helix and a long PPS. The high rate of *CAPN3* exon 10 inclusion is left to be explained solely by +2C/+3G. (Key: *GENE* exon: BP matched pairs PPS length)

**Exon +2C/+3G**	**Exon +1G→*A***	**Intron -3C**
**PSI 100**		
HEXA exon 13: 2 **13**	*HEXA* exon 13: 2 **13**	*HEXA* exon 13: 2 **13**
LAMA exon 24: **4** 9	*LAMA* exon 24: **4** 9	*LAMA* exon 24: **4** 9
NEU1 exon 10: 3 **14**	*NEU1* exon 10: 3 **14**	*NEU1* exon 10: 3 **14**
LPL exon 5: **4 14**		
	COL1A1 exon 23: **0* 16**	
		COL6A2 exon 8: **4 15**
**PSI 99**		
PKHD **cryptic** ex. 25: 3 6	*PKHD* **cryptic** ex. 25: 3 6	
**PSI 91**		
CAPN3 exon 10: 2 10		
	**PSI 81**	
	CLNC2 exon 19: **4** 4	*CLNC2* exon 19: **4** 4

Kruskal-Wallis rank sum test and **Welch's** ANOVA (t-test) for +2C/+3G

1. *Include COL1A1 and PKHD normal 3'ss PSI 0 (N=14)*

	PSI
U5 Watson-Crick pairs	*LPL:* 100, *HEXA:* 100, *LAMA2:* 100, *CAPN3*ex10: 91, *NEU1:* 100
No U5 Watson-Crick pairs	*GH1:* 1, *FECH:* 17, *EYA1:* 66, *COL1A2:* 41, *CLCN2:* 81, *COL6A2:* 100, *COL1A1:* 100, *CAPN3*ex17: 0, *PKHD1:* 0

```
U5_WatsonCrick=[100,100,100,91,100]#n=5
U5_noWatsonCrick=[1,17,66,41,81,100,100,0,0]#n=9
from scipy import stats
###Kruskal-Wallis rank sum test and Welch's t-test for +2C/+3G
print(stats.kruskal(U5_WatsonCrick, U5_noWatsonCrick))
print(stats.ttest_ind(U5_WatsonCrick, U5_noWatsonCrick, equal_var = False))
KruskalResult(statistic=5.255847255369932, pvalue=0.021873149625322268)
Ttest_indResult(statistic=3.7021033170416677, pvalue=0.005702540534091117)
```

2. *Include COL1A1 and PKHD cryptic 3'ss PSI 99 (N=14)*

	PSI
U5 Watson-Crick pairs	*LPL:* 100, *HEXA:* 100, *LAMA2:* 100, *CAPN3*ex10: 91, *NEU1:* 100, *PKHD1:* 99
No U5 Watson-Crick pairs	*GH1:* 1, *FECH:* 17, *EYA1:* 66, *COL1A2:* 41, *CLCN2:* 81, *COL6A2:* 100, *COL1A1:* 100, *CAPN3*ex17: 0

```
U5_WatsonCrick=[100,100,100,91,100,99]#n=6
U5_noWatsonCrick=[1,17,66,41,81,100,100,0]#n=8
KruskalResult(statistic=4.622222222222221, pvalue=0.03156032503831624)
Ttest_indResult(statistic=3.196999122936383, pvalue=0.01473492336832155 6)
```

3. *Include PKHD normal 3'ss PSI 0 and exclude COL1A1 (N=13)*

	PSI
U5 Watson-Crick pairs	*LPL:* 100, *HEXA:* 100, *LAMA2:* 100, *CAPN3*ex10: 91, *NEU1:* 100
No U5 Watson-Crick pairs	*GH1:* 1, *FECH:* 17, *EYA1:* 66, *COL1A2:* 41, *CLCN2:* 81, *COL6A2:* 100, *CAPN3*ex17: 0, *PKHD1:* 0

```
U5_WatsonCrick=[100,100,100,91,100]#n=5
U5_noWatsonCrick=[1,17,66,41,81,100,0,0] #n=8
KruskalResult(statistic=6.57201166180758, pvalue=0.01035947924583537)
Ttest_indResult(statistic=4.208231542954459, pvalue=0.0037207957451188445)
```

4. *Include PKHD cryptic 3'ss PSI 99 and exclude COL1A1 (N=13)*

	PSI
U5 Watson-Crick pairs	*LPL:* 100, *HEXA:* 100, *LAMA2:* 100, *CAPN3*ex10: 91, *NEU1:* 100, *PKHD1:* 99
No U5 Watson-Crick pairs	*GH1:* 1, *FECH:* 17, *EYA1:* 66, *COL1A2:* 41, *CLCN2:* 81, *COL6A2:* 100, *CAPN3*ex17: 0

```
U5_WatsonCrick=[100,100,100,91,100,99]#n=6
U5_noWatsonCrick=[1,17,66,41,81,100,0] #n=7
KruskalResult(statistic=6.240863787375417, pvalue=0.012483557594188328)
Ttest_indResult(statistic=3.6121657705778767, pvalue=0.010840625573149486)
```

R: Kruskal-Wallis rank sum test and **Welch's** ANOVA (t-test) for **+2C/+3G**

1. ***Include COL1A1 and PKHD normal 3'ss PSI 0 (N=14)***

```
### Calculations from lists (unequal sizes are acceptable)
U5_WatsonCrick=c(100,100,100,91,100)#n=5
U5_noWatsonCrick=c(1,17,66,41,81,100,100,0,0)#n=9

> kruskal.test(list(U5_WatsonCrick,U5_noWatsonCrick))#uses vectors without NaNs

	Kruskal-Wallis rank sum test

data:  list(U5_WatsonCrick, U5_noWatsonCrick)
Kruskal-Wallis chi-squared = 5.2558, df = 1, p-value = 0.02187

> shapiro.test(U5_WatsonCrick)$p.value #uses vector without NaNs
[1] 0.0001309782
> shapiro.test(U5_noWatsonCrick)$p.value #uses vector without NaNs
[1] 0.09080469
> sd(U5_WatsonCrick) #uses vector without NaNs
[1] 4.024922
> sd(U5_noWatsonCrick) #uses vector without NaNs
[1] 42.68034
> (sd(U5_WatsonCrick))^2 #uses vector without NaNs
[1] 16.2
> (sd(U5_noWatsonCrick))^2 #uses vector without NaNs
[1] 1821.611

> t.test(U5_WatsonCrick,U5_noWatsonCrick,var.equal = FALSE)# vectors without NaNs

	Welch Two Sample t-test

data:  U5_WatsonCrick and U5_noWatsonCrick
t = 3.7021, df = 8.2539, p-value = 0.005703
alternative hypothesis: true difference in means is not equal to 0
95 percent confidence interval:
 20.19659 85.98118
sample estimates:
mean of x mean of y 
 98.20000  45.11111 

###Dataframe (requires Nans for unequal size groups)
U5_WatsonCrick=c(100,100,100,91,100,NaN,NaN,NaN,NaN)#n=5
U5_noWatsonCrick=c(1,17,66,41,81,100,100,0,0)#n=9
U5_table=data.frame(cbind(U5_WatsonCrick,U5_noWatsonCrick))# vectors with NaNs
U5_table_stacked=stack(U5_table)

#kruskal.test and t.test produce the same result as with lists without NaNs
kruskal.test(values~ind,U5_table_stacked)#uses df with NaNs
t.test(values~ind,U5_table_stacked, var.equal = FALSE)#uses df with NaNs

> oneway.test(values~ind,U5_table_stacked, var.equal = FALSE)#uses df with NaNs

	One-way analysis of means (not assuming equal variances)

data:  values and ind
```

```
F = 13.706, num df = 1.0000, denom df = 8.2539, p-value = 0.005703
```

2. ***Include COL1A1 and PKHD cryptic 3'ss PSI 99 (N=14)***

```
### Calculations from lists (unequal sizes are acceptable)
U5_WatsonCrick=c(100,100,100,91,100,99)#n=6
U5_noWatsonCrick=c(1,17,66,41,81,100,100,0)#n=8

> kruskal.test(list(U5_WatsonCrick,U5_noWatsonCrick))#uses vectors without NaNs

	Kruskal-Wallis rank sum test

data:  list(U5_WatsonCrick, U5_noWatsonCrick)
Kruskal-Wallis chi-squared = 4.6222, df = 1, p-value = 0.03156

> shapiro.test(U5_WatsonCrick)$p.value #uses vector without NaNs
[1] 0.0001621368
> shapiro.test(U5_noWatsonCrick)$p.value #uses vector without NaNs
[1] 0.2241062
> sd(U5_WatsonCrick) #uses vector without NaNs
[1] 3.614784
> sd(U5_noWatsonCrick) #uses vector without NaNs
[1] 41.89016
> (sd(U5_WatsonCrick))^2 #uses vector without NaNs
[1] 13.06667
> (sd(U5_noWatsonCrick))^2 #uses vector without NaNs
[1] 1754.786
> t.test(U5_WatsonCrick,U5_noWatsonCrick,var.equal = FALSE)# vectors without NaNs

	Welch Two Sample t-test

data:  U5_WatsonCrick and U5_noWatsonCrick
t = 3.197, df = 7.1387, p-value = 0.01473
alternative hypothesis: true difference in means is not equal to 0
95 percent confidence interval:
 12.52701 82.63966
sample estimates:
mean of x mean of y 
 98.33333  50.75000 

###Dataframe (requires Nans for unequal size groups)
U5_WatsonCrick=c(100,100,100,91,100,99,NaN,NaN)#n=6
U5_noWatsonCrick=c(1,17,66,41,81,100,100,0)#n=8
U5_table=data.frame(cbind(U5_WatsonCrick,U5_noWatsonCrick))# vectors with NaNs
U5_table_stacked=stack(U5_table)

#kruskal.test and t.test produce the same result as with lists without NaNs
kruskal.test(values~ind,U5_table_stacked)#uses df with NaNs
t.test(values~ind,U5_table_stacked, var.equal = FALSE)#uses df with NaNs

> oneway.test(values~ind,U5_table_stacked, var.equal = FALSE)#uses df with NaNs

	One-way analysis of means (not assuming equal variances)

data:  values and ind
F = 10.221, num df = 1.0000, denom df = 7.1387, p-value = 0.01473
```

4.Include COL1A1 and PKHD cryptic 3'ss PSI 99 (N=14)

```
### Calculations from lists (unequal sizes are acceptable)
U5_WatsonCrick=c(100,100,100,91,100,99)#n=6
U5_noWatsonCrick=c(1,17,66,41,81,100,0) #n=7

> kruskal.test(list(U5_WatsonCrick,U5_noWatsonCrick))#uses vectors without NaNs

	Kruskal-Wallis rank sum test

data:  list(U5_WatsonCrick, U5_noWatsonCrick)
Kruskal-Wallis chi-squared = 6.2409, df = 1, p-value = 0.01248

> shapiro.test(U5_WatsonCrick)$p.value #uses vector without NaNs
[1] 0.0001621368
> shapiro.test(U5_noWatsonCrick)$p.value #uses vector without NaNs
[1] 0.4577796
> sd(U5_WatsonCrick) #uses vector without NaNs
[1] 3.614784
> sd(U5_noWatsonCrick) #uses vector without NaNs
[1] 39.81505
> (sd(U5_WatsonCrick))^2 #uses vector without NaNs
[1] 13.06667
> (sd(U5_noWatsonCrick))^2 #uses vector without NaNs
[1] 1585.238
> t.test(U5_WatsonCrick,U5_noWatsonCrick,var.equal = FALSE)#uses vectors without NaNs

	Welch Two Sample t-test

data:  U5_WatsonCrick and U5_noWatsonCrick
t = 3.6122, df = 6.1153, p-value = 0.01084
alternative hypothesis: true difference in means is not equal to 0
95 percent confidence interval:
 17.78805 91.45005
sample estimates:
mean of x mean of y
 98.33333  43.71429

###Dataframe (requires Nans for unequal size groups)
U5_WatsonCrick=c(100,100,100,91,100,99,NaN)#n=6
U5_noWatsonCrick=c(1,17,66,41,81,100,0) #n=7
U5_table=data.frame(cbind(U5_WatsonCrick,U5_noWatsonCrick))# vectors with NaNs
U5_table_stacked=stack(U5_table)

#kruskal.test and t.test produce the same result as with lists without NaNs
kruskal.test(values~ind,U5_table_stacked)#uses df with NaNs
t.test(values~ind,U5_table_stacked, var.equal = FALSE)#uses df with NaNs

> oneway.test(values~ind,U5_table_stacked, var.equal = FALSE)#uses df with NaNs

	One-way analysis of means (not assuming equal variances)

data:  values and ind
F = 13.048, num df = 1.0000, denom df = 6.1153, p-value = 0.01084
```

Welch’s ANOVA and Kruskal-Wallis rank sum test for substitute *A* or *T*

1. *Include COL1A1 and PKHD normal 3’ss PSI 0 (N=14)*

	PSI
A	*HEXA:* 100, *LAMA2:* 100, *CLCN2:* 81, *NEU1:* 100, *COL1A1:* 100
T	*GH1:* 1, *FECH:* 17, *EYA1:* 66, *LPL:* 100, *COL1A2:* 41, *CAPN3*ex10: 91, *COL6A2:* 100, *CAPN3*ex17: 0, *PKHD1:* 0

```
a=[100,100,81,100,100]#n=5
t=[1,17,66,100,41,91,100,0,0]#n=9
KruskalResult(statistic=4.638080084858127, pvalue=0.031269966652780735)
Ttest_indResult(statistic=3.3095330554652915, pvalue=0.009041709556861968)
```

2. *Include COL1A1 and PKHD cryptic 3’ss PSI 99 (N=14)*

	PSI
A	*HEXA:* 100, *LAMA2:* 100, *CLCN2:* 81, *NEU1:* 100, *COL1A1:* 100, *PKHD1:* 99
T	*GH1:* 1, *FECH:* 17, *EYA1:* 66, *LPL:* 100, *COL1A2:* 41, *CAPN3*ex10: 91, *COL6A2:* 100, *CAPN3*ex17: 0

```
a=[100,100,81,100,100,99]#n=6
t=[1,17,66,100,41,91,100,0]#n=8
KruskalResult(statistic=4.0625, pvalue=0.04384554166017646)
Ttest_indResult(statistic=2.873903456290757, pvalue=0.02188473533338844)
```

3. *Include PKHD normal 3’ss PSI 0 and exclude COL1A1 (N=13)*

	PSI
A	*HEXA:* 100, *LAMA2:* 100, *CLCN2:* 81, *NEU1:* 100
T	*GH1:* 1, *FECH:* 17, *EYA1:* 66, *LPL:* 100, *COL1A2:* 41, *CAPN3*ex10: 91, *COL6A2:* 100, *CAPN3*ex17: 0, *PKHD1:* 0

```
a=[100,100,81,100]#n=4
t=[1,17,66,100,41,91,100,0,0]#n=9
KruskalResult(statistic=3.6384839650145793, pvalue=0.056458322364294 41)
Ttest_indResult(statistic=3.1903049635301564, pvalue=0.010284290272625068)
```

4. *Include PKHD cryptic 3’ss PSI 99 and exclude COL1A1 (N=13)*

	PSI
A	*HEXA:* 100, *LAMA2:* 100, *CLCN2:* 81, *NEU1:* 100, *PKHD1:* 99
T	*GH1:* 1, *FECH:* 17, *EYA1:* 66, *LPL:* 100, *COL1A2:* 41, *CAPN3*ex10: 91, *COL6A2:* 100, *CAPN3*ex17: 0

```
a=[100,100,81,100,99]#n=5
t=[1,17,66,100,41,91,100,0]#n=8
KruskalResult(statistic=3.2651162790697636, pvalue=0.07076800545138776)
Ttest_indResult(statistic=2.806397062740887, pvalue=0.02347286415692591 3)
```

2. *Include COL1A1 and PKHD cryptic 3'ss PSI 99 (N=14)*

```
### Calculations from lists (unequal sizes are acceptable)
a=c(100,100,81,100,100,99)#n=6
t=c(1,17,66,100,41,91,100,0)#n=8

> kruskal.test(list(a,t))#uses vectors without NaNs

	Kruskal-Wallis rank sum test

data:  list(a, t)
Kruskal-Wallis chi-squared = 4.0625, df = 1, p-value = 0.04385

> shapiro.test(a)$p.value
[1] 5.761099e-05
> shapiro.test(t)$p.value
[1] 0.1474304
> sd(a)
[1] 7.685484
> sd(t)
[1] 43.05478
> (sd(a))^2
[1] 59.06667
> (sd(t))^2
[1] 1853.714

> t.test(a,t,var.equal = FALSE)# vectors without NaNs

	Welch Two Sample t-test

data:  a and t
t = 2.8739, df = 7.5883, p-value = 0.02188
alternative hypothesis: true difference in means is not equal to 0
95 percent confidence interval:
  8.485037 80.848297
sample estimates:
mean of x mean of y 
 96.66667  52.00000 

###Dataframe (requires NaNs added to smaller groups to make sizes equal)
a=c(100,100,81,100,100,99,NaN,NaN)#n=6
t=c(1,17,66,100,41,91,100,0)#n=8
U5_table=data.frame(cbind(a,t))
U5_table_stacked=stack(U5_table)

#kruskal.test and t.test produce the same result as with lists without NaNs
kruskal.test(values~ind,U5_table_stacked)#uses df with NaNs
t.test(values~ind,U5_table_stacked, var.equal = FALSE)#uses df with NaNs

> oneway.test(values~ind,U5_table_stacked, var.equal = FALSE)#uses df with NaNs

	One-way analysis of means (not assuming equal variances)

data:  values and ind
F = 8.2593, num df = 1.0000, denom df = 7.5883, p-value =0.02188
```

4. *Include PKHD cryptic 3'ss PSI 99 and exclude COL1A1 (N=13)*

```
### Calculations from lists (unequal sizes are acceptable)
a=c(100,100,81,100,99)#n=5
t=c(1,17,66,100,41,91,100,0) #n=8

> kruskal.test(list(a,t))#uses vectors without NaNs

     Kruskal-Wallis rank sum test

data:  list(a, t)
Kruskal-Wallis chi-squared = 3.2651, df = 1, p-value = 0.07077

> shapiro.test(a)$p.value
[1] 0.0004215357
> shapiro.test(t)$p.value
[1] 0.1474304
> sd(a)
[1] 8.396428
> sd(t)
[1] 43.05478
> (sd(a))^2
[1] 70.5
> (sd(t))^2
[1] 1853.714

> t.test(a,t,var.equal = FALSE)#uses vectors without NaNs

     Welch Two Sample t-test

data:  a and t
t = 2.8064, df = 7.8271, p-value = 0.02347
alternative hypothesis: true difference in means is not equal to 0
95 percent confidence interval:
  7.705914 80.294086
sample estimates:
mean of x mean of y 
       96        52 

###Dataframe (requires NaNs added to smaller groups to make sizes equal)
a=c(100,100,81,100,99,NaN,NaN,NaN)#n=5
t=c(1,17,66,100,41,91,100,0) #n=8
U5_table=data.frame(cbind(a,t))
U5_table_stacked=stack(U5_table)

#kruskal.test and t.test produce the same result as with lists without NaNs
kruskal.test(values~ind,U5_table_stacked)#uses df with NaNs
t.test(values~ind,U5_table_stacked, var.equal = FALSE)#uses df with NaNs

> oneway.test(values~ind,U5_table_stacked, var.equal = FALSE)#uses df with NaNs

     One-way analysis of means (not assuming equal variances)

data:  values and ind
F = 7.8759, num df = 1.0000, denom df = 7.8271, p-value = 0.02347
```

Welch's ANOVA and Kruskal-Wallis rank sum test for -3C

1. *Include COL1A1 and PKHD normal 3'ss PSI 0 (N=14)*

	PSI
-3C	*HEXA:* 100, *COL1A2:* 41, *CLCN2:* 81, *LAMA2:* 100, *NEU1:* 100, *COL6A2:* 100, *PKHD1:* 0
-3Csub	*GH1:* 1, *FECH:* 17, *EYA1:* 66, *LPL:* 100, *CAPN3*ex10: 91, *COL1A1:* 100, *CAPN3*ex17: 0,

```
pos3C=[100,41,81,100,100,100,0]#n=7
pos3Csub=[1,17,66,100,91,100,0]#n=7
KruskalResult(statistic=0.8687350835322164, pvalue=0.351305729128759)
Ttest_indResult(statistic=0.9143731510776307, pvalue=0.37897292811768657)
```

2. *Include COL1A1 and PKHD cryptic 3'ss PSI 99 (N=14)*

	PSI
-3C	*HEXA:* 100, *COL1A2:* 41, *CLCN2:* 81, *LAMA2:* 100, *NEU1:* 100, *COL6A2:* 100
-3Csub	*GH1:* 1, *FECH:* 17, *EYA1:* 66, *LPL:* 100, *CAPN3*ex10: 91, *COL1A1:* 100, *CAPN3*ex17: 0, *PKHD1:* 99

```
pos3C=[100,41,81,100,100,100]#n=6
pos3Csub=[1,17,66,100,91,100,0,99]#n=8
KruskalResult(statistic=2.184722222222221, pvalue=0.13938620946554242)
Ttest_indResult(statistic=1.4712477438172564, pvalue=0.16933922490210684)
```

3. *Include PKHD normal 3'ss PSI 0 and exclude COL1A1 (N=13)*

	PSI
-3C	*HEXA:* 100, *COL1A2:* 41, *CLCN2:* 81, *LAMA2:* 100, *NEU1:* 100, *COL6A2:* 100, *PKHD1:* 0
-3Csub	*GH1:* 1, *FECH:* 17, *EYA1:* 66, *LPL:* 100, *CAPN3*ex10: 91, *CAPN3*ex17: 0,

```
pos3C=[100,41,81,100,100,100,0]#n=7
pos3Csub=[1,17,66,100,91,0]#n=6
KruskalResult(statistic=1.5647646813827558, pvalue=0.21096894075885705)
Ttest_indResult(statistic=1.2080619908622303, pvalue=0.2546947882170792)
```

4. *Include PKHD cryptic 3'ss PSI 99 and exclude COL1A1 (N=13)*

	PSI
-3C	*HEXA:* 100, *COL1A2:* 41, *CLCN2:* 81, *LAMA2:* 100, *NEU1:* 100, *COL6A2:* 100
-3Csub	*GH1:* 1, *FECH:* 17, *EYA1:* 66, *LPL:* 100, *CAPN3*ex10: 91, *CAPN3*ex17: 0, *PKHD1:* 99

```
pos3C=[100,41,81,100,100,100]#n=6
pos3Csub=[1,17,66,100,91,0,99]#n=7
KruskalResult(statistic=3.1096345514950166, pvalue=0.0778304219058807)
Ttest_indResult(statistic=1.6833419314017677, pvalue=0.12573231405797983)
```

2.Include COL1A1 and PKHD cryptic 3'ss PSI 99 (N=14)

```
## Calculations from lists (unequal sizes are acceptable)
pos3C=c(100,41,81,100,100,100)#n=6
pos3Csub=c(1,17,66,100,91,100,0,99)#n=8

> kruskal.test(list(pos3C,pos3Csub))#uses vectors without NaNs

      Kruskal-Wallis rank sum test

data:  list(pos3C, pos3Csub)
Kruskal-Wallis chi-squared = 2.1847, df = 1, p-value = 0.1394

> shapiro.test(pos3Csub)$p.value
[1] 0.02263669
> shapiro.test(pos3C)$p.value
[1] 0.002399249
> sd(pos3Csub)
[1] 45.73761
> sd(pos3C)
[1] 23.78235
> (sd(pos3Csub))^2
[1] 2091.929
> (sd(pos3C))^2
[1] 565.6

> t.test(pos3C,pos3Csub,var.equal = FALSE)#uses vectors without NaNs

      Welch Two Sample t-test

data:  pos3C and pos3Csub
t = 1.4712, df = 10.962, p-value = 0.1693
alternative hypothesis: true difference in means is not equal to 0
95 percent confidence interval:
 -13.78145  69.28145
sample estimates:
mean of x mean of y
    87.00     59.25

###Dataframe (requires NaNs added to smaller groups to make sizes equal)
pos3C=c(100,41,81,100,100,100,NaN,NaN)#n=6
pos3Csub=c(1,17,66,100,91,100,0,99)#n=8
U5_table=data.frame(cbind(pos3C,pos3Csub))
U5_table_stacked=stack(U5_table)
#kruskal.test and t.test produce the same result as with lists without NaNs
kruskal.test(values~ind,U5_table_stacked)#uses df with NaNs

> oneway.test(values~ind,U5_table_stacked, var.equal = FALSE)#uses df with NaNs

      One-way analysis of means (not assuming equal variances)

data:  values and ind
F = 2.1646, num df = 1.000, denom df = 10.962, p-value = 0.1693
```

4.Include PKHD cryptic 3’ss PSI 99 and exclude COL1A1 (N=13)

```
### Calculations from lists (unequal sizes are acceptable)
pos3C=c(100,41,81,100,100,100)#n=6
pos3Csub=c(1,17,66,100,91,0,99) #n=7

> kruskal.test(list(pos3C,pos3Csub))#uses vectors without NaNs

	Kruskal-Wallis rank sum test

data:  list(pos3C, pos3Csub)
Kruskal-Wallis chi-squared = 3.1096, df = 1, p-value = 0.07783

> shapiro.test(pos3Csub)$p.value
[1] 0.06937585
> shapiro.test(pos3C)$p.value
[1] 0.002399249
> sd(pos3Csub)
[1] 46.08997
> sd(pos3C)
[1] 23.78235
> (sd(pos3Csub))^2
[1] 2124.286
> (sd(pos3C))^2
[1] 565.6

> t.test(pos3C,pos3Csub,var.equal = FALSE)#uses vectors without NaNs

	Welch Two Sample t-test

data:  pos3C and pos3Csub
t = 1.6833, df = 9.237, p-value = 0.1257
alternative hypothesis: true difference in means is not equal to 0
95 percent confidence interval:
 -11.36769  78.51054
sample estimates:
mean of x mean of y
 87.00000  53.42857

###Dataframe (requires NaNs added to smaller groups to make sizes equal)#For
pos3C=c(100,41,81,100,100,100,NaN)#n=6
pos3Csub=c(1,17,66,100,91,0,99) #n=7
U5_table=data.frame(cbind(pos3C,pos3Csub))
U5_table_stacked=stack(U5_table)
#kruskal.test and t.test produce the same result as with lists without NaNs
kruskal.test(values~ind,U5_table_stacked)#uses df with NaNs
t.test(values~ind,U5_table_stacked, var.equal = FALSE)#uses df with NaNs

> oneway.test(values~ind,U5_table_stacked, var.equal = FALSE)#uses df with NaNs

	One-way analysis of means (not assuming equal variances)

data:  values and ind
F = 2.8336, num df = 1.000, denom df = 9.237, p-value = 0.1257
```

Boxplots for PSI dependent on +2C/+3G, substitute *A* or *T* and -3C

1. *Include COL1A1 and PKHD normal 3'ss PSI 0 (N=14)*

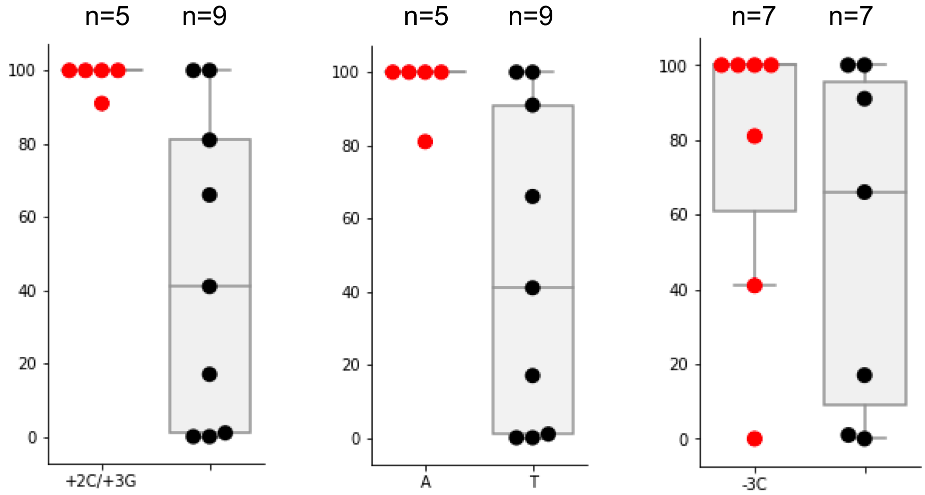

2. *Include COL1A1 and PKHD cryptic 3'ss PSI 99 (N=14)*

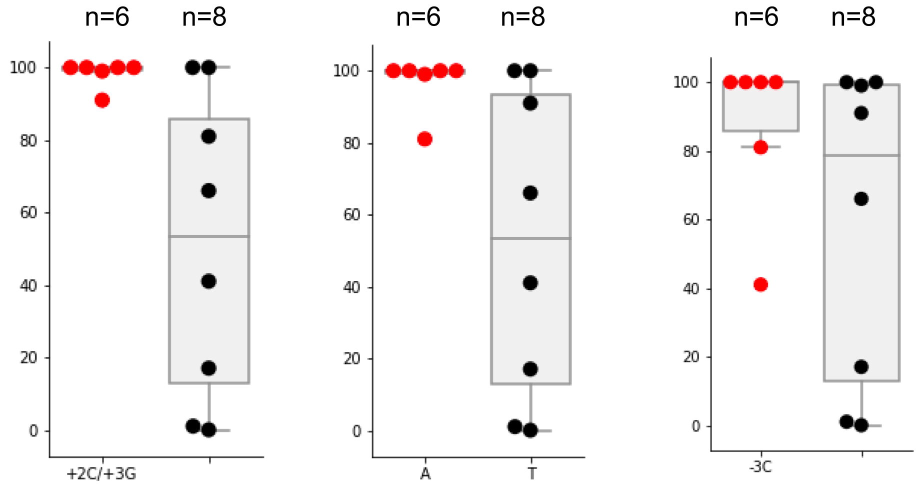

3. *Include PKHD normal 3'ss PSI 0 and exclude COL1A1 (N=13)*

4. *Include PKHD cryptic 3'ss PSI 99 and exclude COL1A1 (N=13)*

Spearman's correlation for PPS length

Gene	Ex	PSI	PPT Stretch (PPS)
GH1	3	1	10
FECH	9	17	6
EYA1	10	66	5
LPL	5	100	14
HEXA	13	100	13
PKHD1	25	99	6
		**0**	3
COL1A2	37	41	5
CLCN2	19	81	4
LAMA2	24	100	9
CAPN3	10	91	10
CAPN3	17	0	9
NEU1	2	100	14
COL6A2	8	100	15
COL1A1	23	100	16

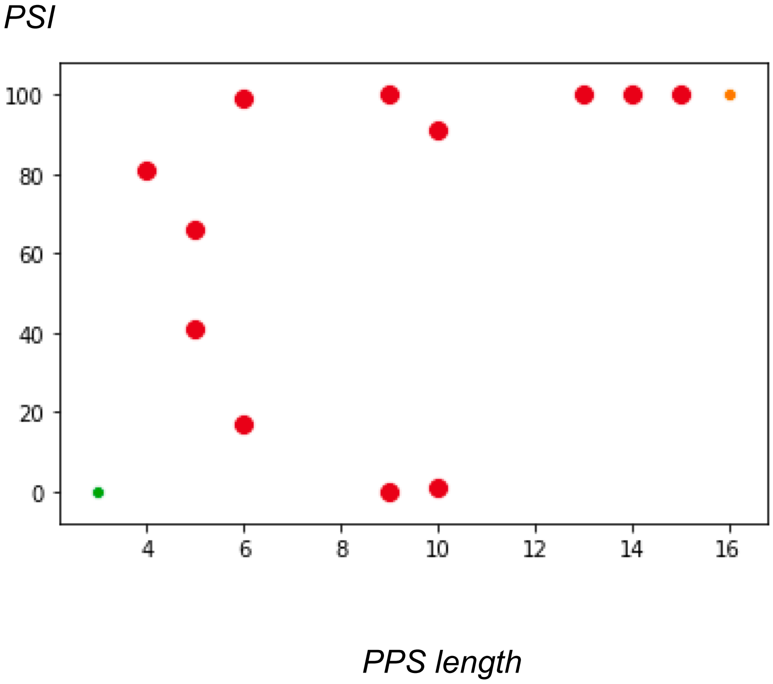

1. *Include COL1A1 and PKHD normal 3'ss PSI 0 (N=14)*

```
X=c(10,6, 5, 14, 13, 3,5, 4, 9,  10,9,14, 15, 16)#PPS length
Y=c(1, 17,66,100,100,0,41,81,100,91,0,100,100,100)#PSI
SpearmanrResult(correlation=0.7108258237527484, pvalue=0.004375165814040274)
```

2. *Include COL1A1 and PKHD cryptic 3'ss PSI 99 (N=14)*

```
X=c(10,6, 5, 14, 13, 6, 5, 4, 9,  10,9,14, 15, 16)#PPS length
Y=c(1, 17,66,100,100,99,41,81,100,91,0,100,100,100)#PSI
SpearmanrResult(correlation=0.6360104238605363, pvalue=0.014485699211541149)
```

3. *Include PKHD normal 3'ss PSI 0 and exclude COL1A1 (N=13)*

```
X=c(10,6, 5, 14, 13, 3,5, 4, 9,  10,9,14, 15) #PPS length
Y=c(1, 17,66,100,100,0,41,81,100,91,0,100,100) #PSI
SpearmanrResult(correlation=0.6701817559482136, pvalue=0.012192348615293494)
```

4. *Include PKHD cryptic 3'ss PSI 99 and exclude COL1A1 (N=13)*

```
X=c(10,6, 5, 14, 13, 6, 5, 4, 9,  10,9,14, 15) #PPS length
Y=c(1, 17,66,100,100,99,41,81,100,91,0,100,100) #PSI
SpearmanrResult(correlation=0.5776564039581658, pvalue=0.03868651748677648)
```

Spearman's correlation for branchpoint matches

Gene	Ex	PSI	BP matches
GH1	3	1	1
FECH	9	17	4
EYA1	10	66	3
LPL	5	100	4
HEXA	13	100	2
PKHD1	25	99	3
		**0** •	3 •
COL1A2	37	41	3
CLCN2	19	81	4
LAMA2	24	100	4
CAPN3	10	91	2
CAPN3	17	0	4
NEU1	2	100	3
COL6A2	8	100	4
COL1A1	23	100 •	0 •

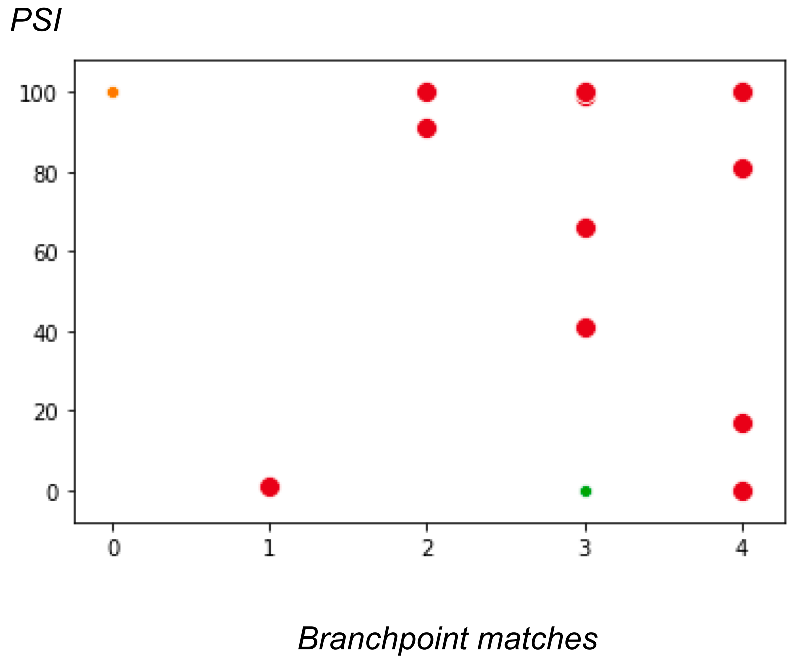

1. *Include COL1A1 and PKHD normal 3'ss PSI 0 (N=14)*
```
X=c(1,4, 3, 4,  2,  3,3, 4, 4,  2, 4,3,  4,  0)
Y=c(1,17,66,100,100,0,41,81,100,91,0,100,100,100)
```
```
SpearmanrResult(correlation=-0.025364168730l4165, pvalue=0.9314119671811683)
```

2. *Include COL1A1 and PKHD cryptic 3'ss PSI 99 (N=14)*
```
X=c(1,4, 3, 4,  2,  3, 3, 4, 4,  2, 4,3,  4,  0)
Y=c(1,17,66,100,100,99,41,81,100,91,0,100,100,100)
```
```
SpearmanrResult(correlation=-0.04946153176891343, pvalue=0.8666504276289523)
```

3. *Include PKHD normal 3'ss PSI 0 and exclude COL1A1 (N=13)*
```
X=c(1,4, 3, 4,  2,  3,3, 4, 4,  2, 4,3,  4)
Y=c(1,17,66,100,100,0,41,81,100,91,0,100,100)
```
```
SpearmanrResult(correlation=0.12414328599550853, pvalue=0.6861588126520046)
```

4. *Include PKHD cryptic 3'ss PSI 99 and exclude COL1A1 (N=13)*
```
X=c(1,4, 3, 4,  2,  3, 3, 4, 4,  2, 4,3,  4)
Y=c(1,17,66,100,100,99,41,81,100,91,0,100,100)
```
```
SpearmanrResult(correlation=0.09372790559206708, pvalue=0.7607010691102144)
```

Scatterplots for PSI dependent on PPS length and branchpoint matches

1. *Include COL1A1 and PKHD normal 3'ss PSI 0 (N=14)*

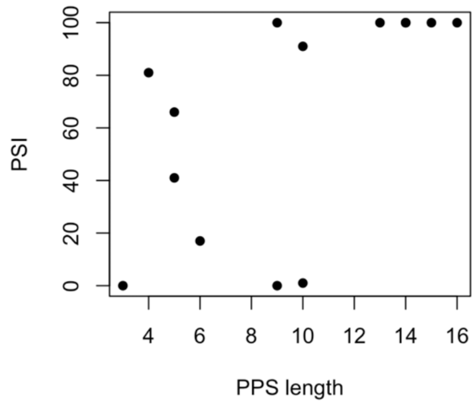

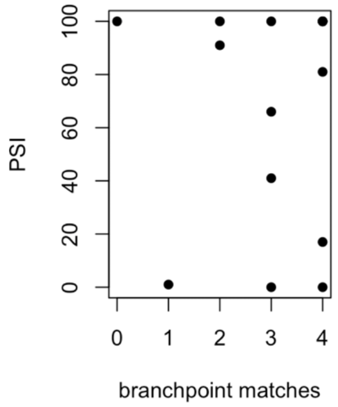

2. *Include COL1A1 and PKHD cryptic 3'ss PSI 99 (N=14)*

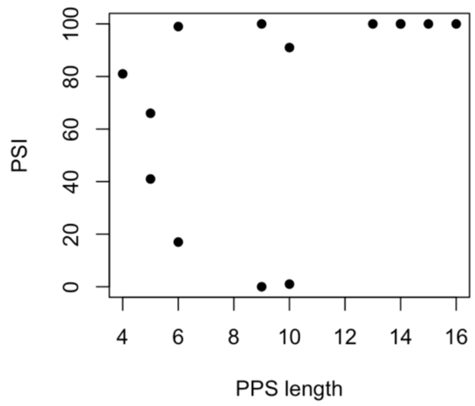

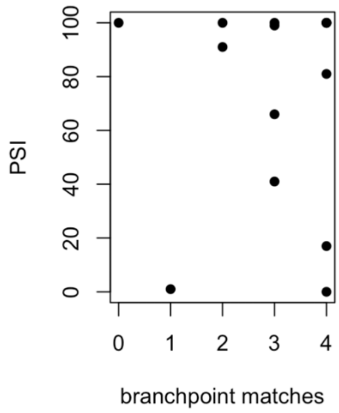

3. *Include PKHD normal 3'ss PSI 0 and exclude COL1A1 (N=13)*

4. *Include PKHD cryptic 3'ss PSI 99 and exclude COL1A1 (N=13)*

5. *All-in-one*

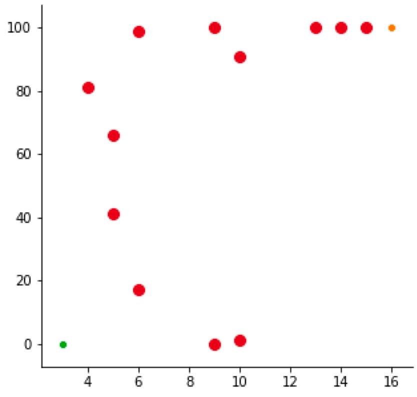

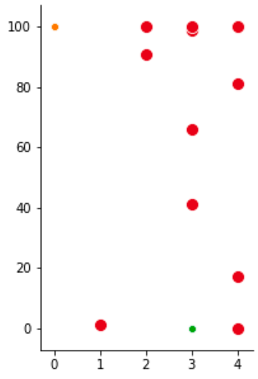

Section S5: Future work: Molecular cell biology testing of the U5 hypothesis

Below, we include suitable human mutations for the proof-of-principle laboratory testing:

1. A **Becker muscular dystrophy** patient with c9563+5G>C mutation in intron 65 (Juan-Mateu et al., 2013) has a dystrophin deficiency due to the activation of two alternative cryptic 5' splice sites. Wild-type dystrophin exon 65 unusually ends with a C (c9563), while the cryptic sites both have Gs for the exon-end. We can attempt to restore normal splicing in a minigene construct in cultured cells with a second compensatory mutation of exon 65 -1C>G (c9563C>G) to match the wild type U5 39C. Reciprocal experiment, a swap of G and C between the interacting RNAs, can involve modified U5 39C>G to match the wt exon 65 -1C (**Figure S5)**. U5 should be checked out independently and in combination with the matching U1 9C>G.

2. A rare A>G substitution in the **coagulation factor V** gene (*F5-Texas*, Vincent et al., 2013), defines an end of an exon and activates splicing of an alternative intron, causing a bleeding phenotype. Interestingly, a wt *F5-short* was later identified in normal individuals as a rare splice isoform that regulates anticoagulation. *F5-Texas* mutant guanine at the end of the exon preceding the alternative intron activates splicing by the factor of 10^2 compared to the wt A in this position. Again, an experiment with a minigene or full length F5 cDNA construct can serve to swap the G and C between the interacting RNAs: F5 alternative intron preceded by -1A>C mutation and modified U5 39C>G. Again, modified U1 9C>G can be considered in parallel (**Figure S6**). The future clinical application involves regulation of anticoagulation for the prevention of common venous thrombosis by targeting F5 alternative intron with a full set of complementary snRNAs.

Follow up of the previous experiments that have already determined that a change in the exon sequence at the splice junction can suppress a different splicing mutation:

3. Mutation study of the ***IKBKAP*** gene (Introduction; Carmel et al., 2004) indicate that substitution of a rare exon-end A for G fully restores normal splicing, blocked by +6T>C mutation. Some restoration of normal splicing is achieved using U1 9C>U modification to match the wild-type end of exon A. In case of U5 snRNA a change for one more U in the uracil-rich Loop1 might be not very useful, so, we suggest starting with U5 39C>G modification combined with the exon-end A for C substitution to introduce the swap G=C pair. U1 9C>G can be tried in parallel.

4. The study of the mutant **ATR** gene (Introduction; Scalet et al., 2017) can be adapted to change the end of exon 9 A>C co-transfected with modified U5 C39>G gene to check if the swap G=C pair will also correct spicing independently or in combination with the matching U1 9C>G snRNA. At the wt 3'ss exon 9 conveniently starts with a C, which will match U5 C38>G modification.

5. The compensatory mutation study of the ***FAH*** c1062+5G>A allele (Discussion above; Scalet et al., 2018) can be followed by testing the effect of U5 37U>C modification to create U5 C_{37}=G_{-2} pair with wt exon 12 in combination with the modified U1 version from the previous study.

Follow up of an exon-end G>A mutation:

6. Finally, in a very recent study (Breuel et al., 2019) from Prof Neidhardt laboratory, who pioneered combining both modified U1 and U6 for splicing correction (Schmid et al., 2013), Breuel and colleagues use adapted U1 snRNA together with antisense oligos to correct the G>A substitution at the end of **BBS** exon 5. In order to verify if a combination of modified U1 and U5 will be effective to correct this mutation, we suggest first changing it in the minigene for G>C and co-transfecting with the matching U5 C39>G and U1 9C>G genes (alternatively and combined).

Testing U2 snRNA interaction with the end of the intron (the proposed U2 G_{31}=C_{-3} pair) is more straightforward and any human intron can do, however the **Fas/CD95** intron 5 (Corrionero et al, 2011) is an excellent study to follow. The inclusion of Fas receptor exon 6 is an apoptotic switch, which is disabled by -3C>G mutation in intron 5 leading to accumulation of T-cells in autoimmune limphoproliferative syndrome (ALPS). We can attempt to rescue normal splicing of intron 5 in a minigene construct using modified U2 31G>C (**Figure S7**).

Therapeutic application for every case outlined above will involve the assessment of the efficacy of a full set of complementary snRNAs (U1, U6, U5 and U2) specific to the target intron and splice junction and with a swapped U2/U6 helix II, which will decrease the intermixing with the endogenous snRNAs.

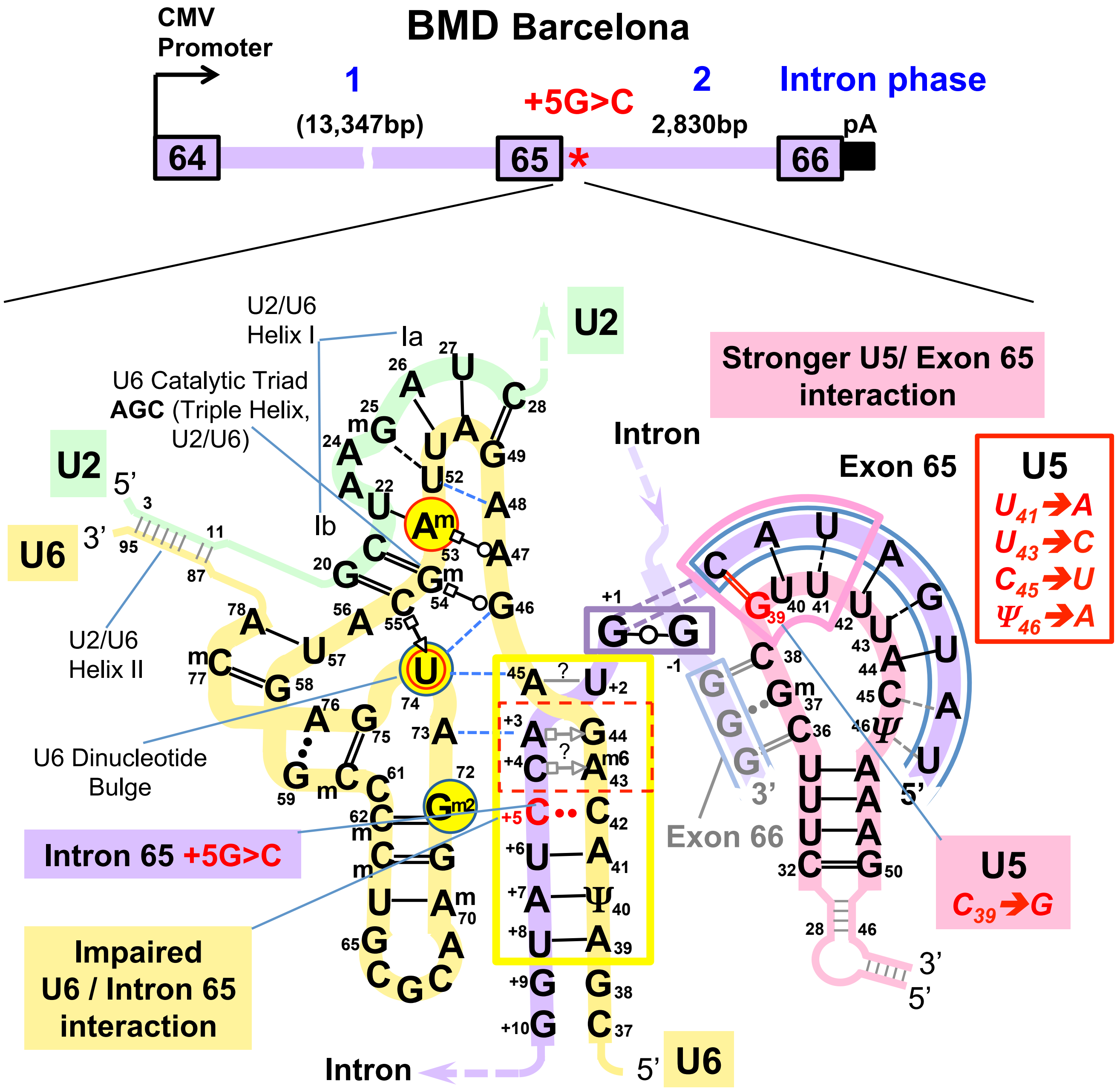

Figure S5| Study design for the correction of the dystrophin gene splicing mutation c9563+5G>C in intron 65 from a Becker muscular dystrophy patient reported by Juan-Mateu et al., 2013. The U5 snRNA modifications, matching wt exon 65 are shown here, as we are specifically interested if U5 can compensate for the loss of the most conserved Watson-Crick pair at the U6 binding site. For therapeutic purpose all spliceosomal snRNAs can be modified to increase complementarity to the dystrophin intron 65. U1 and U6 are obvious candidates for modifications matching the mutant +5C.

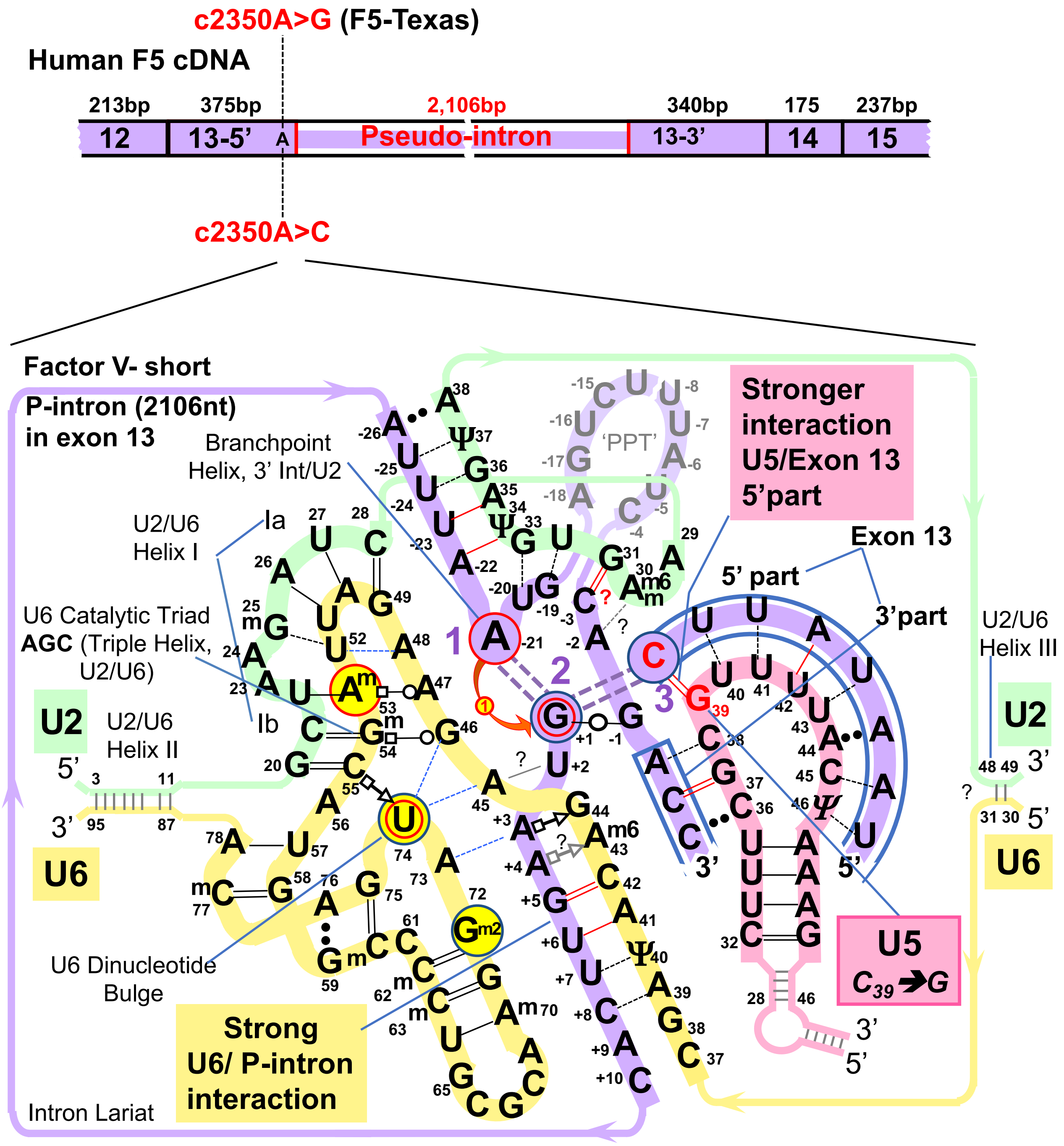

Figure S6| Study design targeting the alternative intron (pseudo-intron) splicing of coagulation F5. Normally, 1% of the circulating F5 protein is the short isoform, lacking the part that corresponds to the alternative intron in exon 13. F5-Texas mutation (Vincent et al., 2013) leads to exclusive production of the F5-short isoform and bleeding disorder. To explore U5 snRNA base pairing with the conserved exon-end guanine we can try to introduce a co-variant pair and swap the G and C between the interacting RNAs: F5 c2350A>C mutation and modified U5 39C>G. Modified U1 9C>G can be considered in parallel. U1 9C>U and a full set of snRNAs complementary to the wt alternative intron might help to reduce coagulation in patients with thrombosis.

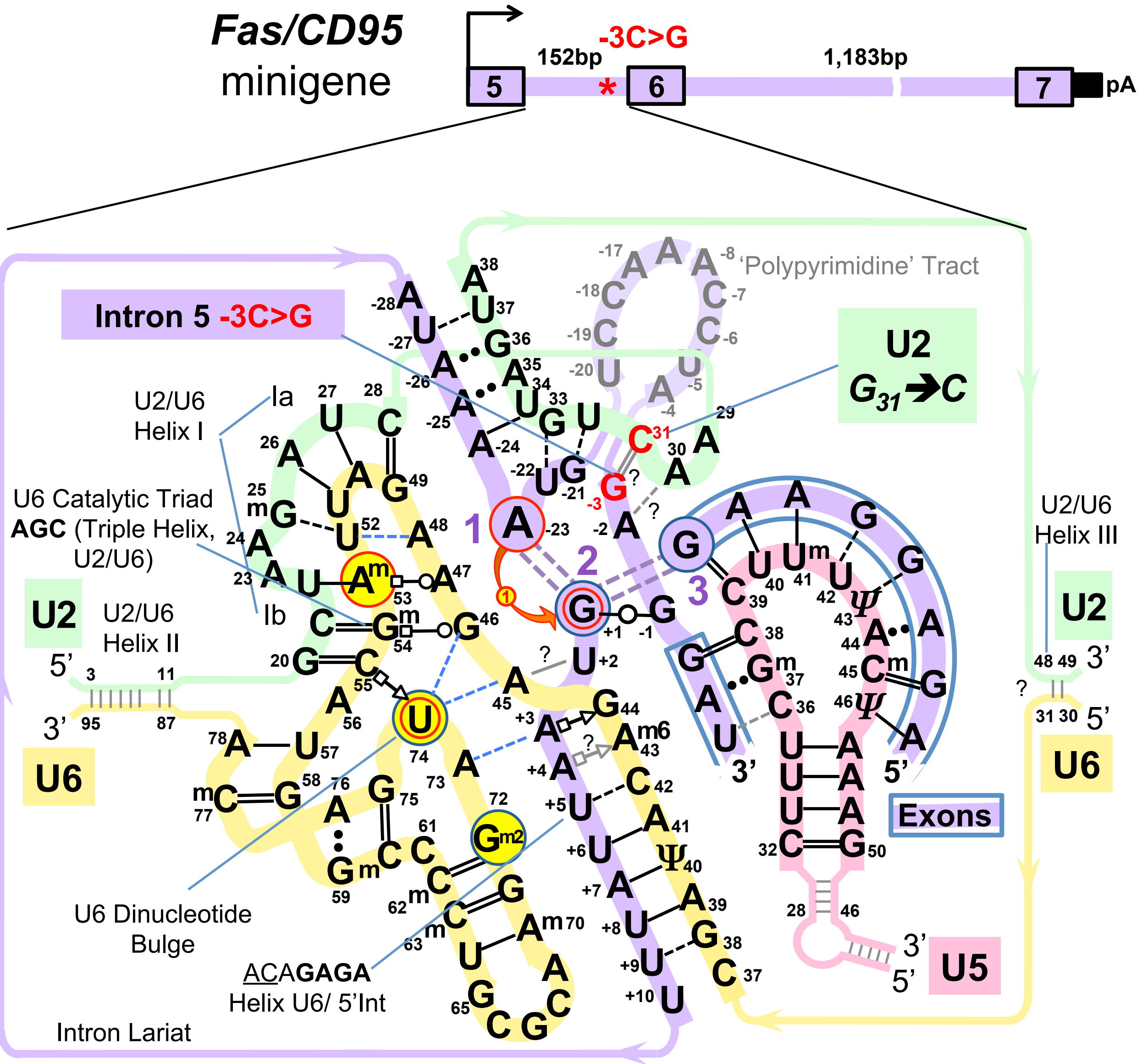

Figure S7| Testing U2 snRNA interaction with the end of the intron (the proposed U2 G_{31}=C_{-3} pair) following the study of Corrionero et al., 2011 on Fas/CD95 intron 5. The inclusion of Fas receptor exon 6 is an apoptotic switch, which is disabled by -3C>G mutation in intron 5 leading to accumulation of T-cells in autoimmune limphoproliferative syndrome (ALPS). We can attempt to rescue normal splicing of intron 5 in a minigene construct using modified U2 31G>C.

Supplementary References

Adachi H, Yu YT. (2014) Insight into the mechanisms and functions of spliceosomal snRNA pseudouridylation World J Biol Chem. 5, 398–408. Mechanism of snRNA pseudouridylation

Anderson K, Moore MJ. (1997) Bimolecular exon ligation by the human spliceosome. Science. 276,1712-6. AdML pre-mRNA substrate

Bessonov S, Anokhina M, Will CL, Urlaub H, Lührmann R. (2008) Isolation of an active step I spliceosome and composition of its RNP core. Nature. 452, 846-50. PM5 pre-mRNA substrate

Burge,C.,B., Padgett,R.,A. and Sharp,P.,A. (1998) Evolutionary fates and origins of U12-type introns. Mol. Cell 2, 773-785. Minor (U12) introns

Burke MF, Logan MK, Hebert MD (2018) Identification of additional regulatory RNPs that impact rRNA and U6 snRNA methylation. Biol Open. 7, bio036095. Mechanism of U6 snRNA methylation

Charenton C, Wilkinson ME, Nagai K. (2019) Mechanism of 5' splice site transfer for human spliceosome activation. Science. 364,362-367. Human Pre-B spliceosome complex, Cryo-EM

Chen JY, Stands L, Staley JP, Jackups RR Jr, Latus LJ, Chang TH. 2001 Specific alterations of U1-C protein or U1 small nuclear RNA can eliminate the requirement of Prp28p, an essential DEAD box splicing factor. *Mol Cell* **7**: 227-32. Mechanism of Prp28-driven disassociation of U1 snRNA from the 5'ss

Ciavarella, J., Perea, W., & Greenbaum, N. L. (2020). Topology of the U12-U6atac snRNA Complex of the Minor Spliceosome and Binding by NTC-Related Protein RBM22. ACS omega, 5, 23549–23558. U12-U6atac duplex, minor spliceosome

Coimbatore Narayanan B, Westbrook J, Ghosh S, Petrov AI, Sweeney B, Zirbel CL, Leontis NB, Berman HM. (2014) The Nucleic Acid Database: new features and capabilities. Nucleic Acids Res. 42, D114-22. RNA base pair catalogue http://ndbserver.rutgers.edu/ndbmodule/services/BPCatalog/bpCatalog.html Westhof geometric classification of RNA base pairs updated

Crotti L.B., Horowitz D.S. (2009) Exon sequences at the splice junctions affect splicing fidelity and alternative splicing. Proc. Natl. Acad. Sci. U S A 106, 18954-18959. Exon-ends mutagenesis in yeast

Deryusheva S and Gall JG (2018) Orchestrated positioning of post-transcriptional modifications at the branch point recognition region of U2 snRNA. RNA. 24, 30–42 Mechanism of U2 snRNA modifications

Dietrich RC, Fuller JD, Padgett RA (2005) A mutational analysis of U12-dependent splice site dinucleotides. RNA. 11, 1430-40. Minor (U12) introns

Dietrich,R.,C., Incorvaia,R. and Padgett,R.,A. (1997) Terminal intron dinucleotide sequences do not distinguish between U2- and U12-dependent introns. Mol. Cell. 1, 151-160. Minor (U12) introns

Dietrich RC, Peris MJ, Seyboldt AS, Padgett RA. (2001) Role of the 3' splice site in U12-dependent intron splicing. Mol Cell Biol. 21, 1942-52. Minor (U12) introns

Dlakić M., Mushegian A. (2011) Prp8, the pivotal protein of the spliceosomal catalytic center, evolved from a retroelement-encoded reverse transcriptase. RNA 17, 799-808. Spliceosome Prp8 homologous to Group II intron encoded protein (IEP)

Fabrizio P., Abelson J. (1990) Two domains of yeast U6 small nuclear RNA required for both steps of nuclear precursor messenger RNA splicing. Science 250, 404-409. *S. cerevisiae* U6 A_{51}-U_{+2} crosslinks

Fica S.M., Oubridge C., Galej W.P., Wilkinson M.E., Bai X.C., Newman A.J., Nagai K. (2017) Structure of a spliceosome remodelled for exon ligation. Nature 542, 377-380. *S. cerevisiae* **C*** complex, CryoEM

Fica SM, Oubridge C, Wilkinson ME, Newman AJ, Nagai K. (2019) A human postcatalytic spliceosome structure reveals essential roles of metazoan factors for exon ligation. Science. 363,710-714. Human **P** complex, CryoEM

Galej W.P., Oubridge C., Newman A.J., Nagai K. (2013) Crystal structure of Prp8 reveals active site cavity of the spliceosome. Nature 493, 638-643. Prp8 cavity has the dimensions of Group **II** intron catalytic core

Ge J and Yu YT (2013) RNA pseudouridylation: new insights into an old modification. Trends Biochem Sci. 38, 210–218. Mechanism of RNA pseudouridylation

Grainger R.J., Beggs J.D. (2005) Prp8 protein: at the heart of the spliceosome. RNA 11, 533-557. Prp8 Review.

Hall,S.L., Padgett,R.,A. (1994) Conserved sequences in a class of rare eukaryotic nuclear introns with non-consensus splice sites. J. Mol. Biol. 239, 357-65. Minor (U12) introns

Haselbach D., Komarov I., Agafonov D.E., Hartmuth K., Graf B., Dybkov O., Urlaub H., Kastner B., Lührmann R., Stark H. (2018) Structure and Conformational Dynamics of the Human Spliceosomal Bact Complex. Cell 172, 454-464.e11. Human **Bact** complex with MINX substrate, CryoEM

Hesselberth, J.R. (2013) Lives that introns lead after splicing. WIREs RNA, 4: 677-691. Metabolism of excised introns

Jády,B.,E. and Kiss,T. (2001) A small nucleolar guide RNA functions both in 2'-O-ribose methylation and pseudouridylation of the U5 spliceosomal RNA. EMBO J. 20, 541-551. Mechanism of U5 snRNA modifications

Karunatilaka KS and Rueda D (2014) Post-transcriptional modifications modulate conformational dynamics in human U2–U6 snRNA complex. RNA. 20, 16–23. The role of U2/U6 snRNA modifications

Kim C.H., Abelson J. (1996) Site-specific crosslinks of yeast U6 snRNA to the pre-mRNA near the 5' splice site. RNA 2, 995-1010. *Saccharomyces cerevisiae* U6 A_{51}-U_{+2} crosslinks

Kustatscher G, Grabowski P, Schrader TA, Passmore JB, Schrader M, Rappsilber J. (2019) Co-regulation map of the human proteome enables identification of protein functions. Nat Biotechnol 37, 1361–1371. Flexible and meaningful functional grouping of human proteins as opposed to functional classification

Leontis N.B., Westhof E. (2001) Geometric nomenclature and classification of RNA base pairs. RNA, 7, 499-512. Westhof geometric classification of RNA base pairs

Liu S., Li X., Zhang L., Jiang J., Hill R.C., Cui Y., Hansen K.C., Zhou Z.H., Zhao R. (2017) Structure of the yeast spliceosomal postcatalytic P complex. Science 358, 1278-1283. *S. cerevisiae* **P** complex, CryoEM **(Zhao Lab)**

Meier UT. (2017) RNA modification in Cajal bodies. RNA Biol. 4, 693–700. Review. Mechanism of pseudouridylation and 2’-O-methylation of snRNAs

Mládek A, Sharma P, Mitra A, Bhattacharyya D, Sponer J, Sponer JE. (2009) Trans Hoogsteen/sugar edge base pairing in RNA. Structures, energies, and stabilities from quantum chemical calculations. J Phys Chem B. 113, 1743-55. Energetics of U6 non-canonical base pairs with intron positions +3 and +4

Padgett RA, Konarska MM, Grabowski PJ, Hardy SF, Sharp PA. (1984) Lariat RNA's as intermediates and products in the splicing of messenger RNA precursors. Science 225, 898-903. MINX pre-mRNA substrate used for CryoEM of the human spliceosome

Pinotti M, Bernardi F, Dal Mas A, Pagani F. (2011) RNA-based therapeutic approaches for coagulation factor deficiencies. J Thromb Haemost. 9, 2143-52. Review. Partial suppression of +5G>A mutation in coagulation Factor VII gene by adapted U1 snRNA, snRNA therapeutics

Piovesan A, Antonaros F, Vitale L, Strippoli P, Pelleri MC, Caracausi M. (2019) Human protein-coding genes and gene feature statistics in 2019. BMC Res Notes. 12, 315. Summary of exon/intron structure of human protein-coding genes

Plaschka C, Lin PC, Nagai K. Structure of a pre-catalytic spliceosome. (2017) Nature. 546, 617-621. *Saccharomyces cerevisiae* **B** complex, CryoEM

Roithová A, Staněk D. (2019) Analysis of Spliceosomal snRNA Localization in Human Hela Cells Using Microinjection. J Vis Exp. 150, e59797. doi: 10.3791/59797. Method for *in vitro* transcription of U2 snRNA

Schneider M., Will C.L., Anokhina M., Tazi J., Urlaub H., Lührmann R. (2010) Exon definition complexes contain the tri-snRNP and can be directly converted into B-like precatalytic splicing complexes. Mol. Cell, 38**,** 223-235. Exon definition complexes

Sharp PA, Burge CB. Classification of introns: U2-type or U12-type. (1997) Cell. Dec 26;91(7):875-9. Splice site conservation and alternative spliceosomes

Solnick D. (1985) Trans splicing of mRNA precursors. Cell, 42, 157-164. Adeno pre-mRNA substrate

Szkukalek,A., Myslinski,E., Mougin,A., Luhrmann,R. and Branlant,C. (1995) Phylogenetic conservation of modified nucleotides in the terminal loop 1 of the spliceosomal U5 snRNA. Biochimie, 77, 16-21 Conservation of U5 Loop1 modifications

Teigelkamp S., Newman A.J., Beggs J.D. (1995) Extensive interactions of PRP8 protein with the 5' and 3' splice sites during splicing suggest a role in stabilization of exon alignment by U5 snRNA. EMBO J. 14, 2602-2612. Exon sequences used for *S.cerevisiae* U5 Loop1 crosslinking (Newman et al, 2015 - main text References)

Valadkhan S., Jaladat Y. (2010) The spliceosomal proteome: at the heart of the largest cellular ribonucleoprotein machine. Proteomics 10, 4128-4141. Spliceosomal proteins Review

Wan R., Yan C., Bai R., Huang G., Shi Y. (2016b) Structure of a yeast catalytic step I spliceosome at 3.4 Å resolution. Science 353, 895-904. *Saccharomyces cerevisiae* **C** complex, CryoEM

Wan R, Yan C, Bai R, Lei J, Shi Y. (2017) Structure of an Intron Lariat Spliceosome from Saccharomyces cerevisiae. Cell 171, 120-132.e12. *S.cerevisiae* **ILS** complex after exon disassociation, CryoEM

Wan R, Bai R, Yan C, Lei J, Shi Y. (2019) Structures of the Catalytically Activated Yeast Spliceosome Reveal the Mechanism of Branching. Cell. 177,339-351. *S.cerevisiae* **B*** complex CryoEM, *ACT1* and *UBC4* pre-mRNA *in vitro* assembly

Wilkinson M.E., Fica S.M., Galej W.P., Norman C.M., Newman A.J., Nagai K. (2017) Postcatalytic spliceosome structure reveals mechanism of 3'-splice site selection. Science 358, 1283-1288. *Saccharomyces cerevisiae* **P** complex, CryoEM

Wu G, Adachi H, Ge J, Stephenson D, Query CC, Yu YT. (2016) Pseudouridines in U2 snRNA stimulate the ATPase activity of Prp5 during spliceosome assembly. EMBO J. 35, 654-67. The role of U2 pseudouridines

Wu,G., Yu,A.T., Kantartzis,A. and Yu,Y.,T. (2011) Functions and mechanisms of spliceosomal small nuclear RNA pseudouridylation. Wiley Interdiscip. Rev. RNA 2, 571-581. The role of snRNA pseudouridylation

Wyatt J.R., Sontheimer E.J., Steitz J.A. (1992) Site-specific cross-linking of mammalian U5 snRNP to the 5' splice site before the first step of pre-mRNA splicing. Genes Dev. 6, 2542-2553. Human U5 - 5'exon crosslinks

Yan C, Hang J, Wan R, Huang M, Wong CC, Shi Y. (2015) Structure of a yeast spliceosome at 3.6-angstrom resolution. Science 349, 1182-91. *Schizosaccharomyces pombe* **ILS** complex, CryoEM

Yan C, Wan R, Bai R, Huang G, Shi Y. (2017) Structure of a yeast step II catalytically activated spliceosome. Science 355, 149-155. *Saccharomyces cerevisiae* **C*** complex, CryoEM

Yan C., Wan R., Shi Y. (2019) Molecular Mechanisms of pre-mRNA Splicing through Structural Biology of the Spliceosome. Cold Spring Harb. Perspect. Biol. 11. pii: a032409. Review of CryoEM studies

Zhang X, Yan C, Zhan X, Li L, Lei J, Shi Y. (2018) Structure of the human activated spliceosome in three conformational states. Cell Res 28:307-322. MINX15 substrate in human **Bact** complex; **refers to Bertram et al. 2017, however shows different binding register for B complex**, CryoEM

Zhan X., Yan C., Zhang X., Lei J., Shi Y. (2018[a]) Structure of a human catalytic step I spliceosome. Science 359, 537-545. MINX substrate in human **C** complex, CryoEM

Zhan X, Yan C, Zhang X, Lei J, Shi Y. (2018[b]) Structures of the human pre-catalytic spliceosome and its precursor spliceosome. Cell Res. 28,1129-1140. MINX substrate in human **preB** and **B** complexes, CryoEM

Zhao Y, Dunker W, Yu YT and Karijolich J (2018) The Role of Noncoding RNA Pseudouridylation in Nuclear Gene Expression Events. Front Bioeng Biotechnol. 6, 8. The Role of ncRNA pseudouridylation, Review

Zillmann M, Zapp ML, Berget SM. (1988) Gel electrophoretic isolation of splicing complexes containing U1 small nuclear ribonucleoprotein particles. Mol Cell Biol 8, 814-21. MINX pre-mRNA substrate used for CryoEM of the human spliceosome

Section S4: Results and Methods (continued)

Histograms of bootstrap difference for U5 bp geometry at exon junctions of +5Gsub and +5G introns

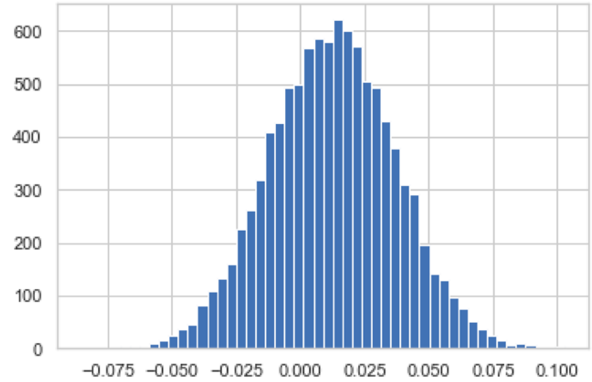

BD_5pr8_WC
P_H0_5pr8_WC 0.31

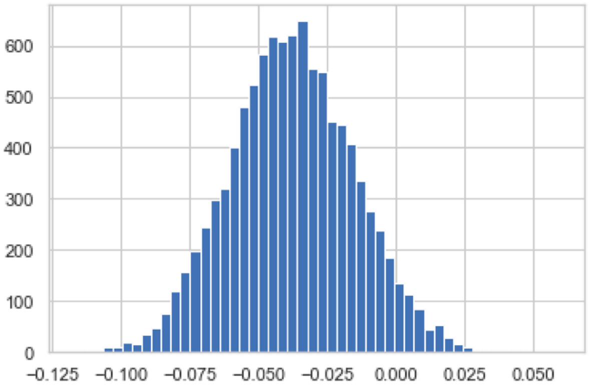

BD_5pr7_WC
P_H0_5pr7_WC 0.0486

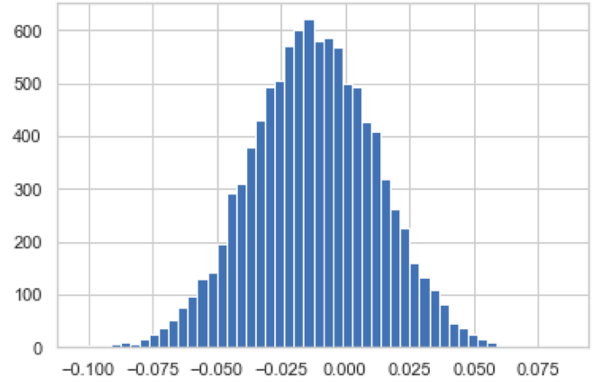

BD_5pr8_iso
P_H0_5pr8_iso 0.31

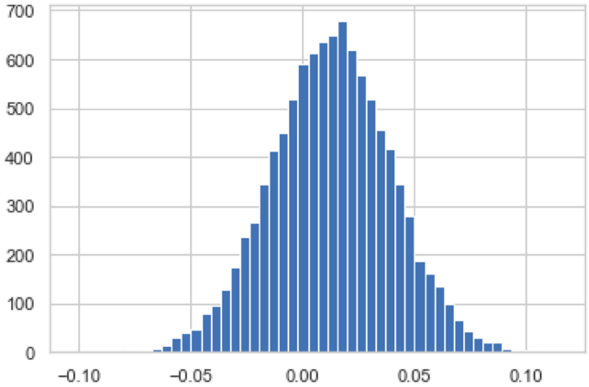

BD_5pr7_iso
P_H0_5pr7_iso 0.3104

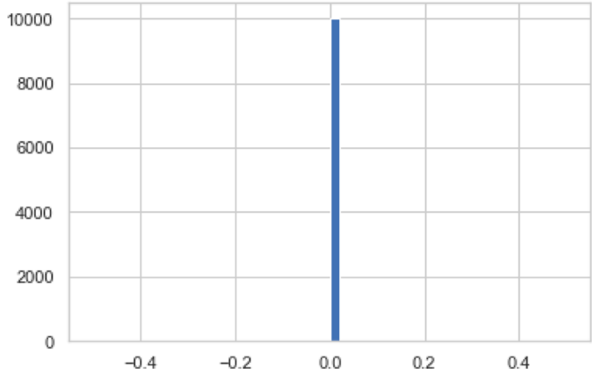

BD_5pr8_dif

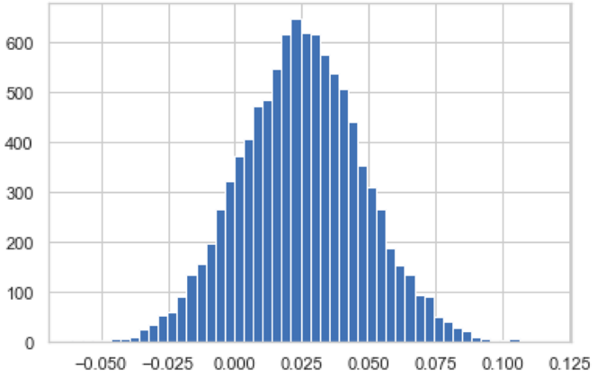

BD_5pr7_dif
P_H0_5pr7_dif 0.1414

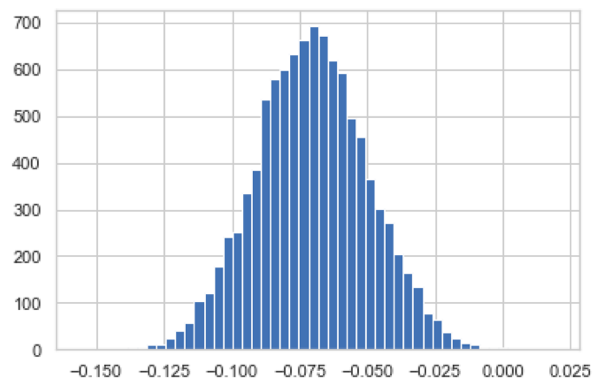

BD_5pr6_WC
P_H0_5pr6_WC 0.0007

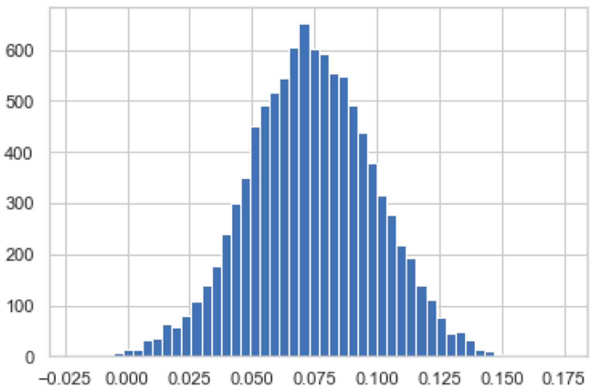

BD_5pr5_WC
P_H0_5pr5_WC 0.0025

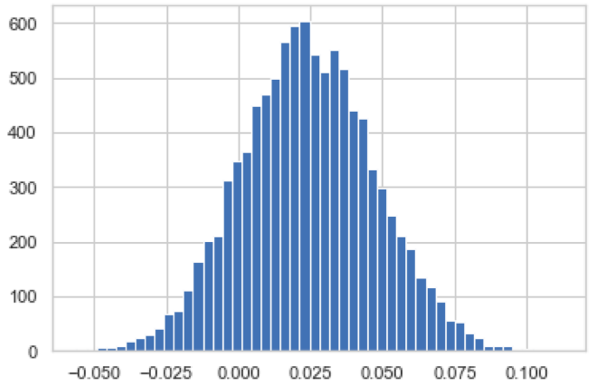

BD_5pr6_iso
P_H0_5pr6_iso 0.151

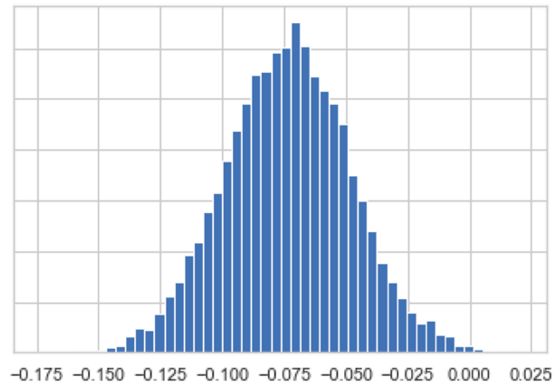

BD_5pr5_iso
P_H0_5pr5_iso 0.0025

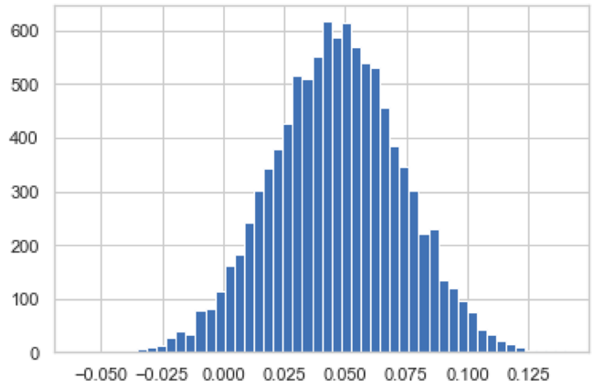

BD_5pr6_dif
P_H0_5pr6_dif 0.0394

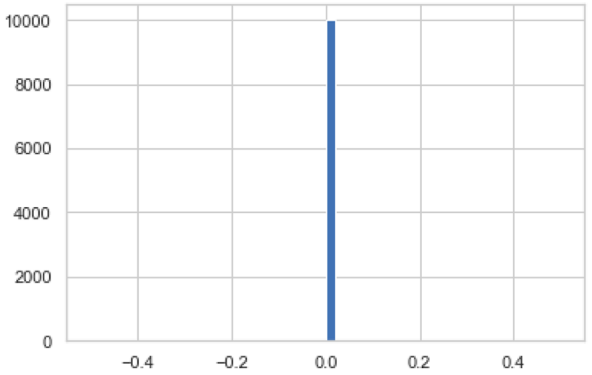

BD_5pr5_dif

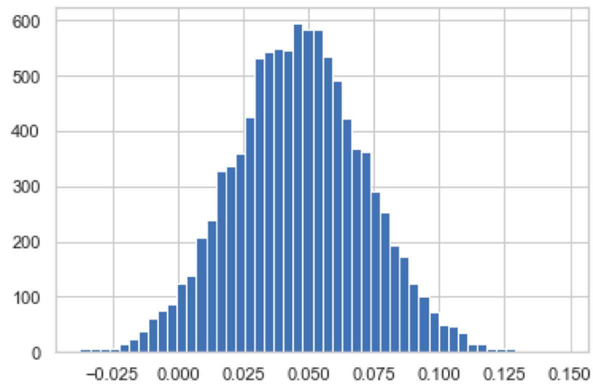

BD_5pr4_WC
P_H0_5pr4_WC 0.0336

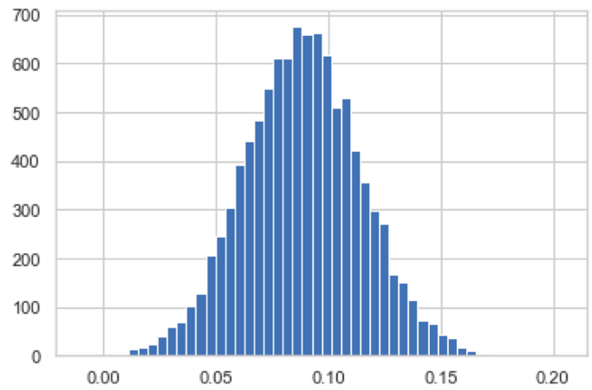

BD_5pr3_WC
P_H0_5pr3_WC 0.0003

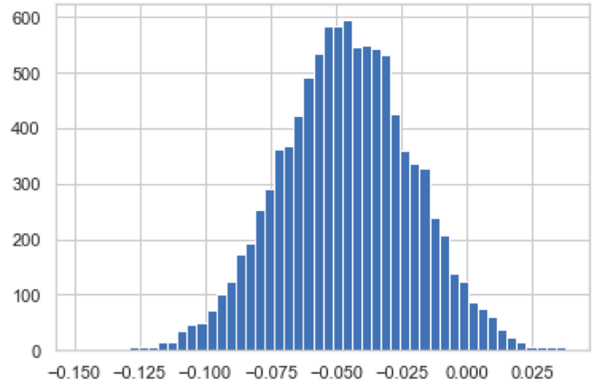

BD_5pr4_iso
P_H0_5pr4_iso 0.0336

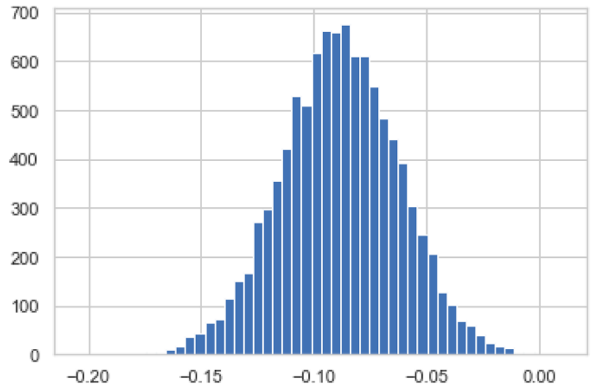

BD_5pr3_iso
P_H0_5pr3_iso 0.0003

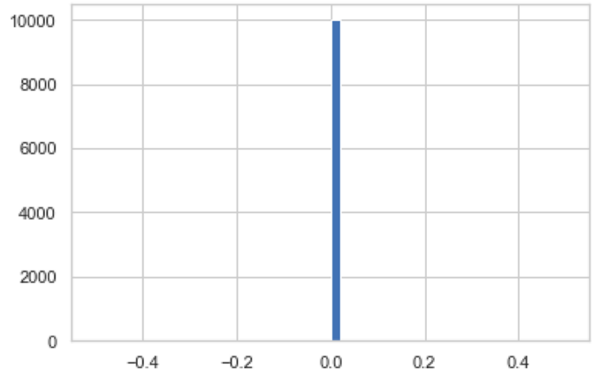

BD_5pr4_dif

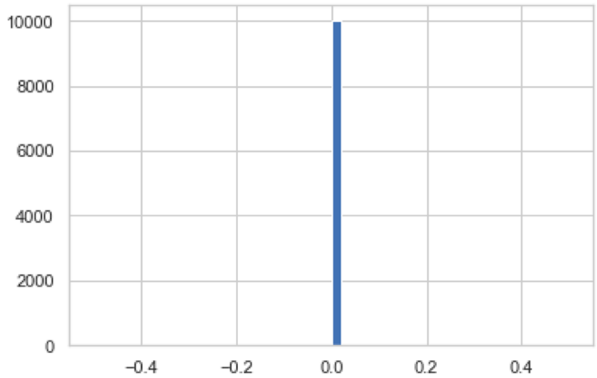

BD_5pr3_dif

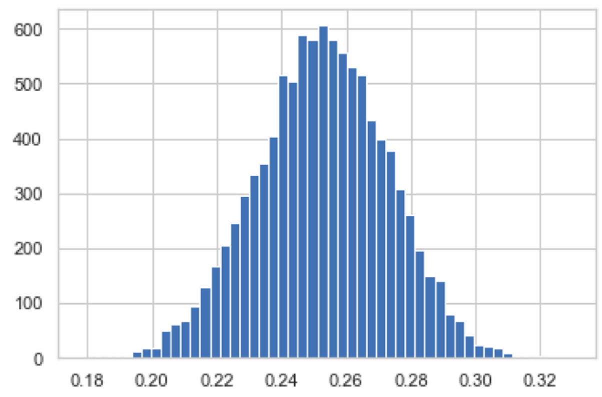

BD_5pr2_WC
P_H0_5pr2_WC 0.0

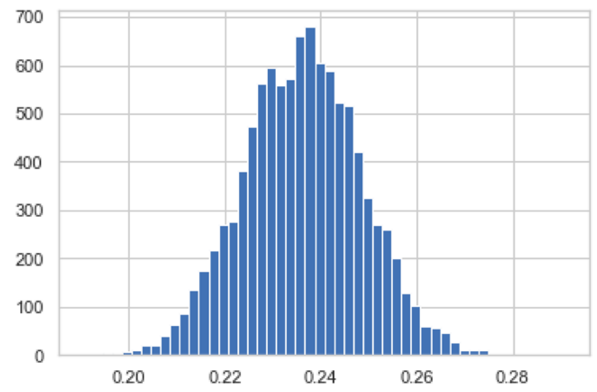

BD_5pr1_WC
P_H0_5pr1_WC 0.0

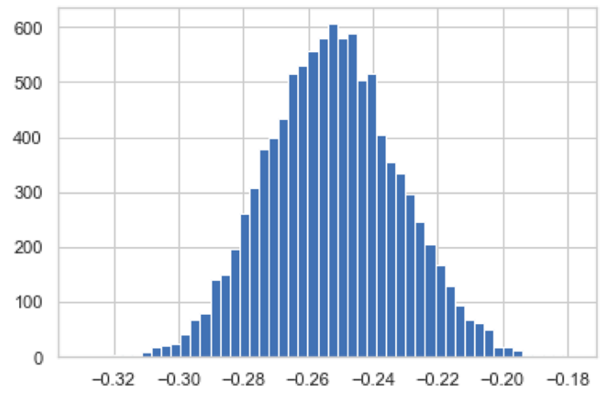

BD_5pr2_iso
P_H0_5pr2_iso 0.0

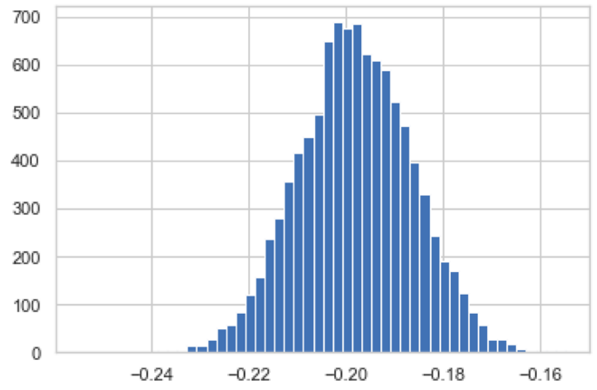

BD_5pr1_iso
P_H0_5pr1_iso 0.0

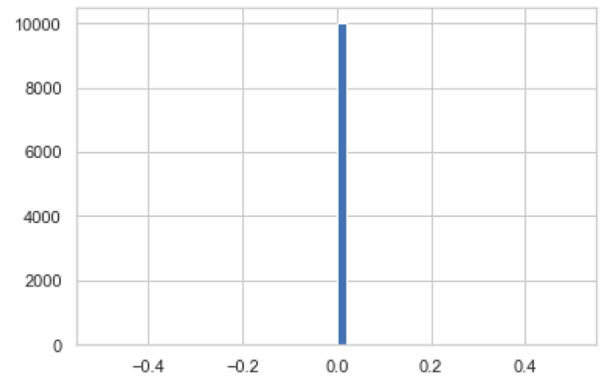

BD_5pr2_dif

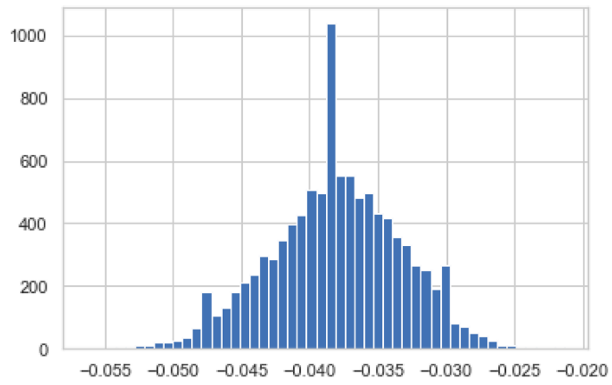

BD_5pr1_dif
P_H0_5pr1_dif 0.0

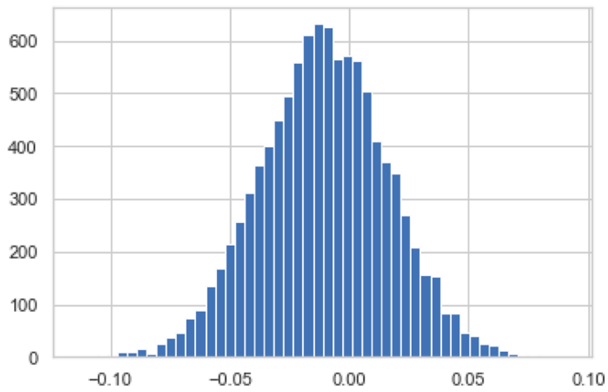

BD_3pr1_WC
P_H0_3pr1_WC 0.3473

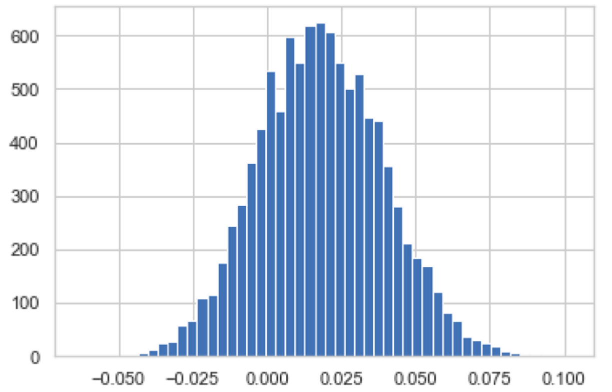

BD_3pr2_WC
P_H0_3pr2_WC 0.2026

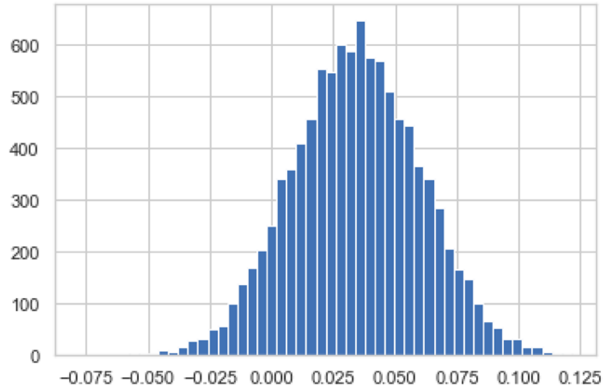

BD_3pr1_iso
P_H0_3pr1_iso 0.0951

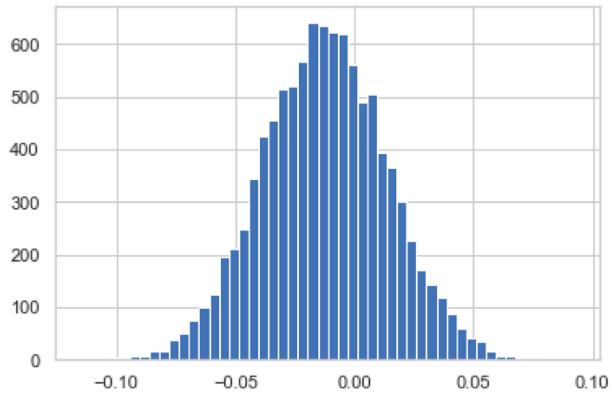

BD_3pr2_iso
P_H0_3pr2_iso 0.3149

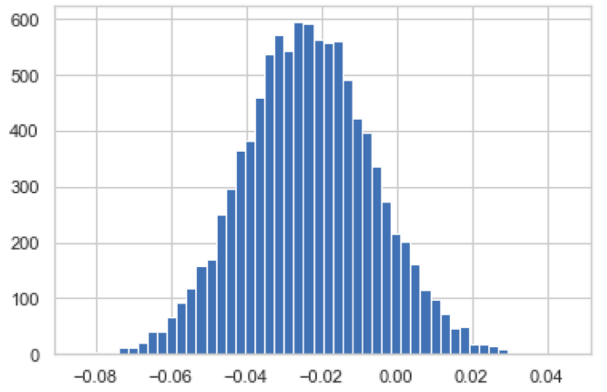

BD_3pr1_dif
P_H0_3pr1_dif 0.0886

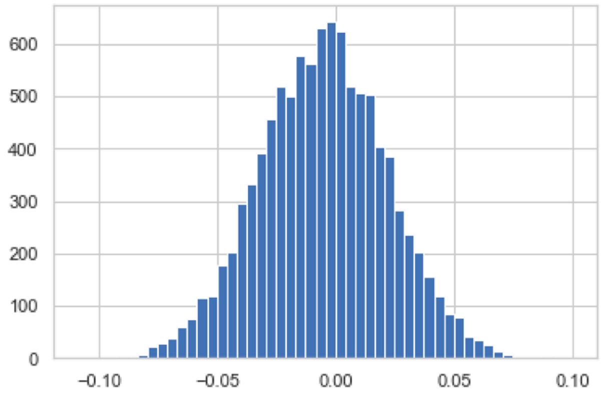

BD_3pr2_dif
P_H0_3pr2_dif 0.4189

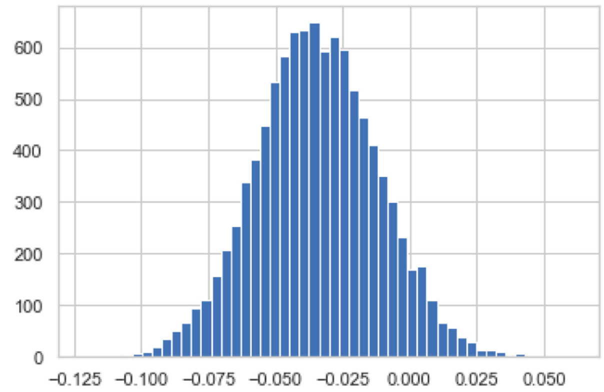

BD_3pr3_WC
P_H0_3pr3_WC 0.0656

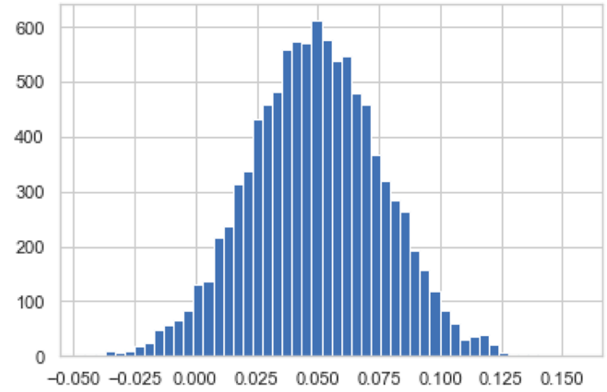

BD_3pr3_iso
P_H0_3pr3_iso 0.0357

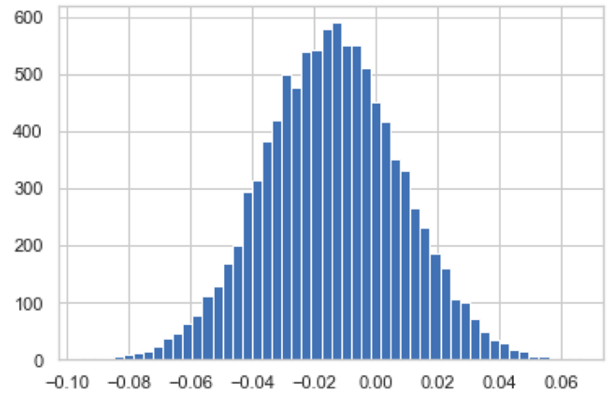

BD_3pr3_dif
P_H0_3pr3_dif 0.2606

Figure S8| Histograms of bootstrap difference for U5 bp types at each position of the exon junctions between +5Gsub and +5G datasets (Violinplots of the same **Figure 6A-C**)
BD = Distributions of **B**ootstrap **D**ifferences of the frequencies for U5 base pair types
Base pair types: **W**atson-**C**rick, **WC**; **iso**steric, **iso**; and non-isosteric (**dif**ferent), **dif**
Splice junction positions key: **5pr1** is 5'exon position -1; **3pr1** is 3'exon position +1

Histograms of bootstrap difference for U6 bp geometry in introns preceded by -1Gsub and -1G exons

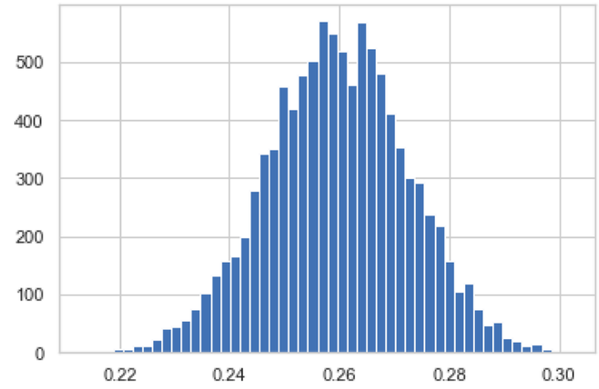

BD_in5_WC
P_H0_in5_WC 0.0

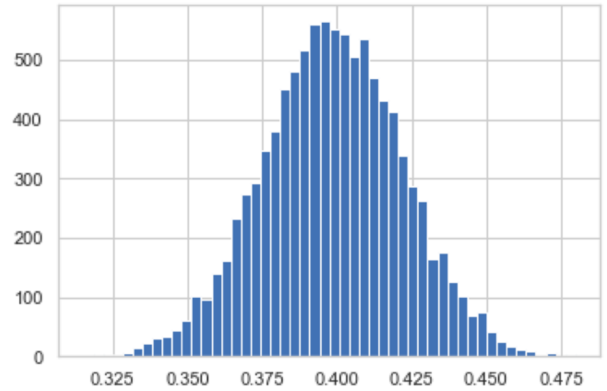

BD_in6_WC
P_H0_in6_WC 0.0

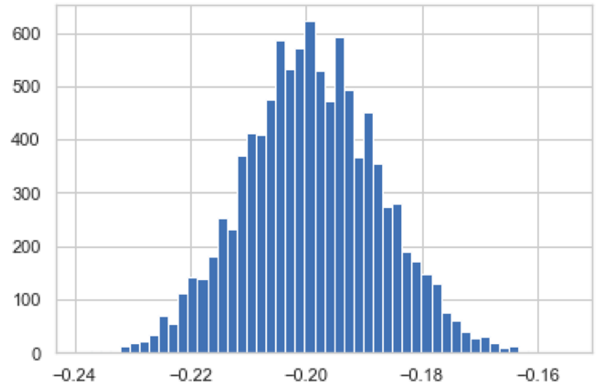

BD_in5_iso
P_H0_in5_iso 0.0

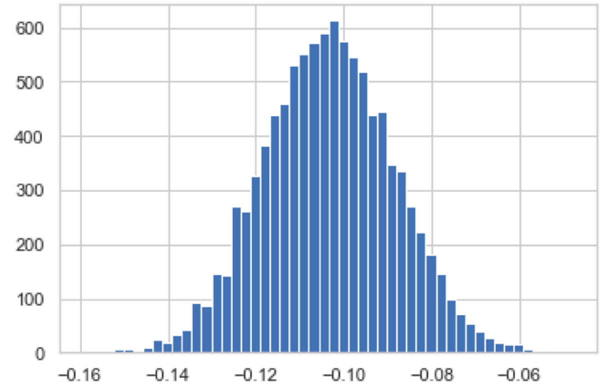

BD_in6_iso
P_H0_in6_iso 0.0

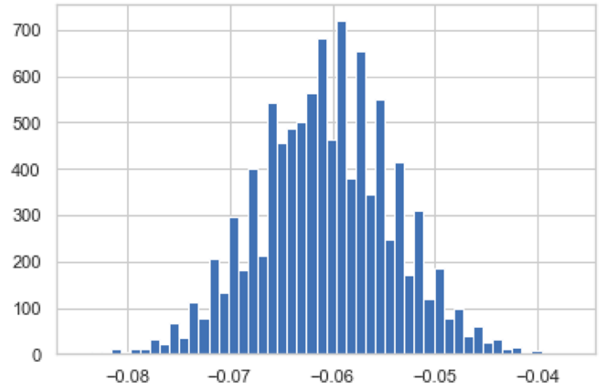

BD_in5_dif
P_H0_in5_dif 0.0

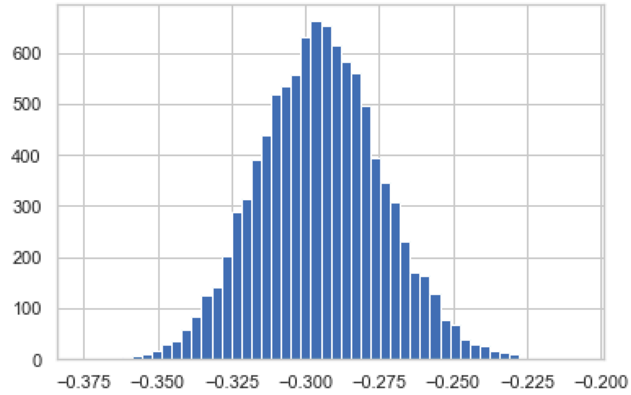

BD_in6_dif
P_H0_in6_dif 0.0

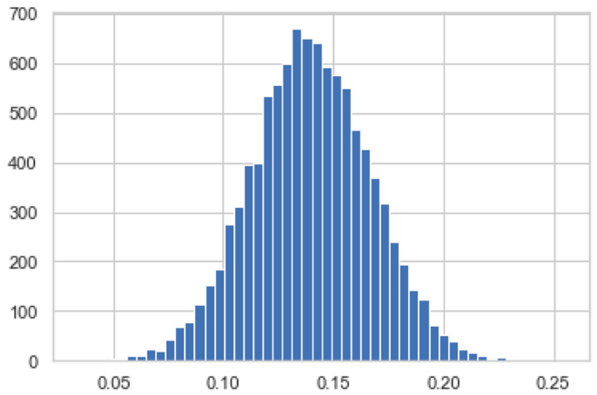

BD_in7_WC
P_H0_in7_WC 0.0

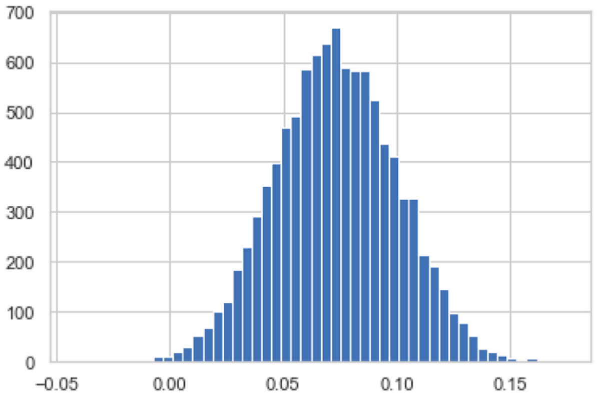

BD_in8_WC
P_H0_in8_WC 0.0029

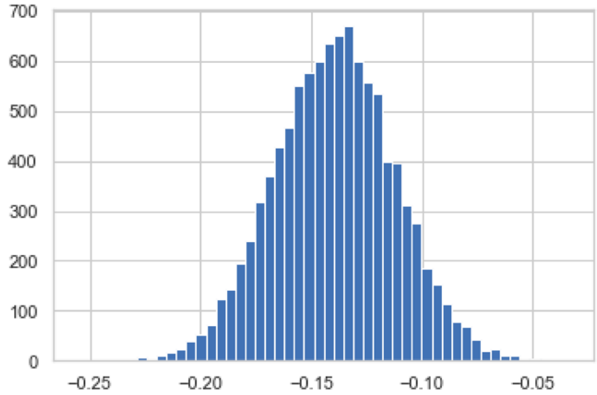

BD_in7_iso
P_H0_in7_iso 0.0

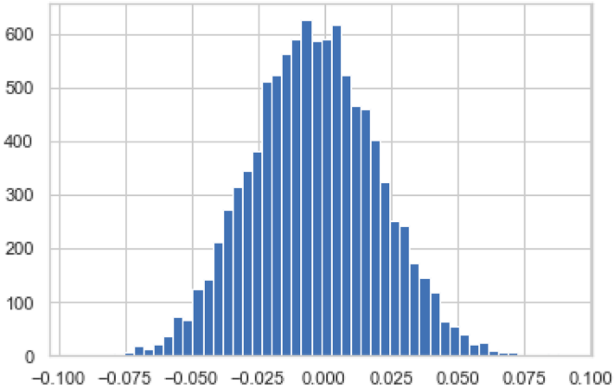

BD_in8_iso
P_H0_in8_iso 0.434

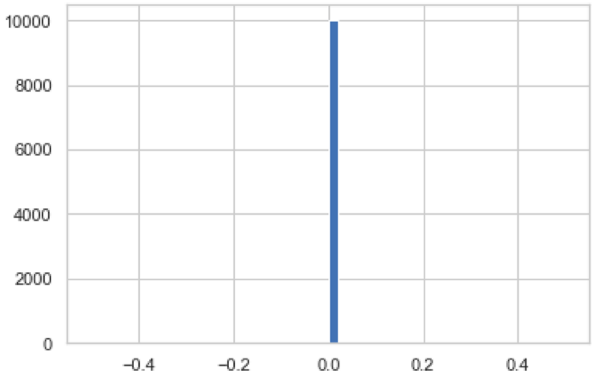

BD_in7_dif

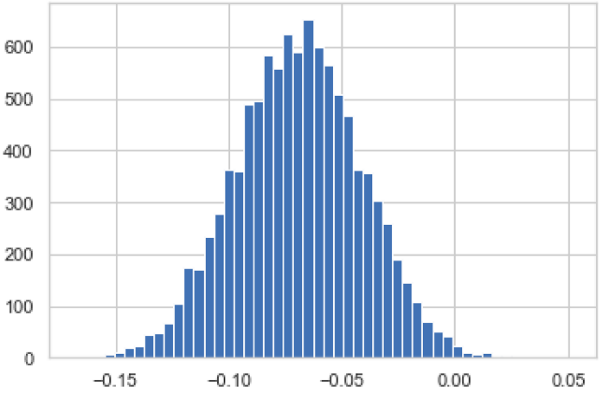

BD_in8_dif
P_H0_in8_dif 0.0051

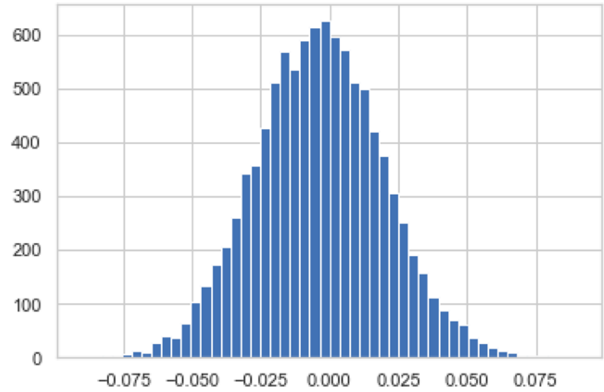

BD_in9_WC
P_H0_in9_WC 0.4314

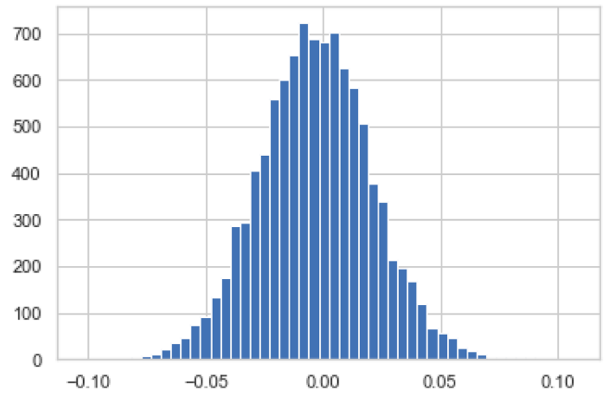

BD_in10_WC
P_H0_in10_WC 0.4462

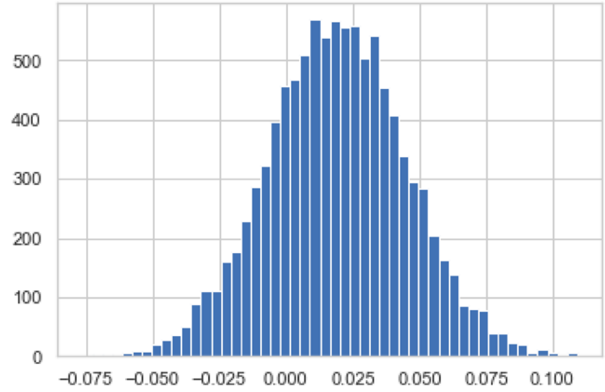

BD_in9_iso
P_H0_in9_iso 0.2326

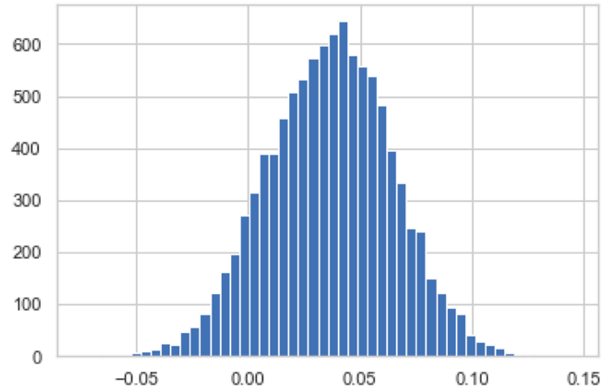

BD_in10_iso
P_H0_in10_iso 0.0993

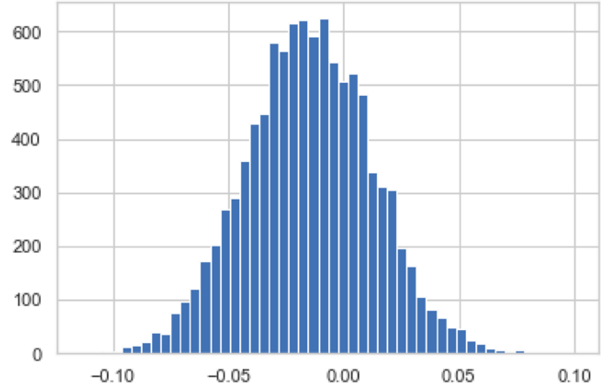

BD_in9_dif
P_H0_in9_dif 0.2933

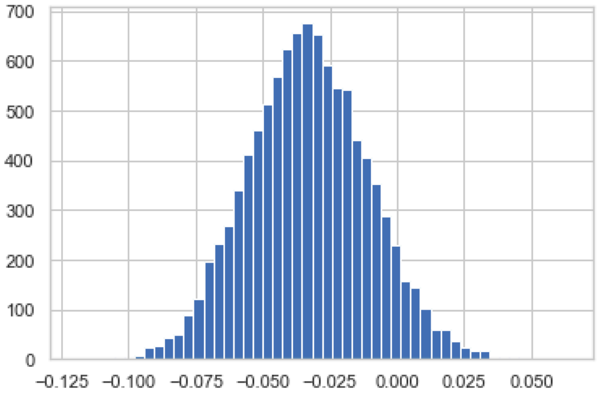

BD_in10_dif
P_H0_in10_dif 0.0717

Figure S9| Histograms of bootstrap difference for U6 bp types at the start of intron position +5 to +10 between -1Gsub and -1G datasets (-1G=exon-end G; Violinplots of the same **Figure 6D-F**)
Base pair types: **W**atson-**C**rick, **WC**; **iso**steric, **iso**; and non-isosteric (**dif**ferent), **dif**

Histograms of bootstrap difference for U5 bp geometry at exon junctions of -3Csub and -3C introns

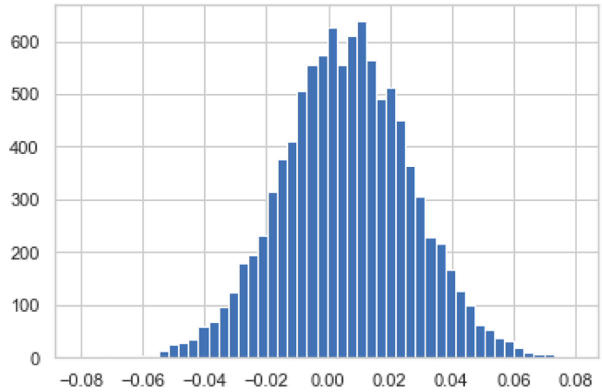

BD_5pr8_WC
P_H0_5pr8_WC 0.3906

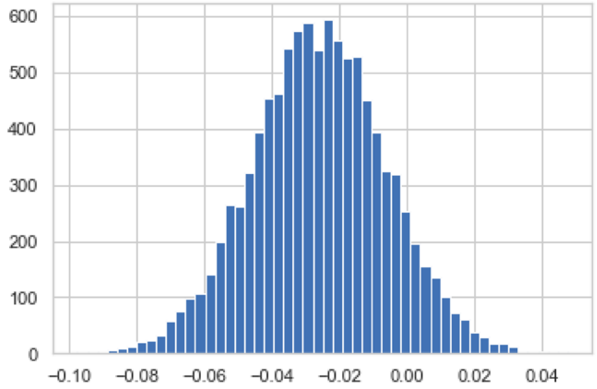

BD_5pr7_WC
P_H0_5pr7_WC 0.0944

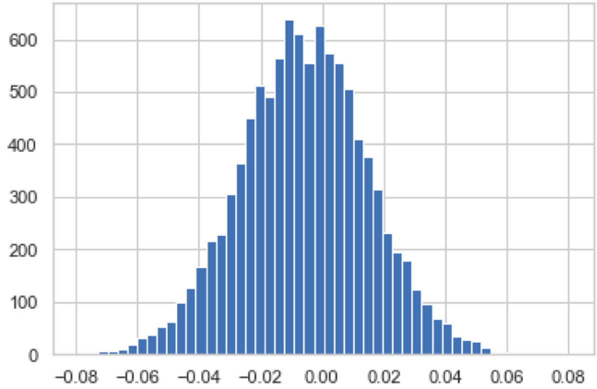

BD_5pr8_iso
P_H0_5pr8_iso 0.3906

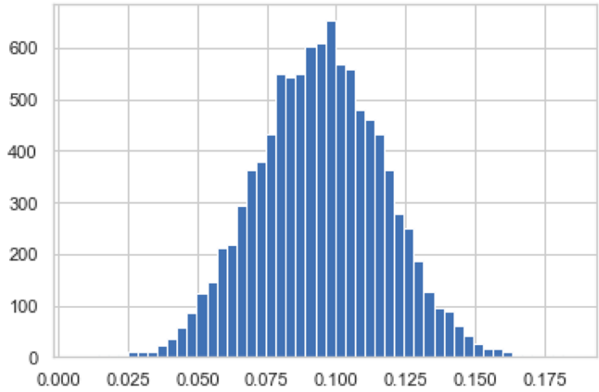

BD_5pr7_iso
P_H0_5pr7_iso 0.0

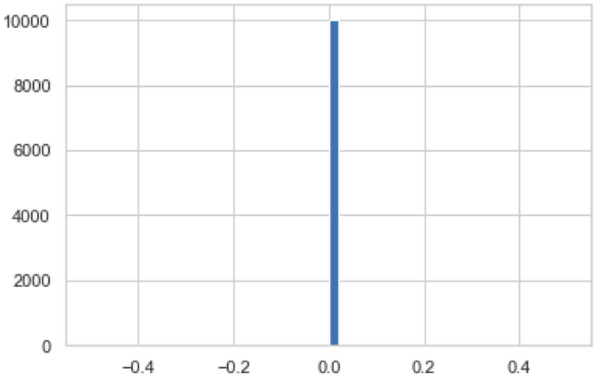

BD_5pr8_dif

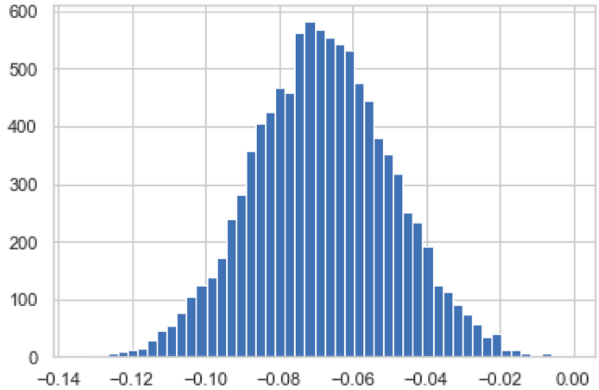

BD_5pr7_dif
P_H0_5pr7_dif 0.0

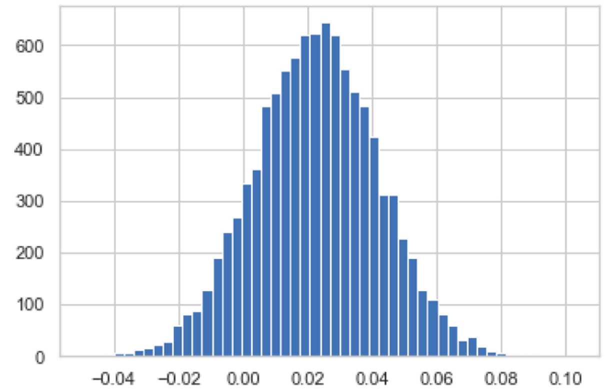

BD_5pr6_WC
P_H0_5pr6_WC 0.1224

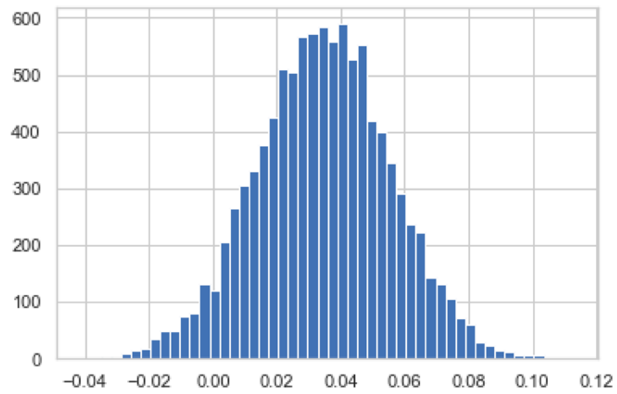

BD_5pr5_WC
P_H0_5pr5_WC 0.0518

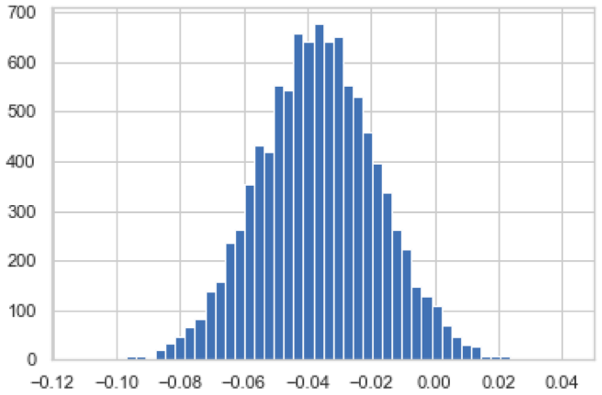

BD_5pr6_iso
P_H0_5pr6_iso 0.0268

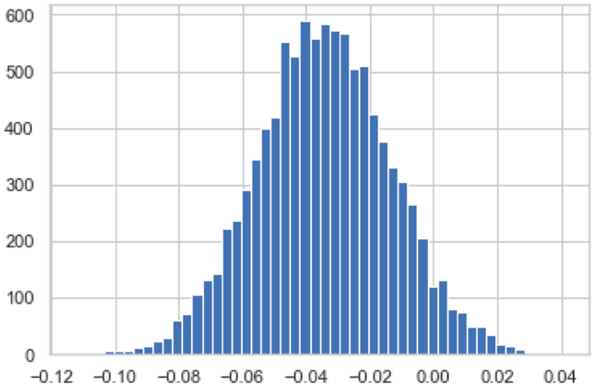

BD_5pr5_iso
P_H0_5pr5_iso 0.0518

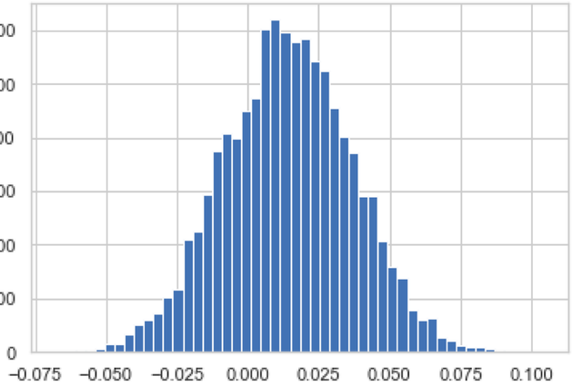

BD_5pr6_dif
P_H0_5pr6_dif 0.2722

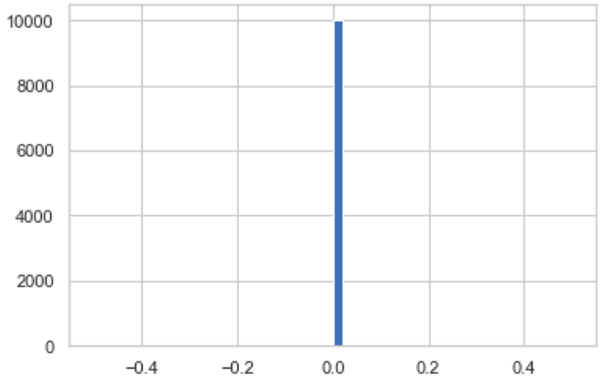

BD_5pr5_dif

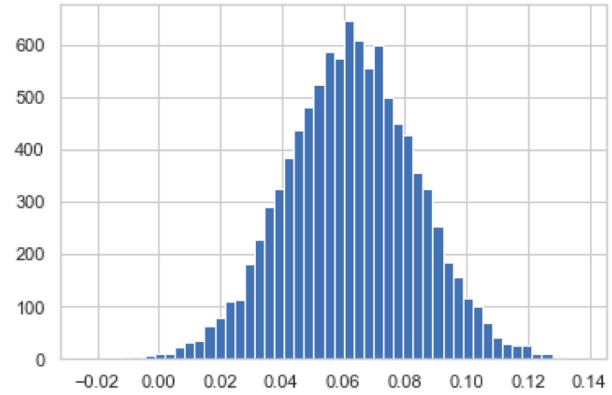

BD_5pr4_WC
P_H0_5pr4_WC 0.002

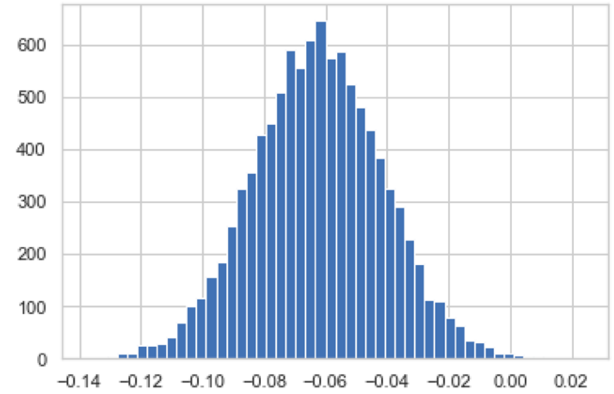

BD_5pr4_iso
P_H0_5pr4_iso 0.002

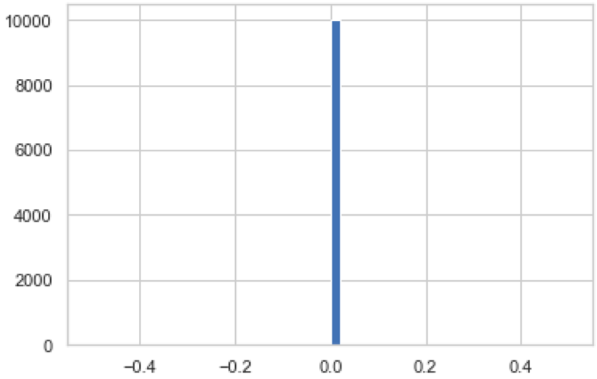

BD_5pr4_dif

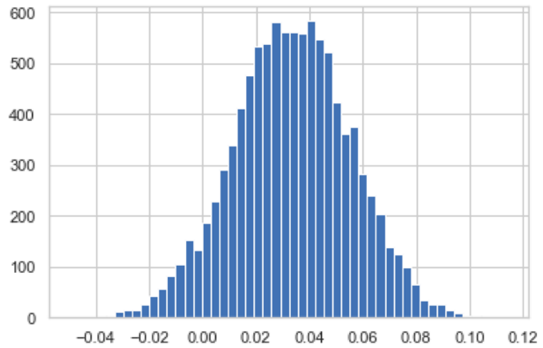

BD_5pr3_WC
P_H0_5pr3_WC 0.0659

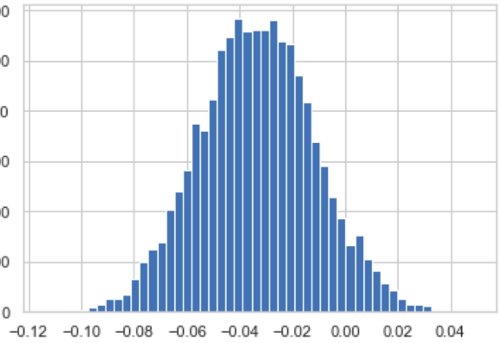

BD_5pr3_iso
P_H0_5pr3_iso 0.0659

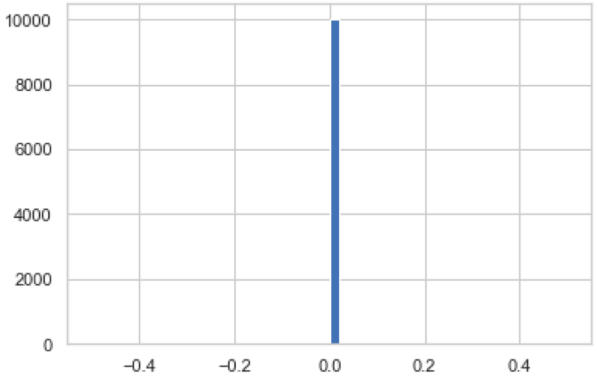

BD_5pr3_dif

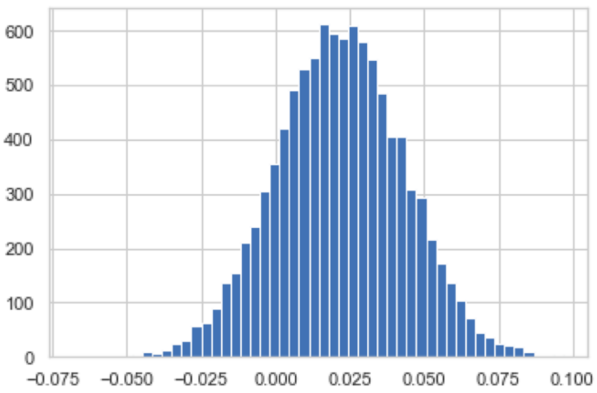

BD_5pr2_WC
P_H0_5pr2_WC 0.1551

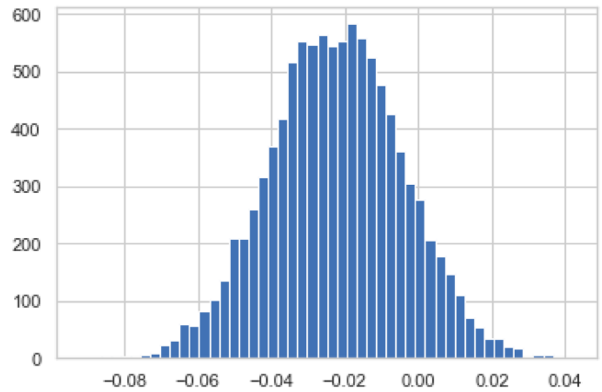

BD_5pr1_WC
P_H0_5pr1_WC 0.1067

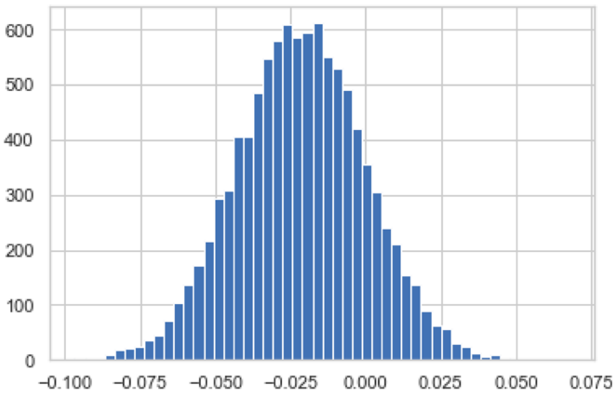

BD_5pr2_iso
P_H0_5pr2_iso 0.1551

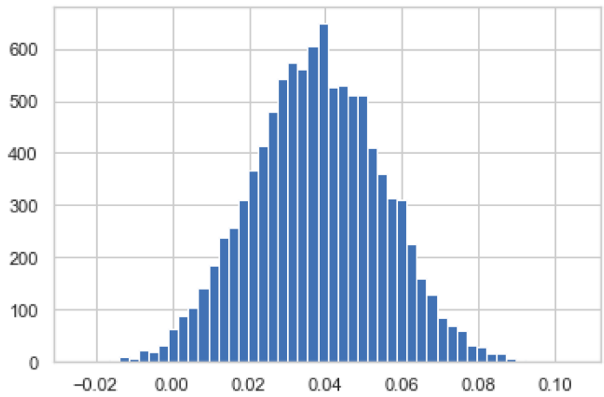

BD_5pr1_iso
P_H0_5pr1_iso 0.0119

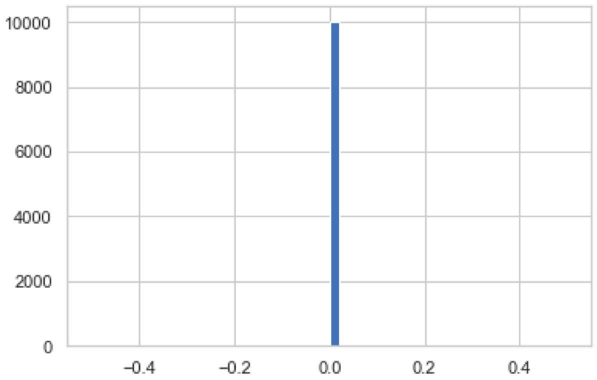

BD_5pr2_dif

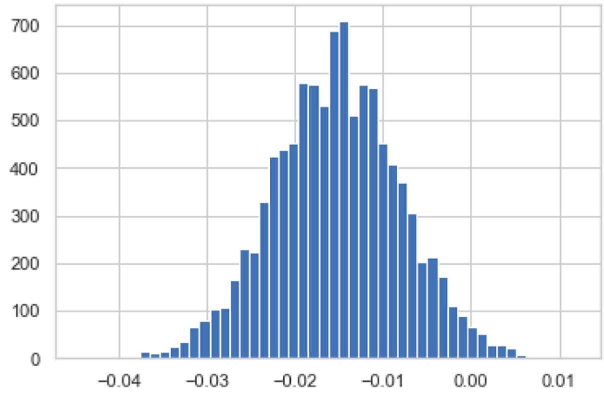

BD_5pr1_dif
P_H0_5pr1_dif 0.0194

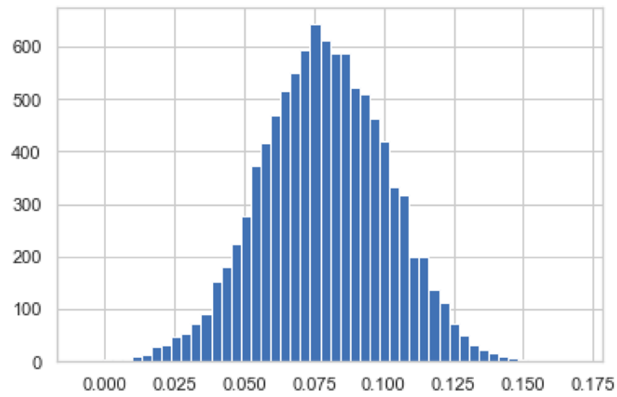

BD_3pr1_WC
P_H0_3pr1_WC 0.0001

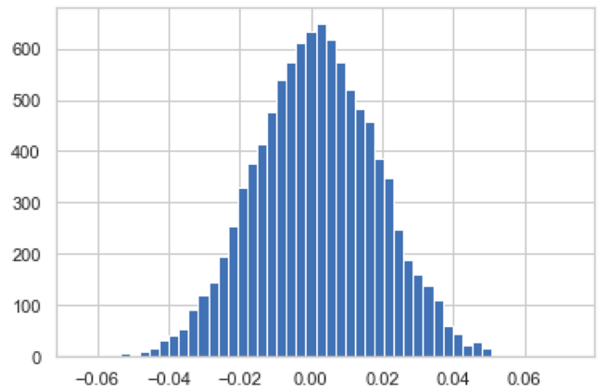

BD_3pr2_WC
P_H0_3pr2_WC 0.464

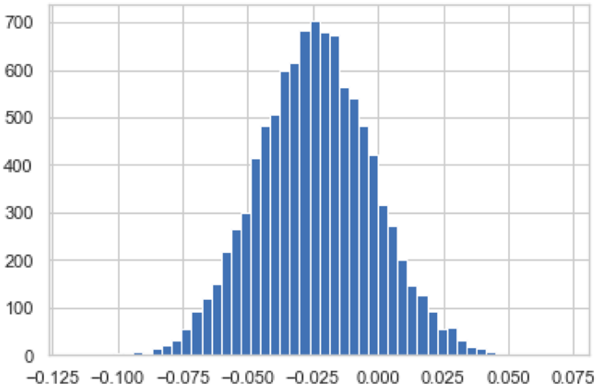

BD_3pr1_iso
P_H0_3pr1_iso 0.1345

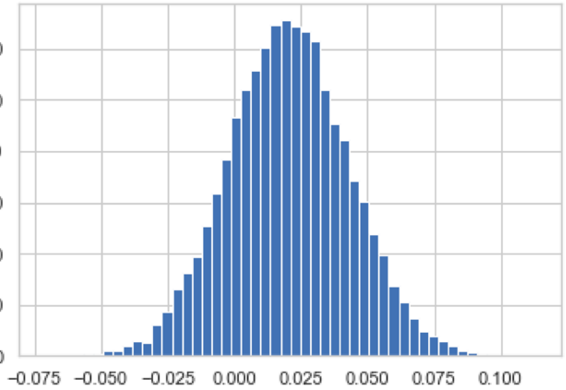

BD_3pr2_iso
P_H0_3pr2_iso 0.1825

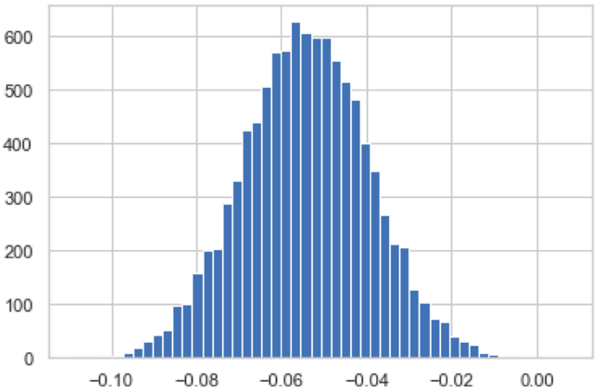

BD_3pr1_dif
P_H0_3pr1_dif 0.0002

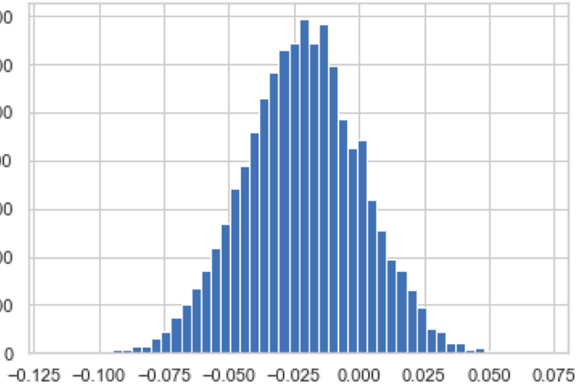

BD_3pr2_dif
P_H0_3pr2_dif 0.1712

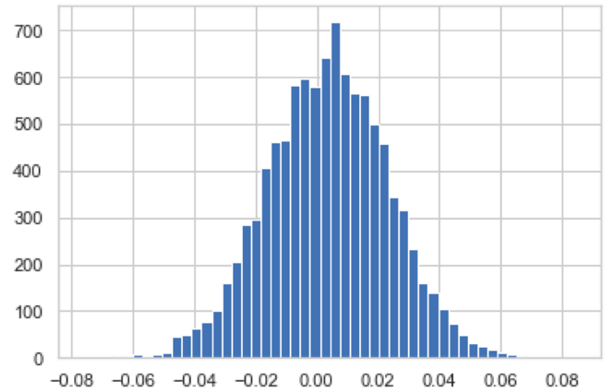

BD_3pr3_WC
P_H0_3pr3_WC 0.4227

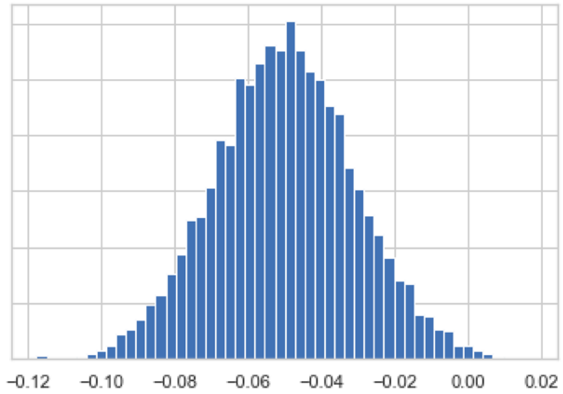

BD_3pr3_dif
P_H0_3pr3_dif 0.0043

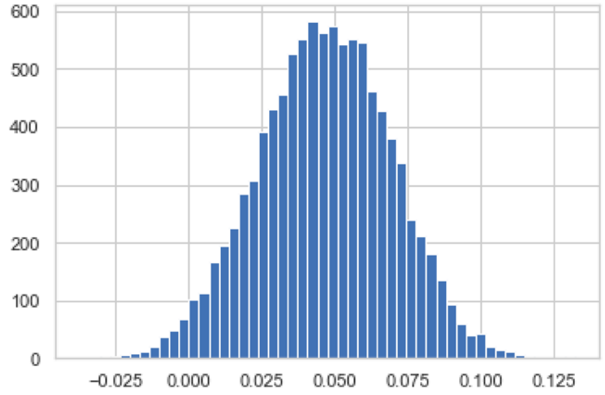

BD_3pr3_iso
P_H0_3pr3_iso 0.0212

Figure S10| Histograms of bootstrap difference for U5 bp types at each position of the splice junction between -3Csub and -3C datasets (Violinplots of the same **Figure 9D-F**)

Base pair types: **<u>W</u>**atson-**<u>C</u>**rick, **WC**; **<u>iso</u>**steric, **iso**; and non-isosteric (**<u>dif</u>**ferent), **dif**
Splice junction positions key: **5pr1** is 5'exon position -1; **3pr1** is 3'exon position +1
